# *Candida albicans* induces neutrophil extracellular traps and leucotoxic hypercitrullination via candidalysin

**DOI:** 10.15252/embr.202357571

**Published:** 2023-10-05

**Authors:** Lucas Unger, Samuel Skoluda, Emelie Backman, Borko Amulic, Fernando M Ponce‐Garcia, Chinelo NC Etiaba, Sujan Yellagunda, Renate Krüger, Horst von Bernuth, Johan Bylund, Bernhard Hube, Julian R Naglik, Constantin F Urban

**Affiliations:** ^1^ Department of Clinical Microbiology Umeå University Umeå Sweden; ^2^ Umeå Centre for Microbial Research (UCMR) Umeå University Umeå Sweden; ^3^ School of Cellular and Molecular Medicine University of Bristol Bristol UK; ^4^ Department of Pediatric Respiratory Medicine, Immunology and Critical Care Medicine Charité – Universitätsmedizin Berlin Berlin Germany; ^5^ Department of Immunology Labor Berlin Labor Berlin – Charité Vivantes GmbH Berlin Germany; ^6^ Berlin Institute of Health at Charité – Universitätsmedizin Berlin Berlin Germany; ^7^ Charité – Universitätsmedizin Berlin, Corporate Member of Freie Universität Berlin, Humboldt‐Universität zu Berlin, and Berlin Institute of Health (BIH) Berlin‐Brandenburg Center for Regenerative Therapies (BCRT) Berlin Germany; ^8^ Department of Oral Microbiology & Immunology, Institute of Odontology Sahlgrenska Academy at University of Gothenburg Gothenburg Sweden; ^9^ Department of Microbial Pathogenicity Mechanisms Leibniz Institute for Natural Product Research and Infection Biology ‐ Hans‐Knoell‐Institute Jena Germany; ^10^ Friedrich Schiller University Jena Germany; ^11^ Centre for Host‐Microbiome Interactions, Faculty of Dentistry, Oral & Craniofacial Sciences King's College London London UK

**Keywords:** chronic granulomatous disease, fungal immunology, histone citrullination, polymorphonuclear leucocytes, reactive oxygen species, Immunology, Microbiology, Virology & Host Pathogen Interaction, Post-translational Modifications & Proteolysis

## Abstract

The peptide toxin candidalysin, secreted by *Candida albicans* hyphae, promotes stimulation of neutrophil extracellular traps (NETs). However, candidalysin alone triggers a distinct mechanism for NET‐like structures (NLS), which are more compact and less fibrous than canonical NETs. Candidalysin activates NADPH oxidase and calcium influx, with both processes contributing to morphological changes in neutrophils resulting in NLS formation. NLS are induced by leucotoxic hypercitrullination, which is governed by calcium‐induced protein arginine deaminase 4 activation and initiation of intracellular signalling events in a dose‐ and time‐dependent manner. However, activation of signalling by candidalysin does not suffice to trigger downstream events essential for NET formation, as demonstrated by lack of lamin A/C phosphorylation, an event required for activation of cyclin‐dependent kinases that are crucial for NET release. Candidalysin‐triggered NLS demonstrate anti‐*Candida* activity, which is resistant to nuclease treatment and dependent on the deprivation of Zn^2+^. This study reveals that *C. albicans* hyphae releasing candidalysin concurrently trigger canonical NETs and NLS, which together form a fibrous sticky network that entangles *C. albicans* hyphae and efficiently inhibits their growth.

## Graphical abstract

The cytolytic peptide toxin candidalysin is secreted by the invasive hyphal form of the human fungal pathogen *Candida albicans*. This study investigates the intricate interaction of candidalysin and neutrophils.

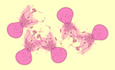

## Introduction

Neutrophils are important innate immune cells that play a crucial role in preventing fungal infections (Bianchi *et al*, 2009). In addition to engulfing and eradicating microbes by phagocytosis, extracellular mechanisms involving the release of neutrophil extracellular traps (NETs) and granular vesicles have been described (Ermert *et al*, 2009; Branzk *et al*, 2014; Shopova *et al*, 2020). As pathogenic fungi can grow as a network of filamentous hyphae, phagocytic killing by neutrophils is often insufficient, thus extracellular mechanisms, such as NET formation, are required for efficient eradication. NETs have been reported to restrict fungal growth and corroborate inflammatory responses during mycoses (Bianchi *et al*, 2009; Urban *et al*, 2009; Khandagale *et al*, 2018). Pathogenic fungi trigger NETs in an NADPH oxidase‐dependent manner involving activation of cyclin‐dependent kinases 4 and 6 (CDK4/6) (Amulic *et al*, 2017). Several studies indicate that if NET release is not properly balanced, NETs may also have harmful effects on the host, mainly due to their pro‐inflammatory function (Brinkmann & Zychlinsky, 2012). Notably, microbial toxins can trigger leucotoxic hypercitrullination of histones in neutrophils resulting in similar extracellular structures, termed NET‐like structures (NLS) (Konig & Andrade, 2016; Bjornsdottir *et al*, 2017). NLS are less fibrous and more compact than canonical NETs and are triggered in an NADPH oxidase‐independent fashion. Similar to NETs, NLS can induce pro‐inflammatory effects with potentially hazardous consequences for the host (Neeli *et al*, 2008; Wang *et al*, 2009; Konig & Andrade, 2016).

The human fungal pathogen, *Candida albicans*, is a dimorphic yeast with the ability to form invasive, filamentous hyphae (Chow *et al*, 2021). The yeast–hyphal transition, in combination with the expression of hypha‐associated factors, is critical for *C. albicans* virulence (Jacobsen *et al*, 2012; Mayer *et al*, 2013). Invasive *C. albicans* hyphae are controlled by human neutrophils, thereby preventing dissemination and exacerbation of disease in otherwise healthy patients (Ermert *et al*, 2013). A critical factor for the invasive and inflammatory potential of *C. albicans* hyphae is the recently discovered peptide toxin candidalysin (Moyes *et al*, 2016; Kasper *et al*, 2018). Candidalysin is released from the polyprotein Ece1p via a sequential proteolytic cleavage by the proteases Kex2p and Kex1p (Bader *et al*, 2008). The corresponding *ECE1* gene is exclusively expressed by the hyphal morphology of *C. albicans* (Birse *et al*, 1993) and belongs to the hyphal core response genes consisting of eight hyphal‐associated genes expressed under a variety of hyphal‐inducing conditions (Martin *et al*, 2013). *C. albicans* hyphae deficient in candidalysin are unable to damage epithelial cells or activate key signalling mechanisms that result in alarmin release and inflammatory responses and the recruitment of neutrophils (Moyes *et al*, 2010, 2014; Ho *et al*, 2019). Consequently, neutrophil recruitment is severely impaired in models of mucosal and systemic candidiasis in response to candidalysin‐deficient mutant strains (Verma *et al*, 2017; Richardson *et al*, 2018; Drummond *et al*, 2019; Swidergall *et al*, 2019). Thus, we investigated whether candidalysin can directly act on neutrophils and whether the toxin shapes neutrophil responses, which in turn may impact the outcome of invasive candidiasis.

We found that candidalysin‐expressing *C. albicans* strains induced more NETs than candidalysin‐deficient strains, indicating that candidalysin amplifies NET formation. Notably, candidalysin as exclusive stimulus induced leucotoxic hypercitrullination and the release of NLS. In contrast to previously described stimuli of NLS, candidalysin induced NLS in partial dependence on NADPH oxidase‐mediated reactive oxygen species (ROS) production, while PAD4‐mediated histone citrullination could be observed as well. Notably, candidalysin alone failed to induce NETs as indicated by a lack of cell cycle activation determined via lamin A/C phosphorylation assays. Our data reveal that candidalysin is a critical virulence factor shaping neutrophil responses, which are essential for antifungal immunity.

## Results

### Candidalysin contributes to *C. albicans*‐induced NET formation

**Figure 1:**
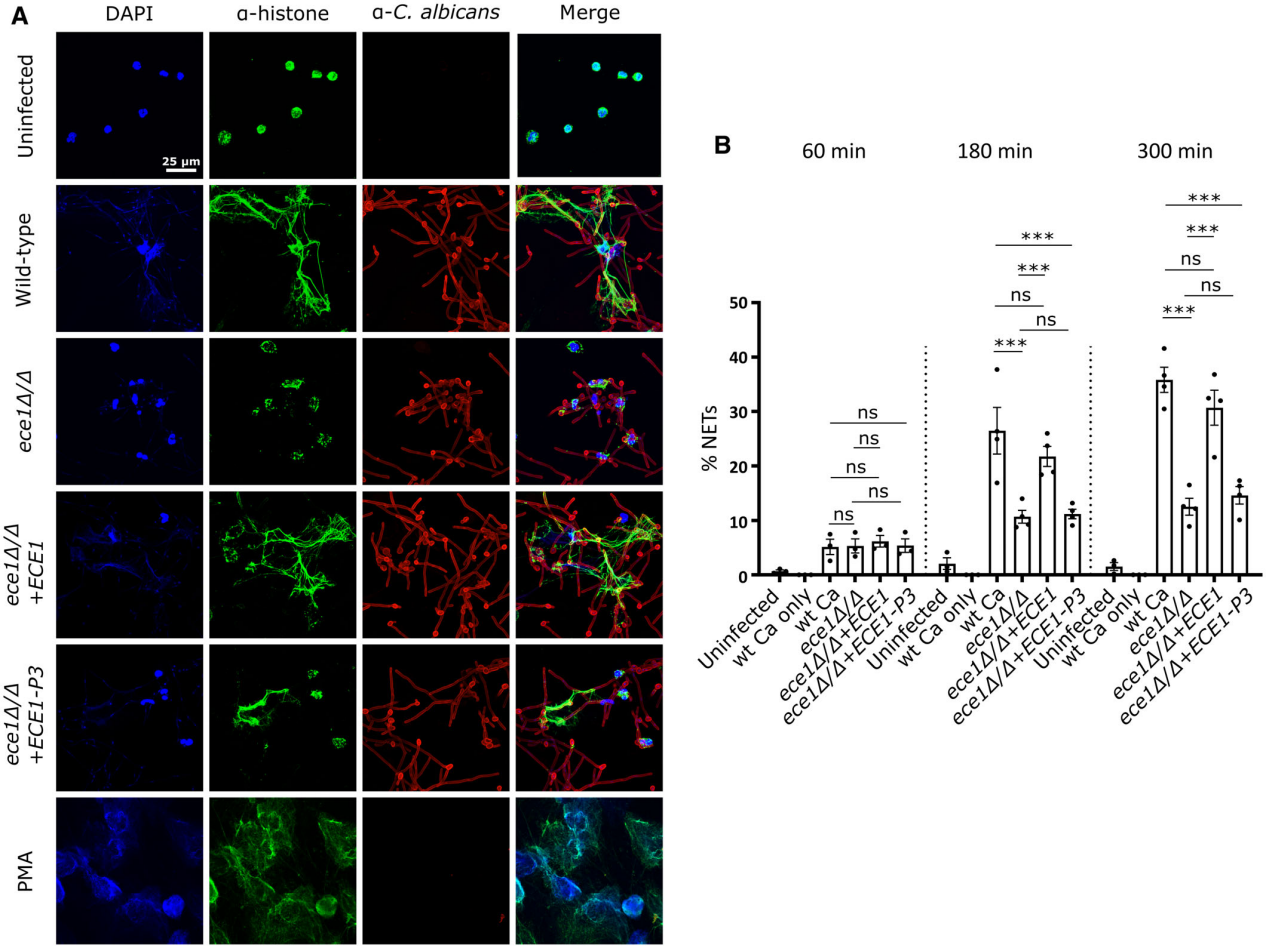
Candidalysin promotes NET formation induced by *C. albicans* hyphae Representative images of confocal immunofluorescence microscopy (60×) show human neutrophils 4 h after infection with wild‐type and candidalysin‐deleted *C. albicans* strains (*ece1*Δ/Δ and *ece1*Δ/Δ+*ECE1*‐P3). *C. albicans* were visualized using anti‐*C. albicans* antibody staining. Lack of Ece1p/candidalysin production led to reduced NET formation as visualized by chromatin staining using anti‐α‐histone antibody staining. Scale bar: 25 μm.Visual impression was corroborated with quantitative image analysis of a time‐series experiment using ImageJ (*n* = 4 (10–14)). Each DAPI‐stained event exceeding 100 μm^2^ was considered a NET. Indicated per cent NET rate was normalized to total amount of DAPI events (cell count) per quantified image according to previous reports (Hosseinzadeh *et al*, 2012, 2016). Data information: Data in (B) is shown as mean ± SEM and statistically analysed using a two‐way ANOVA with Bonferroni *post hoc* test. Representative microscopic images were not obtained from the same experiment conducted for quantification due to different immunostaining procedures. Stars above bars indicate ***P < 0.001 and “ns” indicates “not significant.” If not stated otherwise, numbers of biological replicates using independent neutrophil donors in separate experiments are indicated in the figure legends as *n* = biological replicate number (technical replicate number within each individual experiment). Source data are available online for this figure.

Neutrophils release NETs as a defence mechanism in response to *C. albicans* infections, particularly to control filamentous hyphae that are difficult to phagocytose (Urban *et al*, 2006, 2009; Ermert *et al*, 2009). *C. albicans* hyphae release candidalysin and while the effects of the toxin, for instance, on virulence in general and on adhesion to host cells have been widely studied (Moyes *et al*, 2016; Kasper *et al*, 2018; Ho *et al*, 2019; Swidergall *et al*, 2019; Mogavero *et al*, 2021), the direct impact of candidalysin on the neutrophil immune response towards *C. albicans* remains poorly understood. To investigate the role of candidalysin, we infected neutrophils with wild‐type *C. albicans*, *ECE1*‐deficient (*ece1*ΔΔ) and corresponding revertant (*ece1*ΔΔ+*ECE1*) strains, and a strain only lacking the candidalysin‐coding sequence (P3) within the *ECE1* gene (*ece1*ΔΔ+*ECE1*‐P3). After 4 h of infection, samples were prepared for indirect immunofluorescence microscopy to visualize extracellular trap events using decondensed neutrophil chromatin (DNA and α‐histone) as marker (Fig 1). Whereas wild‐type and the revertant strain induced comparable amounts of NETs, the *ECE1*‐ and candidalysin‐deficient strains triggered strongly reduced levels (Fig 1A). Based on previously published image‐based quantitative analysis of NET formation (Hosseinzadeh *et al*, 2012, 2016), each DAPI‐stained event exceeding 100 μm^2^ was considered a NET. The quantification revealed that both toxin‐deleted strains induced significantly less NETs compared to toxin‐expressing strains, with ~60% decreased levels after 3 and 5 h compared with the wild type (Fig 1B). Notably, the scrutinized image‐based quantification excluded background noise potentially derived from cell debris as confirmed by unstimulated control samples which were incubated in the same manner as stimulated samples (Fig 1B). In conclusion, the data demonstrate that candidalysin contributes to NET formation triggered by *C. albicans* hyphae.

### Candidalysin induces NET‐like structures

**Figure 2:**
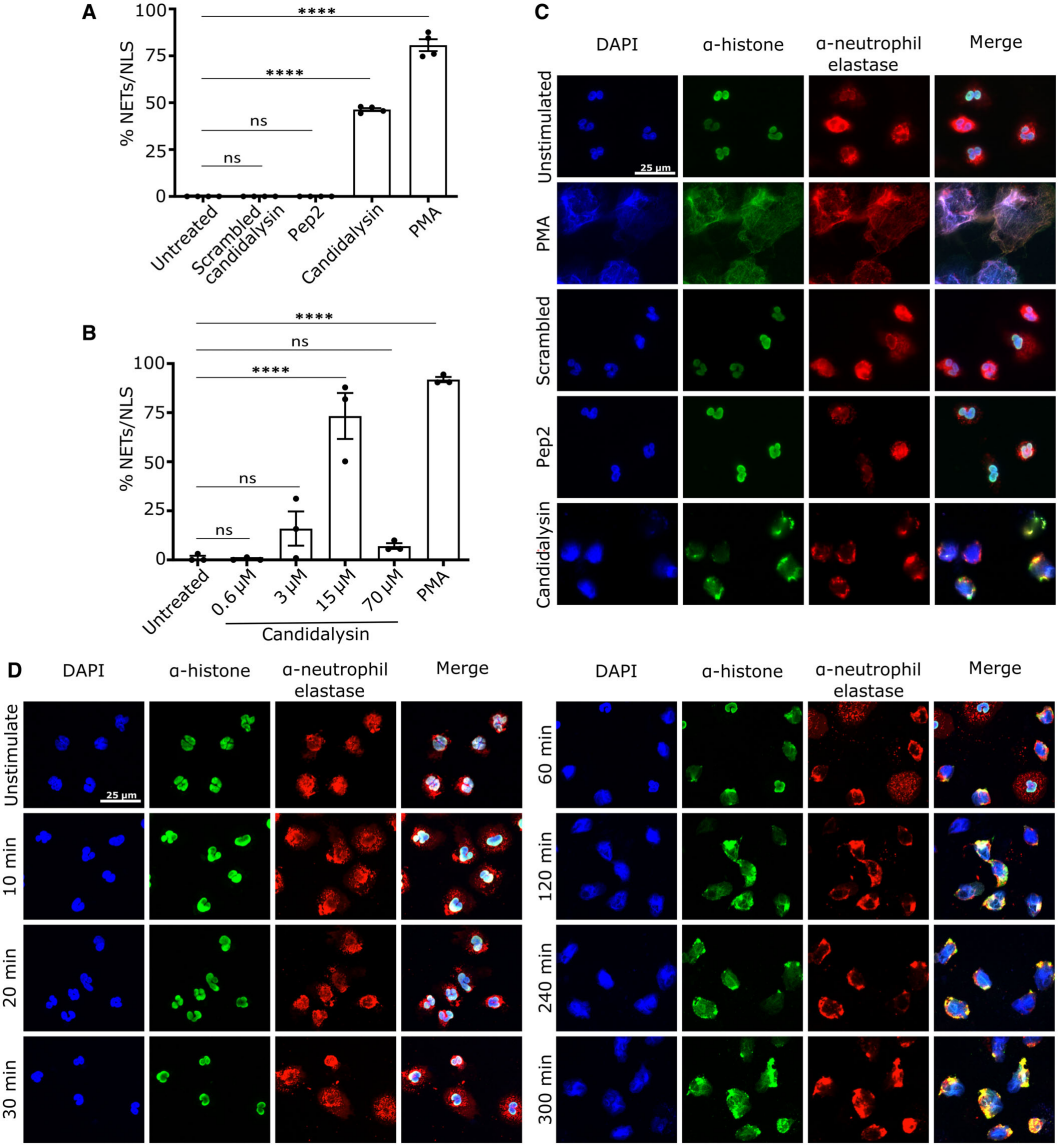
Candidalysin induces NLS in human neutrophils Candidalysin, but not scrambled candidalysin or pep2, another Ece1p‐derived peptide (all 15 μM), induced DNA decondensation in human neutrophils after 4 h (*n* = 4 (10–14)) as determined by quantitative image analysis. To allow comparability, NLS were quantified with the same criteria as described for NETs.Similarly, candidalysin was used to stimulate neutrophils in a dose‐dependent manner and NLS were determined using quantitative image analysis (*n* = 3 (10–14)).Representative images of confocal immunofluorescence microscopy display morphological changes in neutrophils involving nuclear and granular proteins after 4 h compared to unstimulated cells or 100 nM PMA, or cells exposed to scrambled candidalysin and pep2. The morphological changes evoked by PMA considerably deviated from morphological changes evoked by candidalysin and, hence, were defined as NETs (for PMA) and NLS (for candidalysin). Scale bar: 25 μm.Representative images of confocal immunofluorescence microscopy show time‐dependent progression of morphological changes in neutrophils induced by candidalysin over the course of 5 h. Scale bar: 25 μm. Data information: Data in (A, B) shown as mean ± SEM and statistically analysed using one‐way ANOVA with Bonferroni *post hoc* test. Stars above bars indicate ****P < 0.0001 and “ns” indicates “not significant.” All images are with 60× magnification. Source data are available online for this figure.

**Figure EV1:**
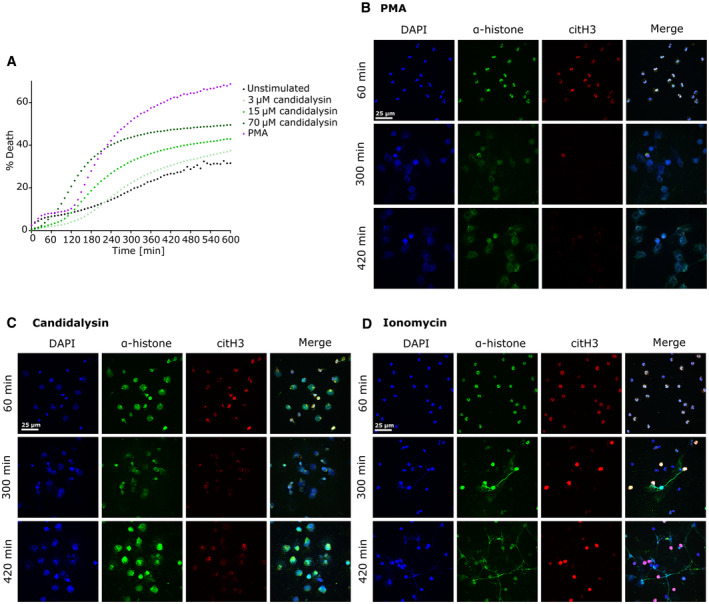
Candidalysin has dose‐dependent effects on human neutrophils ASytox Green staining demonstrated dose‐ and time‐dependent cytotoxic effects of candidalysin on neutrophils (one representative of four biological replicates shown with *n* = 4 technical replicates).B–DRepresentative images of confocal immunofluorescence microscopy depict neutrophils that were treated with (B) 100 nM PMA, (C) 15 μM candidalysin and (D) ionomycin at 1, 3, 5 and 7 h incubation time. Progressing chromatin expansion and release of DNA patches or fibres can be observed over time. Data information: (A) Data presented as mean of percentage of a Triton‐X‐treated lysis control. (B–D) Visualization accomplished by staining DNA with DAPI (blue channel), primary antibody directed against histones (green channel), and citrullinated histone 3 (citH3). Images taken with Nikon A1R confocal microscope (LSM) controlled by Nikon NIS elements interface with a Nikon Eclipse Ti‐E inverted microscope using 60× magnification. If not stated otherwise, numbers of biological replicates using independent neutrophil donors in separate experiments are indicated in the figure legends – *n* = biological replicate number (technical replicate number within each individual experiment).

As candidalysin‐expressing *C. albicans* strains induced more NETs than candidalysin‐deficient strains, we investigated the role of the toxin alone in stimulating neutrophil extracellular trap release. Exposure of neutrophils to candidalysin was sufficient to trigger morphological changes (chromatin decondensation) in 46.3 ± 0.8% of cells after 4 h compared to 80.7 ± 3.2% after exposure to PMA, a well‐known inducer of NETs (Fig 2A). Neither scrambled candidalysin nor Ece1p peptide 2 (one of eight different Ece1p‐derived peptides) affected neutrophil morphology, confirming specificity to candidalysin. Neutrophil chromatin decondensation via candidalysin was also dose dependent, as decondensation increased from 3 μM candidalysin to 15 μM candidalysin (Fig 2B). Notably, the outspread structures in response to candidalysin were more compact, less fibrous and patchier compared to canonical NETs released upon stimulation with PMA or *C. albicans* hyphae (compare Figs 2C and D, and 1A wild type, respectively). Hence, we concluded that candidalysin did not stimulate canonical NETs, but rather more compact DNA structures, resembling NLS that may be the result of leucotoxic hypercitrullination (Wang *et al*, 2009; Neeli & Radic, 2013). In order to ensure consistency in NET/NLS quantification, NLS were quantified with the same criteria as previous described for NETs. Candidalysin demonstrated a dose‐dependent effect with increased NLS formation from 3 to 15 μM. However, reduced NLS formation was observed at 70 μM (Fig 2B), which could result from rapid neutrophil cell death induced by the toxin as determined by a DNA Sytox Green assay (Fig EV1A). The structures induced by candidalysin were morphologically different from canonical NETs. However, the time course of morphological changes occurring during exposure to candidalysin was similar to the dynamics of morphological alterations during PMA‐induced or *C. albicans* hypha‐induced NET formation (Figs EV1B and 1A). In both cases, nuclear decondensation commenced at ~60 min and mixing of granular and nuclear components at ~120 min after stimulation (Figs 2D and EV1C). In summary, candidalysin alone triggered morphologically distinct NLS in a time‐ and dose‐dependent manner, whereas candidalysin‐producing *C. albicans* hyphae induced canonical NETs (Fig 1A).

**Figure 3:**
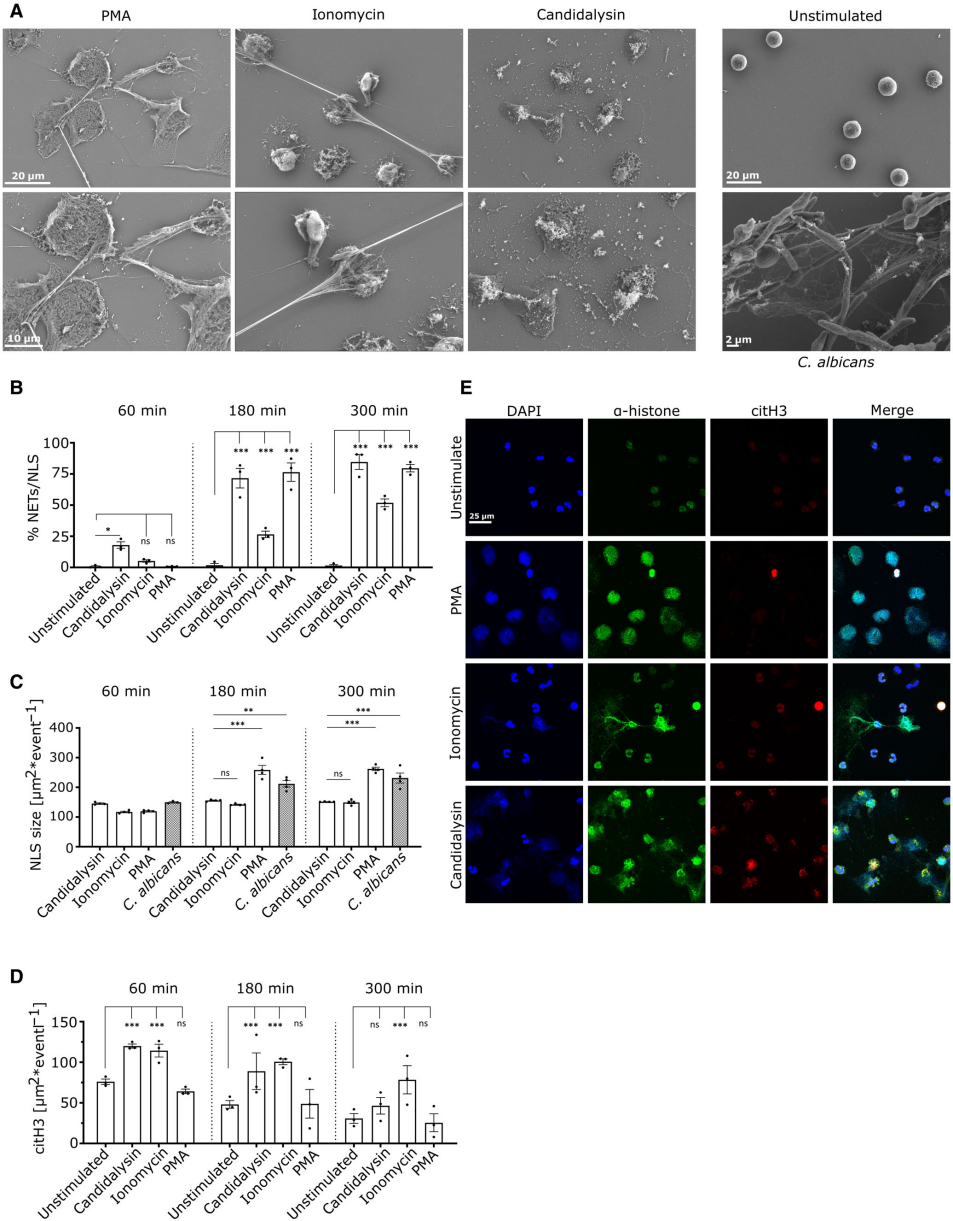
Morphological alterations triggered by candidalysin Scanning electron microscope images of candidalysin‐ (15 μM) and ionomycin‐ (4 μM) stimulated neutrophils after 3 h show differences in structural alterations compared to canonical NETs induced by PMA (100 nM) or by *C. albicans* hyphae (MOI 2) (treated neutrophils: magnification 3.00 KX on top, 5.00 KX at bottom, *C. albicans*‐infected neutrophils 4.00 KX and unstimulated neutrophils 2.3 KX). Scale bars as defined in images.Quantitative image analysis determines DNA decondensation as measure for NLS formation (*n* = 3 (10–14)).Quantitative image analysis determined average size of NLS formed per event for which only DNA‐stained area larger than 100 μm^2^ was considered (for candidalysin, ionomycin, and PMA stimulation *n* = 3 (10–14), for *C. albicans* stimulation *n* = 4 (10–14)).Quantitative image analysis determines histone citrullination level per event (*n* = 3 (10–14)).Representative images of confocal immunofluorescence microscopy 3 h after neutrophil stimulation support the quantitative data visually (60× magnification). Scale bar: 25 μm. Data information: Data in (B–D) are shown as mean ± SEM and statistically analysed using two‐way ANOVA with Bonferroni *post hoc* test. Stars above bars indicate *P < 0.05, **P < 0.01, ***P < 0.001; and “ns” indicates “not significant.” Source data are available online for this figure.

### Candidalysin‐induced NET‐like structures differ morphologically from NETs induced by various stimuli

To investigate candidalysin‐triggered NLS in further detail, we used scanning electron microscopy (SEM) which allows a more detailed view of the neutrophil‐derived structures (Fig 3A). To categorize the morphological alterations upon candidalysin stimulation, we compared the alterations with canonical ROS‐dependent NETs triggered by PMA and NLS upon exposure to the bacterial peptide toxin ionomycin. Ionomycin has been previously reported to induce NLS, also referred to as leucotoxic hypercitrullination (Wang *et al*, 2009; Neeli & Radic, 2013). PMA exposure generated widespread chromatin fibres in the extracellular space (Fig 3A, left panels), whereas ionomycin exposure resulted in more compact, patchy areas occasionally dispersed with long, thin chromatin fibres (Fig 3B, middle panels). With regard to morphological changes, candidalysin treatment resulted in compact, fibrous structures resembling those stemming from ionomycin treatment; however, long, thread‐like structures were absent in candidalysin‐treated neutrophil samples (Fig 3A right panels, for 7 h treatment see Fig EV1C). As expected, *C. albicans* hyphae induced NETs with observable fibres and threads similar to PMA‐induced canonical NETs (Fig 3A, lower right panel).

Image‐based quantification of NLS events (candidalysin and ionomycin) and NETs (PMA and *C. albicans* hyphae) revealed that although candidalysin‐triggered NLS appeared slightly earlier (after 1 h 17.9 ± 2.6% NLS), time dependency and quantity were similar compared to PMA‐induced NETs (Fig 3B). Ionomycin‐induced changes, however, were more delayed with 26.5 ± 2.6% and 51.9 ± 3.1% NLS after 3 and 5 h, respectively, and led to overall fewer NLS events. This was confirmed by an area‐based analysis of the events (Fig 3C). The average area per event exceeding 100 μm^2^ was determined using the images from the DNA stain. The frequency of extended threads was low for ionomycin‐treated samples and the average area was significantly smaller for ionomycin‐induced NLS (149.3 ± 6.21 after 3 h) than it was for PMA‐induced (262 ± 8.43 after 3 h) and *C. albicans* hyphae‐triggered NETs (231.34 ± 16.68 after 3 h). Lacking any recognizable extended threads, candidalysin‐triggered NLS displayed a lower average area per event (151.53 ± 0.62 after 3 h) very much comparable to ionomycin‐triggered NLS (Figs 3C and EV1D).

The post‐translational protein modification (PTM) of histones, in which arginine residues are enzymatically converted into peptidyl citrulline, was analysed since PTM is a driver of chromatin decondensation (Wang *et al*, 2009). The process of the PTM is called deamination or citrullination. Calcium influx activates protein arginine deiminase 4 (PAD4) and the enzyme subsequently facilitates histone citrullination (citH), which contributes to chromatin decondensation and eventually chromatin release. PAD4 activation has been reported for ionomycin (Neeli & Radic, 2013) and nicotine (Hosseinzadeh *et al*, 2016). Thus, we assessed whether candidalysin induced histone citrullination in neutrophils. Indeed, like ionomycin, candidalysin increased histone citrullination in neutrophils above basal levels (Fig 3D). Image quantification of histone citrullination using an antibody directed against citrullinated histone H3 (citH3) demonstrated that citH3 in candidalysin‐stimulated neutrophils appeared more distributed than ionomycin‐stimulated neutrophils, which remained concentrated in compact nuclei (Fig 3E). Notably, we observed ~1.5‐fold increased citH3 levels with ionomycin and candidalysin compared to unstimulated neutrophils. Expectedly, citH3 levels upon PMA stimulation did not increase, but rather decreased which was consistent with previous reports (Konig & Andrade, 2016) (Figs 3D and EV1B). While citrullination levels in unstimulated neutrophils decreased over time, ionomycin stimulation sustained high levels over 5 h. These data strongly suggest that candidalysin induces histone hypercitrullination in neutrophils, which likely promotes chromatin decondensation.

### NADPH oxidase enhances candidalysin‐triggered NLS formation

**Figure 4:**
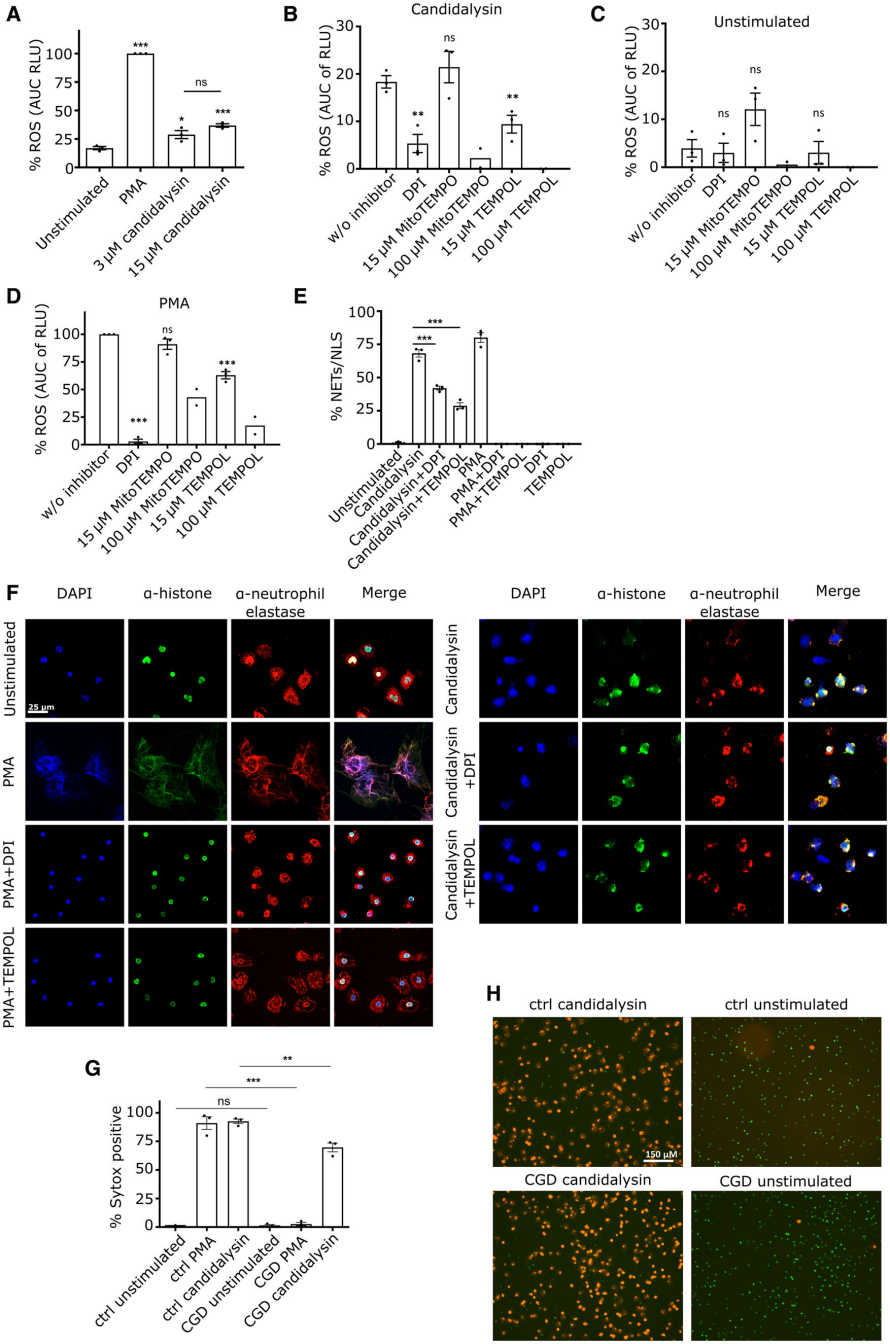
NLS induction by candidalysin is partially ROS dependent AROS response was measured in neutrophils upon stimulation with 100 nM PMA and 15 μM candidalysin using luminol‐based assay (*n* = 3 (4)).B–DNeutrophil ROS response triggered by (B) 15 μM candidalysin, (C) unstimulated or (D) 100 nM PMA in presence of NADPH oxidase inhibitor (DPI), ROS scavenger (TEMPOL) and mitochondrial ROS inhibitor (MitoTEMPO) using luminol‐based assay (*n* = 3 (4), *n* = 2 (4) for 100 μM ROS inhibitors, Tempol and MitoTEMPO).E, FThe impact of stimulus‐triggered neutrophil ROS response on NLS formation was determined after 4.5 h incubation time using (E) quantitative image analysis (*n* = 3 (10–14)) and (F) representative images of confocal immunofluorescence microscopy (60× magnification). Scale bar: 25 μm.GQuantitative image analysis of Sytox‐positive cells after 4 h treatment. Candidalysin and PMA had significantly decreased effects on neutrophils from CGD patients, as compared to neutrophils from healthy donors (*n* = 3 (3)). DNA decondensation was assessed using quantitative image analysis of parallel staining using cell‐impermeable Sytox Orange DNA dye (1 μM) to detect NETs/NLS and cell‐permeable Syto Green DNA dye (250 nM) to determine the total number of cells.HRepresentative images of the analysis in (G) are shown. Scale bar: 150 μm. Data information: Data in (A–D) presented as normalized area under the curve (AUC) over 4 h incubation. Data in (A–E, G) are shown as mean ± SEM and statistically analysed using one‐way ANOVA with Bonferroni *post hoc* test. If not indicated otherwise, statistical significance is indicated in comparison to unstimulated or w/o inhibitor condition. Stars above bars indicate *P < 0.05, **P < 0.01, ***P < 0.001, and “ns” indicates “not significant.” Source data are available online for this figure.

**Figure EV2:**
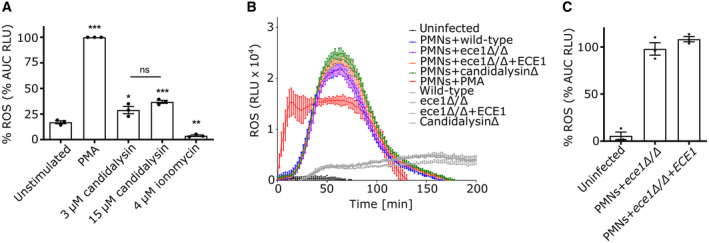
Candidalysin triggers ROS responses in neutrophils but no differences were observed comparing different *C. albicans* strains AA luminol‐based assay was used to quantify ROS production of neutrophils upon PMA, candidalysin and ionomycin stimulation as area under the curve (AUC) over a time period of 4 h (*n* = 3 (4)).B, CInfections of neutrophils with *ece1Δ/Δ C. albicans* strain revealed lower ROS responses in comparison to revertant strain displayed as (B) response over time derived from one representative experiment of three biological replicates with *n* = 4 technical replicates and (C) as AUC over a time period of 3.5 h (*n* = 3 (4)). Data information: Data are shown as mean ± SEM and statistically analysed using one‐way ANOVA with Bonferroni *post hoc* test. If not indicated otherwise, significance is shown in comparison to unstimulated condition. (A, C) are represented as AUC normalized to PMA‐stimulated neutrophil response. Stars above bars indicate *P < 0.05, **P < 0.01, ***P < 0.001, and “ns” indicates “not significant.”

As other peptide toxins can induce NETs independent of NADPH oxidase (Douda *et al*, 2015), we investigated the role of ROS in the induction of NLS by candidalysin using a luminol‐based assay. Treatment of neutrophils with candidalysin induced low levels of ROS, but significantly more than untreated neutrophils (Fig 4A). In the strain context, we observed lower ROS levels in the ece1Δ/Δ strain compared to its revertant strain, however, not to a significant extent (Fig EV2). Next, we assessed whether NADPH oxidase‐dependent ROS or mitochondrial ROS was induced by candidalysin. Notably, candidalysin‐induced ROS production was blocked by diphenyl iodonium (DPI), a specific NADPH oxidase inhibitor, and by Tempol, a ROS scavenger. ROS inhibition was also observed with MitoTempo, a scavenger targeting mitochondrial ROS (Fig 4B). The inhibitors alone had no significant effect on neutrophils (Fig 4C). A similar pattern was observed in response to PMA (Fig 4D). PMA activates protein kinase C (PKC) and the subsequent assembly and activation of NADPH oxidase (Fuchs *et al*, 2007). Thus, we concluded that candidalysin triggered predominantly NADPH oxidase‐mediated ROS and also moderate amounts of mitochondrial ROS. Next, we analysed how inhibition of ROS influenced the release of NLS triggered by candidalysin. Both DPI and Tempol blocked candidalysin‐induced NLS by 40–50%, while PMA‐induced NET production was almost entierly blocked by DPI and Tempol (Fig 4E). The data were confirmed by immunofluorescence where neutrophils were stained for DNA, histone and elastase (Fig 4F). Importantly, using NADPH oxidase‐deficient neutrophils isolated from patients (*n* = 3) with chronic granulomatous disease (CGD), we observed a reduction in candidalysin‐triggered NLS (30–40%) that was comparable to the effect of the ROS inhibitors (Fig 4G and H). Together, the data confirmed that candidalysin induces NLS in part in a NADPH oxidase‐dependent fashion.

**Figure 5:**
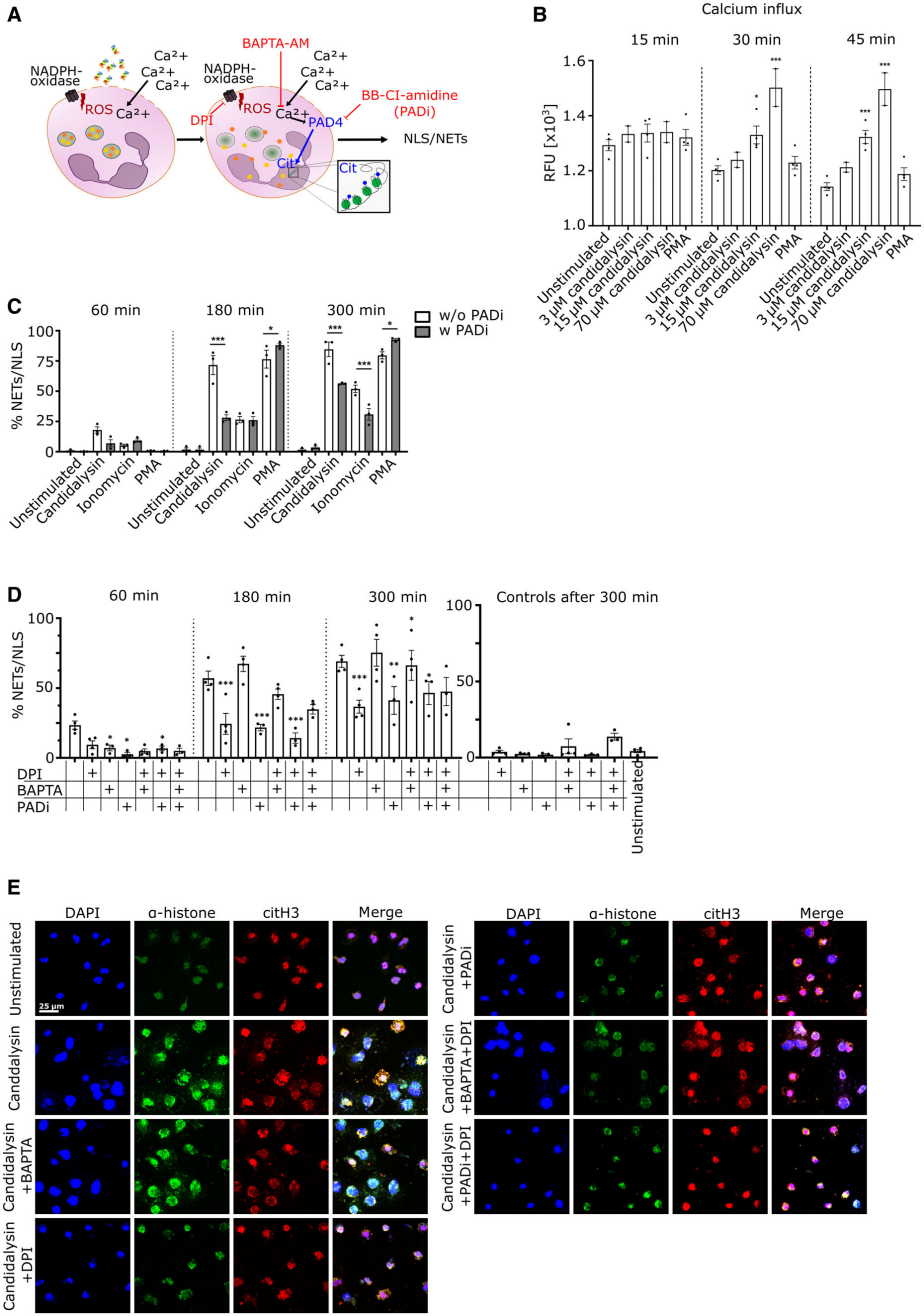
Candidalysin induces NLS via Ca^2+^‐ and ROS‐dependent pathways ASchematic image illustrating the suggested mechanisms by which candidalysin induces NLS in neutrophils. Both downstream effects of ROS and calcium‐dependent PAD4 activation lead to chromatin decondensation. Inhibitors targeting NADPH oxidase (DPI) and PAD activation (BB‐Cl‐amidine, PADi) as well as calcium chelation (BAPTA) show inhibiting effects.B, C(B) Dose‐ and time‐dependent calcium influx in neutrophils through candidalysin was measured with Fluo‐8 AM (*n* = 4 (3)) and (C) image‐based quantification verified PAD‐dependency of NLS formation via ionomycin and candidalysin (*n* = 3 (10–14), data taken from same experiment as Fig 3).DCombination treatment (DPI and PADi) blocking NADPH oxidase‐dependent ROS and PAD activation significantly reduced NLS formation through candidalysin (*n* = 3 or 4 (10–14)).ERepresentative microscopic images (60×) demonstrate decreased morphological alterations through ROS and PAD blockage. Scale bar: 25 μm. Data information: Data in (B–D) are shown as mean ± SEM and statistically analysed using two‐way ANOVA with Bonferroni *post hoc* test. If not indicated otherwise, statistical significance is indicated in comparison to unstimulated or w/o inhibitor condition. Stars above bars indicate *P < 0.05, **P < 0.01, ***P < 0.001. Source data are available online for this figure.

### Calcium influx and PAD4 activity contribute to candidalysin‐triggered NLS

**Figure EV3:**
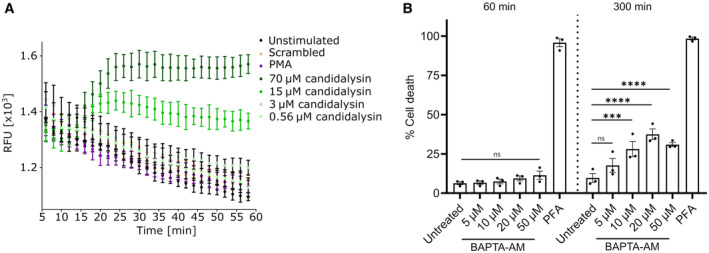
Candidalysin causes Ca^2+^ influx into neutrophils and BAPTA‐AM cytotoxicity increases with incubation time Full dose‐ and time‐dependent calcium influx measurement in neutrophils over 1 h induced by candidalysin. Calcium influx was measured with Fluo‐8 AM and presented as one representative of four biological replicates shown with *n* = 4 technical replicates.Calcium chelator BAPTA‐AM is cytotoxic to neutrophils after 300 min incubation. Cytotoxicity was quantified using propidium iodide staining and flow cytometry analysis. Neutrophils treated with 2% paraformaldehyde for 300 min served as positive control (*n* = 3 (4)). Data information: Data in (A) are shown as mean ± SD and in (B) as mean ± SEM. Statistical analysis was performed using one‐way ANOVA with Bonferroni *post hoc* test. Stars above bars indicate ***P < 0.001 and ****P < 0.0001 and “ns” indicates “not significant.”

Cytoplasmic calcium (Ca^2+^) influx is required to stimulate PAD4 (Neeli *et al*, 2008), which is responsible for histone citrullination and chromatin de‐condensation during ionomycin‐induced hypercitrullination. Since candidalysin also led to increased citrullination of histones in neutrophils, we aimed to elucidate the role of Ca^2+^ during candidalysin neutrophil interaction (Fig 5A). Candidalysin had a clear dose‐dependent effect on intracellular Ca^2+^ influx (Fig 5B). In contrast to Ca^2+^ spikes characteristic for chemokine receptor signalling, candidalysin‐induced Ca^2+^ influx was not instantaneous (Figs 5B and EV3A) but started around 30 min post‐stimulation (Fig 5B). This indicates that candidalysin most probably causes Ca^2+^ influx via pore formation and not via direct receptor stimulation. The PAD inhibitor Cl‐amidine (PADi) reduced candidalysin‐induced NLS formation by 70% after 180 min and by 50% after 300 min, as quantified by microscopic analysis (Fig 5C). Also, the cell‐permeable calcium‐chelator BAPTA‐AM blocked candidalysin‐induced NLS after 60 min (Fig 5D). At later time points, BAPTA‐AM led to an increase in NLS, probably due to toxic effects as indicated by cytotoxicity assay using flow cytometry (Fig EV3B). We thus hypothesized that it may be possible to fully block candidalysin‐induced NLS using a combination of PADi and the NADPH oxidase inhibitor DPI, since this combination would target both the ROS‐dependent and ‐independent axis (Fig 5A). At 180 min, the combination of PADi and DPI abrogated candidalysin‐induced NLS slightly more than the individual inhibitors alone but not beyond the individual inhibitor effect at 300 min. Nevertheless, quantitative image analysis confirmed that PADi and DPI together blocked most NLS formation. For this purpose, neutrophils were stained with antibodies directed against histone H1, citrullinated histone H3 and DNA dye DAPI. The analysis revealed that the treatment with DPI and PADi reduced the amount of patchy areas representing NLS after 300 min to almost background levels (Fig 5E). Taken together, this suggested that candidalysin‐induced NLS formation depended in part on both ROS and PAD4.

**Figure 6:**
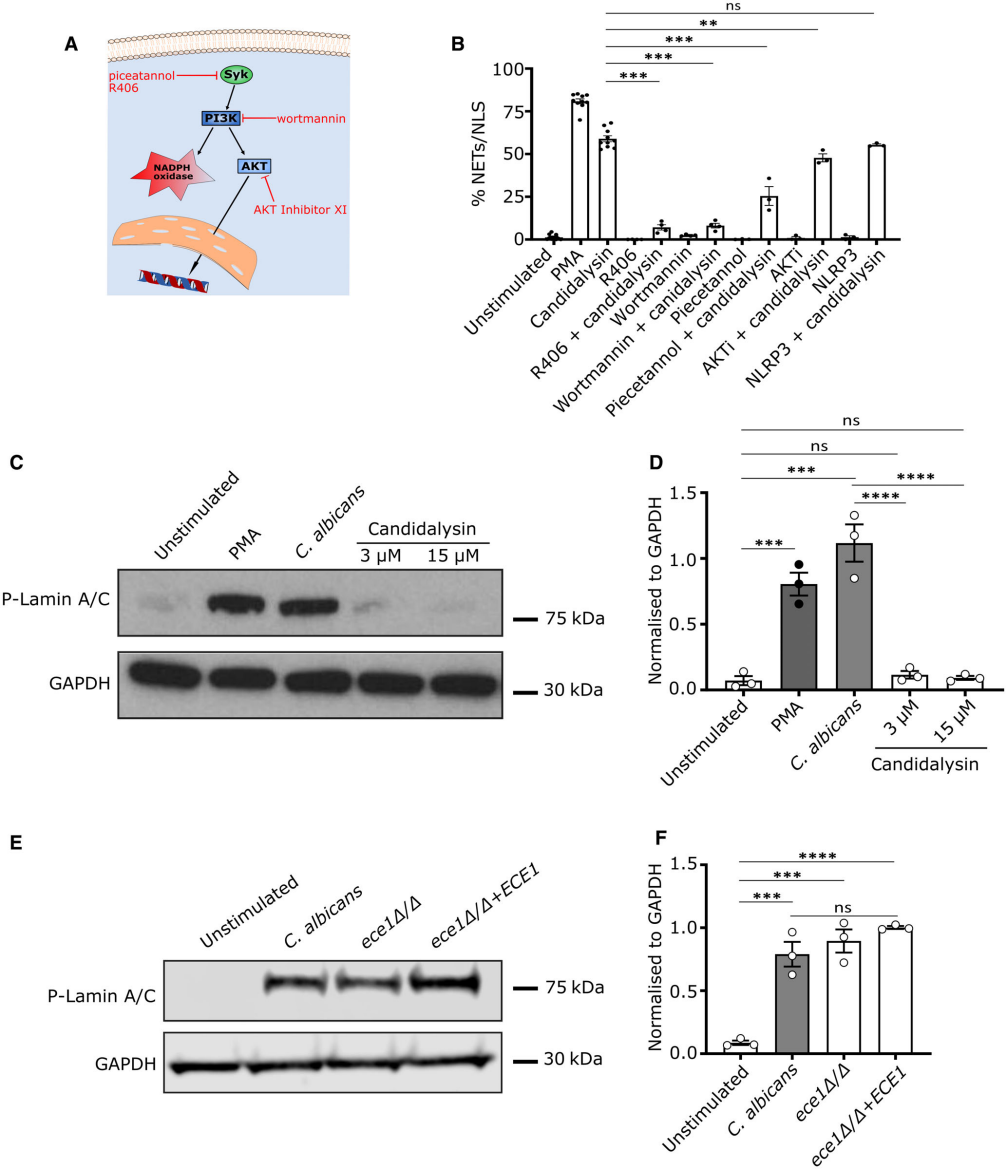
Candidalysin triggers signalling pathways involved in NET formation ASchematic image shows the pathways involved in NET formation and inhibitors used to obtain mechanistic insights.BBlocking main kinases involved in NET formation with 15 μM R406 (SYK), 12.5 μM piceatannol (SYK), 15 μM wortmannin (PI3K), and 2.5 μM AKT inhibitor XI decreased NLS formation upon 4.5 h candidalysin (15 μM) stimulation in human neutrophils from healthy volunteers analysed using image analysis. Pharmacological inhibition of NLRP3 using compound MCC950 (1 μM) did not affect NLS formation (unstimulated/PMA/candidalysin *n* = 10 (10), Wortmannin/R406 (*n* = 4 (10)), and AKTi/NLRP3 (*n* = 3 (10))).C, DWestern blot (C) and quantitative analysis (D) of candidalysin‐stimulated neutrophils did not show phospho‐Lamin A/C activation by candidalysin, in contrast to *C. albicans* or PMA (*n* = 3).E, FSimilarly, western blot (E) and quantitative analysis (F) of *C. albicans*‐stimulated neutrophils comparing wild type, ece1Δ/Δ, and ece1 rev showed no differences in phospho‐Lamin A/C activation (*n* = 3). Data information: Data in (B, D, F) are shown as mean ± SEM, statistically analysed using one‐way ANOVA with Bonferroni *post hoc* test. Stars above bars indicate **P < 0.01, ***P < 0.001, ****P < 0.0001, and “ns” indicates “not significant.” Source data are available online for this figure.

### Candidalysin initiates signalling pathways involved in NET formation

Our data showed that candidalysin induced Ca^2+^ influx in neutrophils, which in turn activates PAD4 (Neeli *et al*, 2008; Gupta *et al*, 2010), leading to chromatin decondensation. Next, we investigated whether additional signalling pathways were involved in candidalysin induction of NLS (Fig 6A). Phosphoinositide‐3 kinase (PI3K) is a signalling molecule upstream of protein kinase B (Akt). PI3K and Akt are known molecular switches for neutrophil apoptosis or NET formation (Douda *et al*, 2014). The spleen tyrosine kinase (SYK), an important signalling protein involved in fungal detection, acts upstream of PI3K (Urban & Backman, 2020). In agreement, SYK signalling contributes to the regulation of NET formation triggered by *C. albicans* (Negoro *et al*, 2020). As *C. albicans* hyphae bind to pathogen recognition receptors (PRRs), activate neutrophils and ultimately promote the release of NETs, we aimed to elucidate whether candidalysin alone could trigger similar pathways in neutrophils via signalling cross‐talk induced by Ca^2+^ influx. Hence, we stimulated neutrophils with candidalysin in the presence or absence of specific inhibitors for SYK, PI3K and Akt (Fig 6B).

Interestingly, SYK blockade with R406 and PI3K blockade with wortmannin reduced NLS formation by candidalysin almost to background levels. The inhibitor piceatannol, which blocks both SYK and PI3K, also blocked NLS formation. In contrast, Akt blockade with AKT inhibitor XI only partially blocked candidalysin‐induced NLS formation. This was expected since Akt signals towards a ROS‐dependent mechanism in neutrophils (Douda *et al*, 2014), which was not critical for NLS induction by candidalysin. In contrast, candidalysin stimulation of neutrophils induced Ca^2+^ influx, which led to PAD4 activation (Fig 5C). Candidalysin has been reported to induce inflammasome activation via NOD‐like receptor family pyrin domain containing 3 (NLRP3) (Kasper *et al*, 2018). However, NLRP3 activation apeared to be dispensable for NLS induction (Fig 6B). Cell cycle molecules are also activated in the later stages of NET formation and a hallmark of cell cycle induction is the phosphorylation of lamin A/C (Amulic *et al*, 2017). However, unlike *C. albicans*, candidalysin did not trigger the phosphorylation of lamin A/C (Fig 6C and D), and *C. albicans* strains expressing or lacking candidalysin induced phosphorylation of lamin A/C to a similar extent (Fig 6E and F). Thus, we concluded that pathways involved in NET formation were triggered by candidalysin via signalling cross‐talk. Notably, these pathways cannot be fully sustained, thus NLS were formed rather than NETs. It is likely that a combination of candidalysin activity and hyphal recognition was required for sustained signalling, which eventually could lead to complete chromatin decondensation and expulsion of NET fibres. This notion was confirmed by lack of downstream activation of the cell cycle proteins by candidalysin (Fig 6C).

### Neutrophils remain functional in the presence of candidalysin

**Figure 7:**
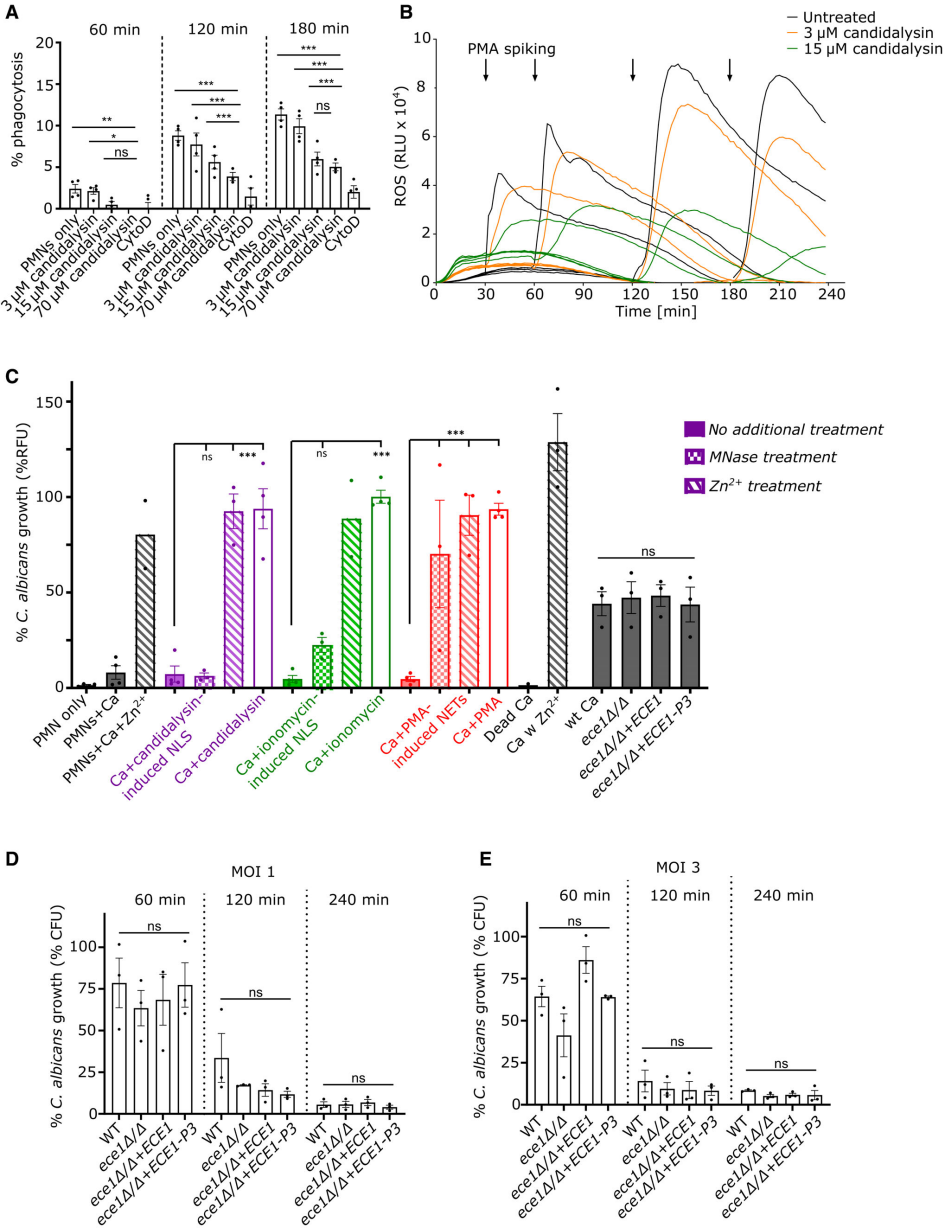
Candidalysin does not abrogate neutrophil functionality and NLS suppress fungal growth ADespite some cytotoxic effects of candidalysin on neutrophils, the cells were still able to phagocytose pre‐opsonized zymosan‐coated beads in presence of candidalysin, with significantly higher levels compared to CytoD‐treated cells (one representative of four biological replicates shown with *n* = 4 technical replicates).BROS production of candidalysin‐treated neutrophils was assessed over time through PMA spiking using a luminol‐based assay (one representative of three biological replicates shown with *n* = 4 technical replicates).CAntimicrobial activity assay resulted in similar fungal growth inhibition via NET‐like structures induced by candidalysin and ionomycin as via canonical NETs induced by PMA (MOI 0.5). *C. albicans* (Ca) growth on pre‐induced NLS/NETs was measured with Calcofluor White staining after 16 h. The addition of Zn^2+^ to candidalysin‐induced NLS before adding *C. albicans* negated the antimicrobial effect in opposite to no response to MNase exposure (*n* = 4 (4)), with following exception: *n* = 3 (4) for MNase and Zn^2+^ treatment and *n* = 2 (4) for Zn^2+^ treatment on ionomycin‐induced NLS and *n* = 2 (4) for intact PMNs with *C. albicans* and Zn^2+^ treatment. In addition, susceptibilities of wild‐type and candidalysin‐deficient *C. albicans* strains to PMA‐induced NETs were compared using slightly more fungal cells (MOI 1.5) for better comparability of the different strains (*n* = 3 (5)).D, ESusceptibility of *C. albicans* wild‐type and candidalysin‐deficient strains towards intact neutrophils was compared by a plating assay (mainly phagocytic killing) at (D) MOI 1 and (E) MOI 3 for 60, 120 and 240 min (*n* = 3 (4)). Data information: (A) Data shown as mean ± SD, (B) data shown as mean and (C–E) data shown as mean ± SEM. Statistical analysis for (A, C–E) was performed using one‐way ANOVA with Bonferroni *post hoc* test. (C–E) In both antimicrobial assays, no statistically significant differences in susceptibilities of the different *C. albicans* strains could be observed. Stars above bars indicate *P < 0.05, **P < 0.01, ***P < 0.001, and “ns” indicates “not significant.” Source data are available online for this figure.

**Figure EV4:**
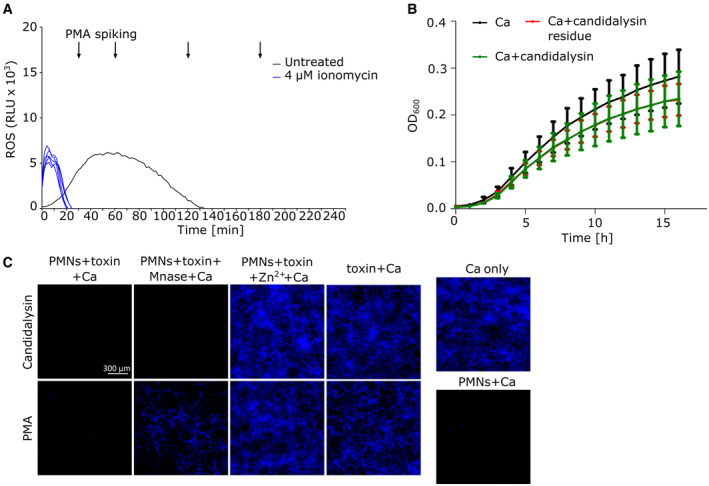
Candidalysin‐induced NLS but not candidalysin affects fungal growth Luminol‐based assay to assess neutrophil ROS response. Ionomycin‐treated neutrophils mounted negligible ROS responses, and during the course of the experiment, the cells became functionally impaired to produce ROS in response to PMA spiking (graph shows one representative of three biological replicates with *n* = 4 technical replicates).OD measurement confirmed that 15 μM of externally added candidalysin did not inhibit the growth of *C. albicans* (Ca). One representative measurement of two biological replicates shown with *n* = 4 technical replicates.Representative microscopic images (10×) taken by Cytation 5 cell imaging reader (BioTek) show that the antimicrobial effect, which was assessed by Calcufluor White staining of fungal cell walls (quantification in Fig 7C), arose from candidalysin‐induced NET‐like structures similarly to the anti‐*Candida* activity of NETs. Scale bar: 300 μm. Data information: Data in (A) are shown as mean and data in (B) are shown as mean ± SD.

Next, we investigated whether neutrophils exposed to candidalysin retain essential antimicrobial functions, such as ROS production and phagocytosis. Although cellular death, as assessed using Sytox Green cell‐impermeable DNA dye, occurs at increasing rates in candidalysin‐treated neutrophils in a dose‐dependent manner (Fig EV1A), neutrophils generally retained their functionality. Neutrophils were able to phagocytose beads in the presence of candidalysin (Fig 7A), which was confirmed by time‐lapse video (Movie EV1), indicating that both Sytox‐negative and Sytox‐positive neutrophils remained functional. Candidalysin‐treated neutrophils were tested for their capacity to mount ROS using PMA as a stimulant. Production of ROS was evident, even 1 or 2 h after candidalysin treatment (Fig 7B). Untreated neutrophils, which were allowed to rest for the times indicated between 30 min and 3 h, showed increased ROS responses upon PMA stimulation (Fig 7B). Notably, even after 2 h treatment with 15 μM candidalysin, neutrophils remained responsive, with ~40–50% of the ROS generated by PMA‐stimulated neutrophils in the absence of candidalysin. The data indicated that the majority of neutrophils did not die immediately upon exposure to 15 μM candidalysin. Finally, ionomycin‐treated cells showed a minor ROS response and were subsequently unable to produce ROS in response to PMA (Fig EV4A).

### Candidalysin‐triggered NLS inhibit *C. albicans* growth but candidalysin expression does not affect anti‐Candida activity of neutrophils

As NETs inhibit the growth of *C. albicans* (Urban *et al*, 2006; Johnson *et al*, 2016), we investigated whether candidalysin‐induced NLS harboured antifungal activity. Thus, we designed an image‐based assay to assess *C. albicans* growth by quantifying Calcofluor White staining in the presence of neutrophils that had been stimulated by candidalysin. Most importantly, NLS triggered by candidalysin showed a strong anti‐*Candida* effect (Fig 7C). Optical density (OD) measurements were used to quantify biomass increase in *C. albicans* corroborating that candidalysin did not suppress *C. albicans* growth (Fig EV4B). Thus, this effect was solely due to candidalysin‐induced NLS (Fig EV4C). In addition, *C. albicans* growth suppression could be reverted by addition of excess Zn^2+^ but not by micrococcal nuclease (MNase) (Fig 7C). This confirmed that, in contrast to canonical NETs, candidalysin‐triggered NLS could not be dismantled and removed by nuclease treatment, probably because NLS are considerably more compact than NETs. Therefore, NLS possessed antimicrobial effects even after nuclease treatment. The anti‐*Candida* effect was most probably exerted via the zinc‐binding protein, calprotectin, as supplementation with excess Zn^2+^ blocked the antimicrobial effect of candidalysin‐triggered NLS (Urban *et al*, 2009).

To corroborate, whether candidalysin deficiency affected *C. albicans*' susceptibility to neutrophil attack, we performed two antimicrobial assays. In the first assay, we determined NET‐mediated anti‐*Candida* activity by preformed NETs comparing wild‐type and candidalysin‐deficient strains. We used the same imaged‐based analysis with Calcofluor White staining (Fig EV4C). To be able to better observe differences in susceptibility of the different strains, we used a slightly higher MOI than for the previous NET inhibition assays which explain higher survival percentage (Fig 7C, black bars on the right side). As expected, candidalysin did not affect the inhibitory effect on *C. albicans* imposed by NETs (Fig 7C). In the second assay, we determined short‐term anti‐*Candida* activity of intact neutrophils, which is predominantly phagocytic elimination, by serial dilution and plating for colony counts. Candidalysin‐deficient and wild‐type strains were killed similarly over the time of 1–4 h, both at MOI 1 and 3 (Fig 7D and E). This indicated that candidalysin expression did not enable evasion from neutrophil phagocytic attack and this result agreed well with our previous finding that wild‐type *C. albicans* engulfed by human neutrophils were unable to escape by hyphal outgrowth (Ermert *et al*, 2013). In conclusion, while candidalysin strongly increased the NET‐inductive capacity of *C. albicans* hyphae, the toxin did neither affect the anti‐*Candida* effect of intact neutrophils nor NETs.

### Candidalysin‐expressing strains induce more NETs and higher citrullination levels than candidalysin‐deficient strains

**Figure 8:**
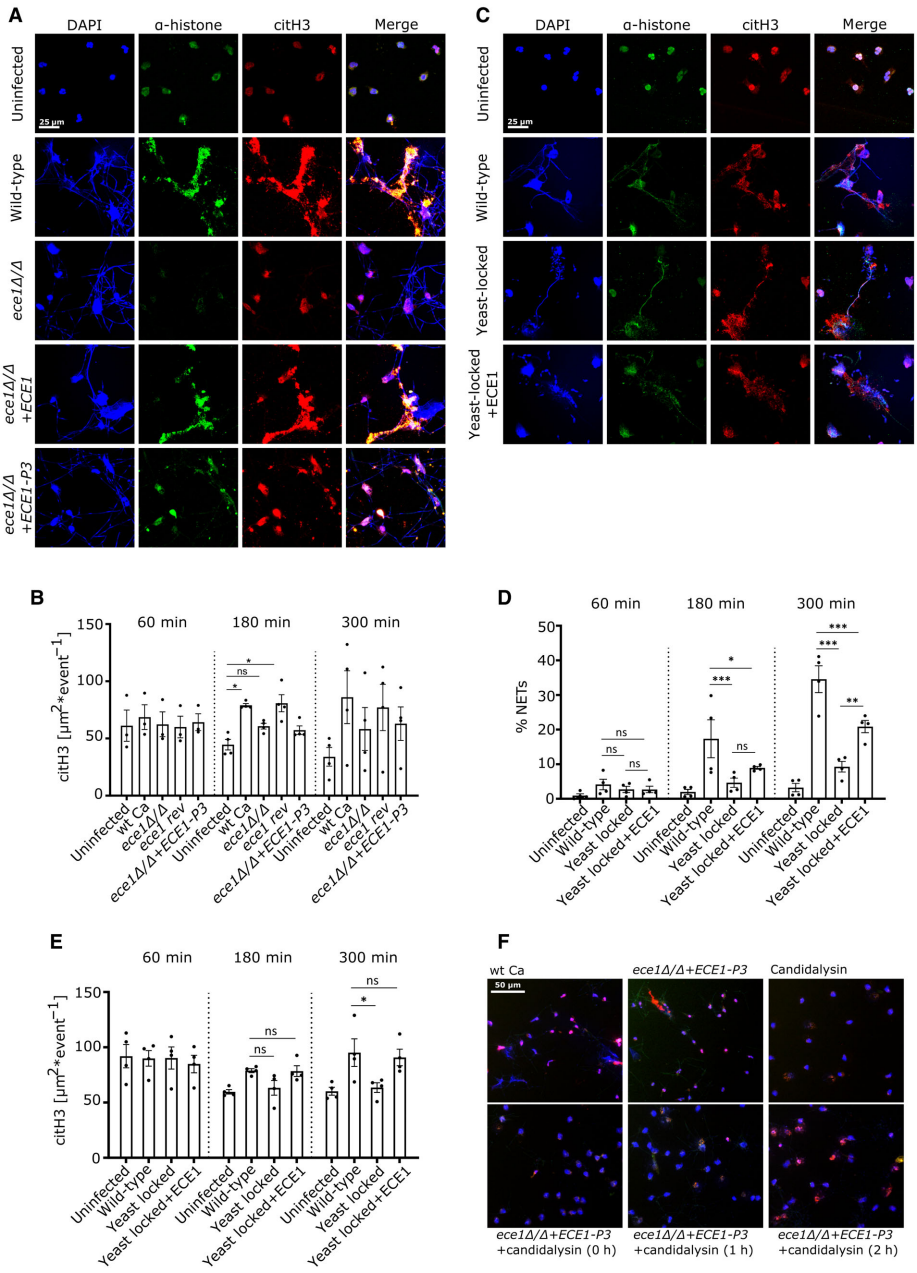
Candidalysin enhances NET formation through histone citrullination ARepresentative immunofluorescence images (60×) of neutrophils infected with *C. albicans* wild‐type and mutant strains (MOI 2) after 3 h identified candidalysin as a major inducer of histone citrullination in human neutrophils. Scale bar: 25 μm.BImage‐based quantification of histine citrullination revealed significantly decreased levels of citH3 in candidalysin‐deficient strains (*n* = 4 (10–14)).CRepresentative immuno‐fluorescence images (40×) of neutrophils infected with *C. albicans* wild‐type and strains (MOI 2) after 3 h revealed that *ECE1* overexpression partially recovered NET‐inductive capacity, despite the fact that the yeast‐locked mutant stimulated fewer NETs. Scale bar: 25 μm.D, E(D) Image‐based quantification (*n* = 4 (10–14)) of NET formation (E) is shown along with histone citrullination.FRepresentative immunofluorescence images (20×) of neutrophils infected with *C. albicans* and 15 μM candidalysin was added 0, 1, or 2 h after the infection. Addition of candidalysin at the different time points after *C. albicans* infection resulted in a shift to NLS structures rather than NETs after 5 h total incubation. Scale bar: 50 μm. Data information: (B, D, E) Data are shown as mean ± SEM and statistically analysed using (B) one‐way ANOVA with Dunnett's multiple‐comparison *post hoc* test and (D, E) one‐way ANOVA with Bonferroni *post hoc* test. Stars above bars indicate *P < 0.05, **P < 0.01, ***P < 0.001 and “ns” indicates “not significant.” (F) One of two experiments is shown. Source data are available online for this figure.

Since candidalysin contributed to the ability of *C. albicans* to induce NETs (Fig 1) and candidalysin alone strongly stimulated histone citrullination, we aimed to establish that citrullination events mainly stem from candidalysin when neutrophils were exposed to candidalysin‐producing and candidalysin‐deficient *C. albicans* strains. To assess this, neutrophils were stained with citrullination‐specific antibodies. Candidalysin‐producing strains induced far more NETs than candidalysin‐deficient strains (Fig 8A). Image‐based quantification corroborated the visual analysis and confirmed that candidalysin‐producing *C. albicans* hyphae promote histone citrullination in neutrophils (Fig 8B). As candidalysin only induced NLS, we concluded that candidalysin augments NET release when the toxin was secreted by *C. albicans* hyphae. We proposed that the combination of candidalysin activity and fungal recognition via pattern recognition receptors (Zawrotniak *et al*, 2017) was required to fully trigger NET formation when neutrophils were exposed to *C. albicans in vivo*.

To test this proposition, neutrophils were infected with a yeast‐locked (*cph1*ΔΔ/*efg1*ΔΔ) strain and an *ECE1*‐overexpressing strain of the same genetic background (*cph1*ΔΔ/*efg1*ΔΔ‐*ECE1*) (Fig 8C). As expected, the yeast‐locked mutant induced significantly fewer NETs than wild‐type *C. albicans* hyphae. Notably, the *ECE1*‐overexpressing yeast‐locked mutant was partly restored in its ability to induce NET release, with twofold increased levels after 5 h compared to the yeast‐locked mutant and over 60% of WT strain (Fig 8D). This confirmed that candidalysin promotes *C. albicans*‐triggered NET release and that this effect was independent of the growth form of *C. albicans*. Elevated citrullination patterns in presence of candidalysin provided further corroboration (Fig 8E). However, *ECE1*‐overexpressing yeast‐locked mutants were delayed in their ability to induce NET release and citrullination, which only emerged after 5 h of stimulation. Finally, we aimed to determine whether candidalysin could rescue NET formation when neutrophils were infected with a candidalysin‐deficient strain (Fig 8F). Interestingly, the addition of candidalysin resulted in a shift to NLS, irrespective of the time of addition, 1 or 2 h post‐infection. The data suggested that candidalysin was the key driver of histone citrullination in neutrophils infected with *C. albicans* and that addition of evenly distributed, external candidalysin in high concentration (15 μM) drove neutrophils towards NLS release despite the presence of *C. albicans* cells. We concluded that, during infection, candidalysin‐triggered Ca^2+^ influx and histone hypercitrullination probably amplified processes in neutrophils, which were induced by *C. albicans* hyphae. These amplified processes culminated in a strongly increased release of NETs which indeed are formidable weapons to control hyphal filaments.

## Discussion

Candidalysin is the first fungal peptide toxin identified in any human fungal pathogen (Moyes *et al*, 2016) and is critical for initiating inflammatory responses that trigger neutrophil recruitment during mucosal and systemic experimental candidiasis (Verma *et al*, 2017; Kasper *et al*, 2018; Richardson *et al*, 2018; Drummond *et al*, 2019; Swidergall *et al*, 2019). As candidalysin is only produced by *C. albicans* hyphae (Wilson *et al*, 2016), we investigated neutrophil responses when these phagocytes encounter candidalysin‐expressing *C. albicans* hyphae or candidalysin alone. Hyphae of candidalysin‐expressing strains induced more NETs than *ECE1*‐deficient and candidalysin‐deficient strains (Fig 1), indicating that candidalysin promotes NET formation. However, incubation of neutrophils with candidalysin was not sufficient to induce NETs (Fig 2). Rather, stimulation with candidalysin led to citrullination of histones via PAD4, leucotoxic hypercitrullination and the release of NLS. In contrast to canonical NETs, NLS were more compact and patchier with fewer clear fibres and threads (Fig 3). The described observations shared key features with NLS induced by the bacterial toxin ionomycin (Konig & Andrade, 2016). Hence, we used ionomycin‐induced NLS and PMA‐induced NETs to better frame our new findings on candidalysin‐induced structures. Notably, candidalysin‐induced NLS did not occur instantaneously. Morphological changes were visible after 30–60 min exposure to candidalysin, and intracellular mixing of granular and nuclear material was observed after ~120 min (Fig 2). After 300 min, ~80% of the neutrophils stimulated with candidalysin released NLS (Fig 3). The role of candidalysin in promoting canonical NET release was confirmed using a yeast‐locked strain overexpressing *ECE1* (Fig 8). While this overexpression construct did not reach the *ECE1* expression levels driven by the endogenous *ECE1* promoter, it nevertheless secreted significant level of candidalysin as described previously (Mogavero *et al*, 2021).

Interestingly, candidalysin induced low activity of NADPH oxidase and consequently ROS production. In CGD patient neutrophils, candidalysin‐induced NLS were significantly reduced compared to control neutrophils; however, 60% of the CGD neutrophils were still able to release NLS (Fig 4). Hence, while NADPH oxidase activity promotes candidalysin‐induced NLS, it is not essential for NLS formation. This notion is clinically supported by the observation that CGD patients very rarely acquire *C. albicans* infections (Marciano *et al*, 2015). In addition to ROS effects, candidalysin also induced the influx of calcium ions into the cytosol of neutrophils. Calcium influx is a known inducer of PAD4, the enzyme responsible for histone citrullination (Neeli & Radic, 2013). We showed that PAD4 was also required for histone decondensation during candidalysin‐induced NLS formation (Fig 5). Calcium influx in neutrophils was unlikely due to candidalysin directly triggering chemokine receptors since calcium influx was slow and over time, and not in a pulse‐like fashion.

High concentrations of candidalysin (70 μM) lysed human neutrophils more rapidly than lower concentrations (15 and 3 μM). Rapid lysis does not allow for regulated cellular processes to be induced within neutrophils. However, at lower concentrations (15 μM), the neutrophils encountering the toxin were still functional (ROS, phagocytosis) and mounted a specific response leading to ROS production, PAD4 activation and the release of NLS. While ionomycin‐ and candidalysin‐induced NLS shared similar key features, such as increased histone citrullination, our study revealed striking differences between the two toxins. In contrast to ionomycin, candidalysin stimulation led to ROS production in neutrophils. This agreed with our finding that signalling pathways involved in NET formation were also triggered by candidalysin (Fig 6). Notably, SYK and PI3K inhibition significantly reduced the amount of candidalysin‐triggered NLS; both signalling molecules are also inducers of NADPH oxidase (Douda *et al*, 2014; Negoro *et al*, 2020). While chelation of calcium ions and PAD4 inhibition reduced NLS formation, cell cycle processes essential for the release of *C. albicans*‐induced canonical NETs, such as the phosphorylation of lamin A/C (Amulic *et al*, 2017), were not activated by candidalysin. This indicated that candidalysin activated NET signalling pathways but these were not sustained or sufficient to induce the release of canonical NETs (Fig 6). This notion was consistent with previous findings describing PAD4 as dispensable for NET formation induced by *C. albicans* hyphae (Guiducci *et al*, 2018).

It is noteworthy that candidalysin‐induced NLS displayed anti‐*Candida* activity. While some reports described NLS as lacking antimicrobial activity (Konig & Andrade, 2016), we clearly observed anti‐*Candida* effects by candidalysin‐triggered NLS. As epithelial cells are able to expunge candidalysin for protection while *C. albicans* hyphae remain adherent (Westman *et al*, 2022), recruited neutrophils may encounter candidalysin before direct contact with hyphae. In addition, neutrophil recruitment is virtually absent in mucosal and systemic models of candidiasis in response to candidalysin‐deficient strains (Verma *et al*, 2017; Richardson *et al*, 2018; Drummond *et al*, 2019; Swidergall *et al*, 2019). Hence, we chose to delineate the capacity of candidalysin‐exposed neutrophils to kill *C. albicans*. Interestingly, candidalysin‐triggered NLS were resistant to nuclease treatment but the resulting anti‐*Candida* effect was Zn^2+^ dependent, indicating that growth inhibition of *C. albicans* by NLS relied on the presence of S100A8/A9 (calprotectin) (Urban *et al*, 2009) and potentially other Zn^2+^‐binding neutrophil proteins. The more compact and thread‐lacking structure of NLS compared to NETs, likely explained why NLS were more resistant to nucleases (Fig 7). During *C. albicans* infection, candidalysin‐induced permeabilization of the plasma membrane could result in large amounts of S100A8/A9 being released and entangling in the structures. Further studies will be required to elucidate the key factors contributing to the anti‐*Candida* effect of candidalysin‐induced NLS. Notably, while candidalysin strongly promoted NET release by *C. albicans* hyphae, the toxin did not increase resistance of *C. albicans* against neither phagocytic killing nor NET‐mediated inhibition (Fig 7C–E). One of the main goals of the study was to delineate contribution of candidalysin to neutrophil responses either as factor released by *C. albicans* hyphae or as singular peptide toxin. Our data demonstrate that candidalysin was the main driver of histone citrullination in neutrophils infected with *C. albicans* (Fig 8). Lack of candidalysin production in *C. albicans* resulted in significantly reduced histone citrullination, accompanied by decreased NET formation. However, citrullination was not required for NET release but rather governs the formation of NLS, which was dominant when candidalysin was added exogenously with even distribution throughout the cell suspension. With regard to *C. albicans* hyphae secreting candidalysin, local concentrations of the toxin are likely to vary to a large degree, particularly when the candidalysin‐secreting hypha is engulfed by a neutrophil. Therefore, it may be difficult to discriminate NLS form NETs during the interaction of neutrophils and *C. albicans*, as both structures may be induced concurrently (Konig & Andrade, 2016). It seems logical that the pore‐forming activity of candidalysin augments the release of NET fibres during *C. albicans* infection, where PRRs will additionally be triggered on neutrophils, resulting in combinatorial activation of downstream pathways. In line with this notion, candidalysin drove histone citrullination, which contributed to chromatin decondensation. On the contrary, when neutrophils were exposed to candidalysin alone, the activation of pathways involved in NET formation was insufficiently sustained, resulting in the emergence of NLS. Importantly, our finding that hyphae induced NETs at the one hand and that candidalysin alone induced NLS on the other hand, provided a possible explanation for why opsonized *C. albicans* induced NETs in a ROS‐dependent fashion, whereas un‐opsonized *C. albicans* induced NETs in an ROS‐independent fashion (Wu *et al*, 2019). As such, it appears that candidalysin had a more dominant effect in experimental settings without serum opsonization and a less dominant effect in the presence of serum opsonization.

In summary, this study shows that candidalysin strongly promoted NET formation during *C. albicans* infection but exclusively NLS formation when present alone. Neutrophils encountering candidalysin‐expressing hyphae were able to adequately respond by releasing increased amounts of NETs, whereas secretion of candidalysin did not allow hyphae to evade neutrophil attack. During *C. albicans* infection, candidalysin drove the release of extracellular chromatin structures by both ROS‐dependent and ROS‐independent pathways, providing a possible rationale for the virtual absence of severe *C. albicans* infection in CGD patients. Importantly, neutrophils remained functional in the presence of candidalysin as both NETs and NLS display anti‐*Candida* activity. Hence. our findings serve as good starting point to further unravel the complexity of NET induction triggered by *C. albicans* and indicate that a combination of candidalysin (this study) and hyphal recognition (Ermert *et al*, 2009; Byrd *et al*, 2013; Branzk *et al*, 2014; Zawrotniak *et al*, 2017) drives NET formation during *C. albicans* infection.

## Materials and Methods

### Fungal strain culture

The *Candida albicans* strains used in this study are listed in Table 1. In all cases, *C. albicans* was incubated in complete dropout medium (SC medium) for 16 h at 30°C. If not otherwise stated, a fresh subculture was inoculated in SC medium for 3 h before finally being washed three times with PBS, counted and adjusted according to each experimental protocol.

**Table 1:** Overview of *C. albicans* strains used in this study.

Fungal strain	Parental strain	Description	Genotype	Reference
*SC5314*		*Candida albicans* standard wild‐type strain		Gillum *et al* (1984)
*BWP17+Clp30*		Wild‐type strain	ura3::λimm434/ura3::λimm434 arg4::hisG/arg4::hisG his1::hisG/his1::hisG + Cip30	Zakikhany *et al* (2007)
*ece1ΔΔ*	*BWP17+CiP30*	*ECE1* knockout	*ece1*::*HIS1*/*ece1*::*ARG4 RPS1*/*rps1*::*URA3*	Moyes *et al* (2016)
*ece1ΔΔ+ECE1*	*BWP17+CiP30*	*ece1Δ* revertant	*ece1*::*HIS1*/*ece1*::*ARG4 RPS1*/*rps1*::(*URA3 ECE1*)	Moyes *et al* (2016)
*ece1ΔΔ+ECE1ΔIII*	*BWP17+CiP30*	*C. albicans BWP17‐Clp30* with candidalysin knockout	*ece1*::*HIS1*/*ece1*::*ARG4 RPS1*/*rps1*::(*URA3 ECE1*Δ_184–279_)	Moyes *et al* (2016)
*cph1ΔΔ/efg1ΔΔ*	*CAI4+CiP10*	*C. albicans* yeast‐locked mutant strain	*cph1*::*hisG*/*cph1*::*hisG efg1*::*hisG*/*efg1*::*hisG*‐*URA3*‐*hisG*	Lo *et al* (1997)
*cph1/efg1 ECE1* overexpression	*CAI4+CiP10*	*C. albicans* yeast‐locked mutant strain with *ECE1* overexpression	cph1/efg1 pENO1_ECE1	Mogavero *et al* (2021) and Westman *et al* (2018)

Fungal strains are described with parental strains and genetic background. Relevant references are included.

### Isolation of human polymorphonuclear neutrophils (PMNs)

Blood sampling for research purposes was conducted in accordance with the principles stated in the Declaration of Helsinki, and with agreement with the blood central of the University Hospital of Umeå. CGD patient blood collection was approved by the Ethical Committee of Charité University Hospital, Berlin, Germany. Venous blood samples were drawn from healthy volunteers and CGD patients in EDTA tubes, and neutrophils were isolated as previously described (Thunstrom Salzer *et al*, 2018). In brief, the neutrophil fraction was obtained using density centrifugation in Histopaque 1119 (Sigma‐Aldrich) to separate granulocytes followed by a discontinuous Percoll (GE Healthcare Life Sciences) gradient to isolate neutrophils. After RBC lysis (RBC lysis buffer, BioLegend), the cells were resuspended in RPMI 1640 media (Lonza, supplemented with 5% HEPES) and counted. Only neutrophils with viability above 90% were selected for further experimentation.

### Neutrophil stimulation

Neutrophils were seeded on glass cover slips coated with 0.001% poly‐L‐lysine (Sigma‐Aldrich) with a concentration of 1 × 10^5^ cells per well if not stated otherwise. PMNs were stimulated with 4 μM ionomycin (free acid, Sigma‐Aldrich), 100 nM phorbol 12‐myristate 13‐acetate (PMA, Sigma‐Aldrich), 15 μM Ece1 peptides including candidalysin (if not otherwise stated) or infected with *C. albicans* yeast (MOI 2) for a defined time period, following fixed using 2% paraformaldehyde (PFA) and stored at 4°C. In the infection experiments, the fungus was added to 1 × 10^4^ PMNs 1 h after the cells were seeded.

For the pathway studies, neutrophils were incubated for 30 min before stimulation with 10 μM BB‐Cl‐amidine (PADi, Cayman Chemicals), 15 μM diphenyleneiodium (DPI, Sigma‐Aldrich), 15 μM 4‐hydroxy‐TEMPO (TEMPOL, Sigma‐Aldrich), 10/20 μM BAPTA‐AM (Abcam), 15 μM SYK inhibitors R406 (InvivoGen) and 12.5 μM piceatannol (InvivoGen), 15 μM PI3K blocker wortmannin (InvivoGen), 2.5 μM AKT inhibitor XI (InvivoGen) or NLRP3 blockage using compound 1 μM MCC950 (InvivoGen).

### Immunostaining, microscopy and quantitative image analysis

For immune staining, the cover slips were washed with PBS, cells permeabilized with 0.5% TritonX‐100 (Sigma‐Aldrich) for 1 min and then blocked at room temperature for 30 min in 3% bovine serum albumin (Sigma‐Aldrich) buffer. Antibodies directed against histone H1 (final 1 μg/ml, Acris, #BM465) and citrullinated histone H3 (citrulline R2+R8+R17, 1 μg/ml, Abcam, ab5103) were applied and incubated for 1 h at 37°C followed by secondary antibodies conjugated with Alexa Fluor dyes 488 and 568 (10 μg/ml, Thermo Fisher). DNA was stained with DAPI (1 μg/ml, Sigma‐Aldrich). Prolong Diamond Antifade Mountant (Invitrogen) was used for mounting. For visualization and quantification, 10–14 images per condition with around 50–150 cells were randomly taken with 20× magnification (Nikon Eclipse 90i fluorescence microscope with NIS Elements software) and the analysis was performed with ImageJ.

For quantification of NETs and NET‐like structures (NLS) (modified accordingly; Hosseinzadeh *et al*, 2012; Hosseinzadeh *et al*, 2016), DAPI‐stained events with an area of over 15 μm^2^ were measured and nuclei exceeding 100 μm^2^ were counted. For quantification of citrullinated histone, the Alexa Fluor 568 total stained area was measured and further normalized as signal per cell based on the event count of the DNA staining. NETs are characterized as web‐like structures with threads spanning over several dozens of micrometres, whereas NLS are more compact, patchy and without longer threads.

For some microscopic analyses, human neutrophils were stained against neutrophil elastase (1 μg/ml, Calbiochem, Cat#481001) and *C. albicans* visualized with anti‐*Candida* antibody (1 μg/ml, ProSci, Cat#35‐645).

Confocal images were taken with Nikon A1R confocal (LSM) controlled by Nikon NIS Elements interface with a Nikon Eclipse Ti‐E inverted microscope using 60× magnification.

To quantify NLS from CGD patient neutrophils in comparison to neutrophils from healthy individuals, cells were seeded in a concentration of 1 × 10^5^ cells per well in 24‐well plates in RPMI medium. Neutrophils were stained using cell‐impermeable Sytox Orange DNA dye (1 μM, Thermo Fisher) to detect NETs and cell‐permeable DNA dye Syto Green (250 nM, Thermo Fisher) to determine the total number of cells. NETs/NLS were imaged at 4 h post‐stimulation using 20× magnification on a EVOS FL Auto Microscope (Thermo Fisher).

### Scanning electron microscopy

Neutrophils were stimulated as described above. After fixation, the cells were washed with PBS and subsequently dehydrated in a series of graded ethanol (70, 80, 90, 95 and 100%). After critical point drying with Leica EM CPD300, the cover slips were coated with a 2 nm platinum layer (Quorum Q150T‐ES Sputter Coater). Representative images were acquired using field‐emission scanning electron microscopy (SEM, Carl Zeiss Merlin) with secondary electron detector at accelerating voltage of 4 kV, probe current of 120 pA and a working distance of 5.1 mm.

### Western blot

2 × 10^6^ neutrophils were stimulated with 100 nM PMA, different opsonized *C. albicans* strains (MOI 5) or candidalysin (3 and 15 μM) for 90 min in tubes, followed by centrifugation at 400 *g* for 5 min. Neutrophils were then resuspended in 40 μl PBS supplemented with 1× protease and phosphatase inhibitor (Thermo Fisher) and placed on ice for 10 min. Subsequently, SDS was added, samples were boiled at 100°C for 10 min, sonicated with 3 pulses of 15 s at 100% power (QSonica) and stored at −20°C. Ten microlitre of sample was loaded in a 4–12% Bis‐Tris pre‐cast gel (Invitrogen). Gel was transferred to a PVDF membrane and blocked in 1% BSA (Fisher) in TBS‐T (0.1% Tween20), followed by protein detection with anti‐phospho‐lamin A/C (1:1,000, Cell Signaling #13448) and anti‐GAPDH (1:1,000, Cell Signaling #2118).

### Cell death and cytotoxicity assays

Neutrophil cell death or the presence of extracellular DNA was quantified using a Sytox Green‐based (Invitrogen) fluorescence assay similar to previous descriptions (Fuchs *et al*, 2007; Ermert *et al*, 2009). To ultimately quantify NETs or NLS, we always used image‐based assays; the cell death assay was only used as complementation. Briefly, cells were seeded in a black 96‐well plate with a concentration of 5 × 10^4^ cells per well. Subsequently, Sytox Green, a membrane‐impermeable DNA dye, was added to a final concentration of 5 μM, before cells were stimulated. The fluorescence signal was measured in a plate‐based fluorescence spectrophotometer (Fluostar Omega, BMG) at 37°C and 5% CO_2_ for 10 h in intervals of 10 min. The percentage of dead cells was calculated using TritonX‐100 permeabilized neutrophils as 100% control.

To determine the possible cytotoxicity of BAPTA‐AM inhibitor on neutrophils, staining with propidium iodide (PI) and analysis on flow cytometer were used. Briefly, freshly isolated 10^6^ neutrophils were resuspended in clear RPMI 1640 medium in Eppendorf tubes, and BAPTA‐AM inhibitor was added to reach the specified concentrations. To create a positive control with dead population, neutrophils were resuspended in PBS with 4% paraformaldehyde. The Eppendorf tubes were then placed in a humidified incubator set to 37°C and 5% CO2 for 1 or 5 h. Following the incubation, samples were centrifuged at 300 *g* for 10 min, cell pellet was resuspended in fresh PBS with 0.5% human serum albumin and 10 μl of PI (250 μg/ml, Thermo Fisher) was added to all the tubes. After 20 min, the samples were analysed on flow cytometer.

### ROS measurement

The induction of ROS was measured by oxidation of luminol and determined in Varioskan Flash reader (Thermo Fisher) at 37°C. 5 × 10^4^ PMNs per well were seeded into black 96‐well plates and incubated in media containing 50 mM luminol (Sigma‐Aldrich), 1.2 U/well HRP (Sigma‐Aldrich) and different inhibitors for 30 min at 37°C and 5% CO_2_. After stimulation or infection with *C. albicans* (MOI 2), the luminescence measurement was started and data were obtained every 2 min. Each experiment was performed in four technical replicates.

For ROS inhibition TEMPOL, MitoTEMPO and DPI (all from Sigma‐Aldrich) were used at a concentration of 15 or 100 mM. For the functional assessment, 100 nM PMA (Sigma‐Aldrich) was added to previously stimulated neutrophils after 30, 60 or 120 min.

### Phagocytosis assay

Neutrophils (5 × 10^4^ cells/well) were seeded into a black 96‐well plate and stimulated with different concentrations of candidalysin. After 30 min incubation time, 25 μg/well opsonized pHrodo Red Zymosan bioparticle conjugates for phagocytosis (Thermo Fisher) were added and the fluorescence intensity of the beads (excitation 560/emission 585 nm) was measured with Fluostar Omega plate reader (BMG). Acidized beads (phthalate buffer(100 mM; pH 4)) and PMNs with the blocked cytoskeleton (12.5 μM cytochalasin D (CytoD)) served as 100 and 0% control, respectively. Each experiment was performed in four technical replicates. Bead opsonization was performed with 60% human serum for 30 min and the control cells were incubated with CytoD for 80 min.

The time‐lapse imaging (Movie EV1) was performed with pHrodo Red *S. aureus* bioparticle conjugates for phagocytosis (Thermo Fisher) as described above in addition to 5 μM Sytox Green (final concentration). The video shows neutrophils 30 min after addition of 15 μM candidalysin.

### Antimicrobial activity assays

The growth inhibition of candidalysin or PMA pre‐treated neutrophils on different *C. albicans* strains was assessed with an end‐point chitin staining with Calcofluor White (Sigma‐Aldrich). Neutrophils (1 × 10^5^ cells/well) were seeded in a poly‐L‐lysine (Sigma‐Aldrich) pre‐coated 96‐well plate and after 30 min incubation time stimulated with 15 μM candidalysin, 4 μM ionomycin or 100 nM PMA for 5 h. After treating designated wells with 10 U/ml MNase, the total well volume was removed and *C. albicans* in a concentration of 5 × 10^4^ cells/well (MOI 0.5 or MOI 1.5) was added. Thimerosal (Sigma‐Aldrich)‐killed *Candida* served as a control. Designated wells were supplemented with 5 μM ZnSO_4_ (Sigma‐Aldrich) as a Zink source. The plate was incubated for 16 h at 37°C and 5% CO_2_, MNase was added to wells previously not treated and subsequently, the cells were fixed with 4% PFA for 20 min at room temperature. After Calcofluor White staining (0.1 mg/ml for 10 min), nine images per well (technic replicate) with 10× magnification were acquired with Cytation 5 Cell Imaging Reader (BioTek), and a cell number representative fluorescence signal was obtained. Each experiment was performed in four technical replicates. To exclude an inhibitory effect of the toxins itself on *C. albicans*, wells were treated in the absence of neutrophils and then infected with the fungus.

To determine instant antimicrobial activity of intact neutrophils (mainly phagocytic killing), we seeded neutrophils (1 × 10^5^ cells/well) in a 24‐well plate and added diluted and washed *C. albicans* cells from fresh overnight SC cultures to reach MOI 1 and 3, respectively. The plates were incubated at 37°C and 5% CO_2_ for indicated time periods. To stop the assay, detergent Triton‐X was added to a final concentration of 1%, and the suspensions in the wells were thoroughly dispersed using a cell scraper and rigorous up‐and‐down pipetting. Serial dilutions of the extracts were then plated on SC‐medium agar plates and incubated at 30°C for 2 days. Colony‐forming units were subsequently counted.

### Growth curve

To study the growth of *C. albicans* in the presence of candidalysin, a measurement of optical density (λ = 600) was performed. Fifteen micrometre candidalysin was added to a poly‐L‐lysine pre‐coated 96‐well plate as described, before being washed and infected with different concentrations of *C. albicans* or directly added to the well together with the fungus. Data were obtained with Fluostar Omega plate reader (BMG) over 16 h with an interval of 1 h at 37°C and 5% CO_2_. Each experiment was performed in four technical replicates.

### Calcium influx

The measurement of calcium influx into cells was adapted from Schaff *et al* (2008). After neutrophil isolation, the cells were resuspended in HBSS without calcium and magnesium (Lonza). Five micrometre Fluo‐8 AM (Abcam) was added to PMNs at 37°C for 90 min. Cells were washed once and resuspended in RPMI 1640. In a total reaction volume of 120 μl, 1 × 10^5^ cells were seeded into a black 96‐well plate and stimulated with 70, 15, 3 and 0.56 μM candidalysin. After 10 min incubation, the fluorescence was measured (Ex490/Em520) for 60 min with Fluostar Omega plate reader (BMG). Each experiment was performed in three technical replicates.

### Statistical analysis

For all calculations and analyses, GraphPad Prism Software 8.0 (GraphPad Software) was used. Bars represent 95% CI and *P*‐value significance is shown as follows: *P < 0.05, **P < 0.01, ***P < 0.001, ****P < 0.0001 and ns = not significant. If not stated otherwise, numbers of biological replicates using independent neutrophil donors in separate experiments are indicated in the figure legends as *n* = biological replicate number (technical replicate number within each individual experiment).

## Author contributions

**Lucas Unger:** Conceptualization; formal analysis; validation; investigation; visualization; methodology; writing – original draft; project administration; writing – review and editing. **Samuel Skoluda:** Validation; investigation; visualization; methodology. **Emelie Backman:** Validation; investigation; visualization; methodology. **Borko Amulic:** Validation; investigation; visualization; methodology; project administration. **Fernando M Ponce‐Garcia:** Validation; investigation; methodology. **Chinelo NC Etiaba:** Validation; investigation; visualization; methodology. **Sujan Yellagunda:** Validation; investigation; visualization; methodology; project administration. **Renate Krüger:** Resources; supervision; project administration. **Horst von Bernuth:** Resources; supervision; project administration. **Johan Bylund:** Supervision; validation; writing – review and editing. **Bernhard Hube:** Conceptualization; resources; supervision; funding acquisition; validation; methodology; writing – review and editing. **Julian R Naglik:** Conceptualization; resources; supervision; funding acquisition; validation; methodology; writing – review and editing. **Constantin F Urban:** Conceptualization; resources; supervision; funding acquisition; validation; investigation; visualization; methodology; writing – original draft; project administration; writing – review and editing.

## Disclosure and competing interests statement

The authors declare that they have no conflict of interest.

## Supporting information



Expanded View Figures PDF

Movie EV1

PDF+

Source Data for Figure 1

Source Data for Figure 2

Source Data for Figure 3

Source Data for Figure 4

Source Data for Figure 5

Source Data for Figure 6

Source Data for Figure 7

Source Data for Figure 8

## Data Availability

All microscopic images shown in this study and the supplemented movie are available in the BioStudies database (BioImage Archive) with the accession code S‐BIAD849 (https://www.ebi.ac.uk/biostudies/bioimages/studies/S‐BIAD849).
