# Supplementary material for: Candida albicans induces neutrophil extracellular traps and leucotoxic hypercitrullination via candidalysin
Source: EMBO Rep. 2023 Oct 5;24(11):e57571. doi: 10.15252/embr.202357571 (PMC10626426; doi:10.15252/embr.202357571)
Supplement: Supplementary file 1 — Expanded View Figures PDF [file EMBR-24-e57571-s008.pdf]

Expanded View Figures

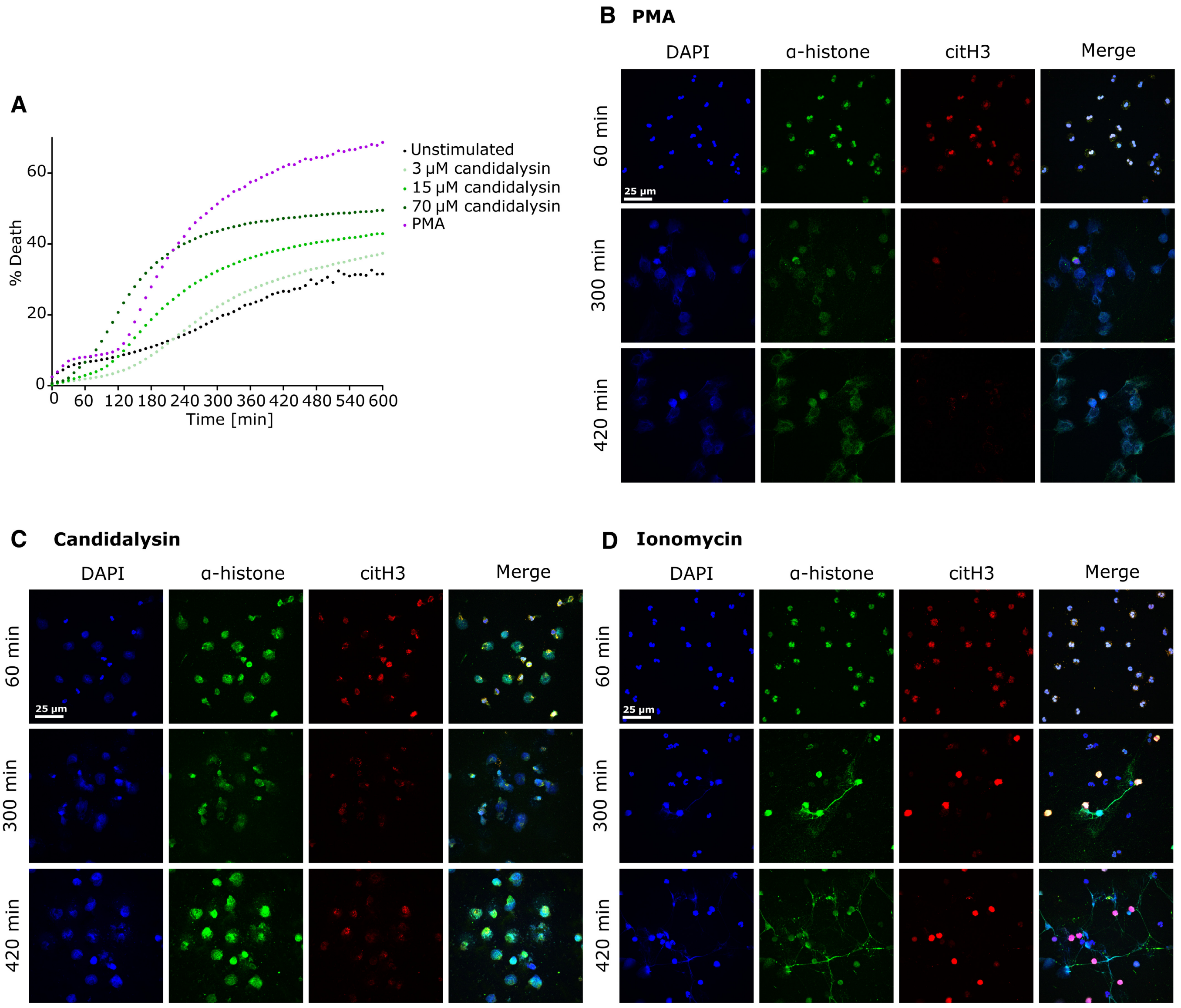

Figure EV1. Candidalysin has dose-dependent effects on human neutrophils.

A Sytox Green staining demonstrated dose- and time-dependent cytotoxic effects of candidalysin on neutrophils (one representative of four biological replicates shown with $n = 4$ technical replicates).

B–D Representative images of confocal immunofluorescence microscopy depict neutrophils that were treated with (B) 100 nM PMA, (C) 15 μM candidalysin and (D) ionomycin at 1, 3, 5 and 7 h incubation time. Progressing chromatin expansion and release of DNA patches or fibres can be observed over time.

Data information: (A) Data presented as mean of percentage of a Triton-X-treated lysis control. (B–D) Visualization accomplished by staining DNA with DAPI (blue channel), primary antibody directed against histones (green channel), and citrullinated histone 3 (citH3). Images taken with Nikon A1R confocal microscope (LSM) controlled by Nikon NIS elements interface with a Nikon Eclipse Ti-E inverted microscope using 60× magnification. If not stated otherwise, numbers of biological replicates using independent neutrophil donors in separate experiments are indicated in the figure legends – n = biological replicate number (technical replicate number within each individual experiment).

 © 2023 The Authors

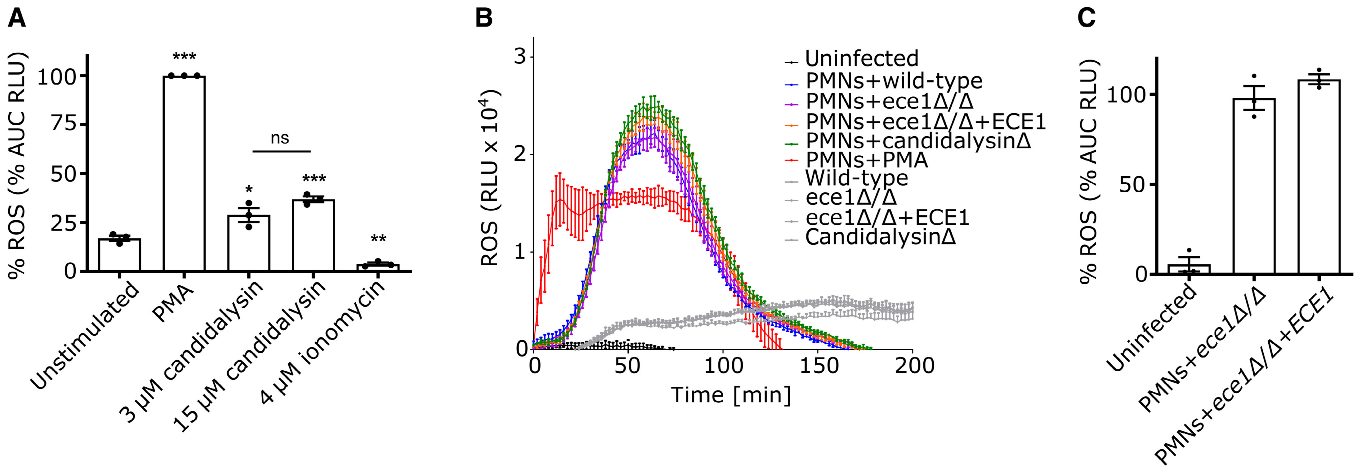

Figure EV2. Candidalysin triggers ROS responses in neutrophils but no differences were observed comparing different *C. albicans strains.*

A A luminol-based assay was used to quantify ROS production of neutrophils upon PMA, candidalysin and ionomycin stimulation as area under the curve (AUC) over a time period of 4 h (n = 3 (4)).

B, C Infections of neutrophils with *ece1Δ/Δ C. albicans* strain revealed lower ROS responses in comparison to revertant strain displayed as (B) response over time derived from one representative experiment of three biological replicates with n = 4 technical replicates and (C) as AUC over a time period of 3.5 h (n = 3 (4)).

Data information: Data are shown as mean ± SEM and statistically analysed using one-way ANOVA with Bonferroni *post hoc* test. If not indicated otherwise, significance is shown in comparison to unstimulated condition. (A, C) are represented as AUC normalized to PMA-stimulated neutrophil response. Stars above bars indicate *P < 0.05, **P < 0.01, ***P < 0.001, and "ns" indicates "not significant."

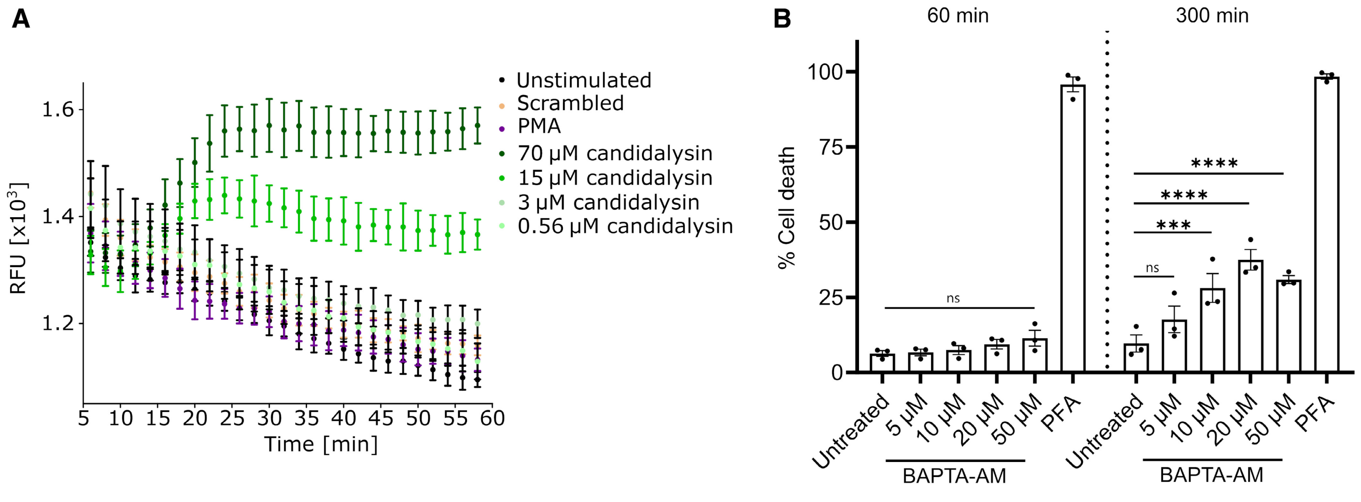

Figure EV3. Candidalysin causes Ca^{2+} influx into neutrophils and BAPTA-AM cytotoxicity increases with incubation time.

A Full dose- and time-dependent calcium influx measurement in neutrophils over 1 h induced by candidalysin. Calcium influx was measured with Fluo-8 AM and presented as one representative of four biological replicates shown with n = 4 technical replicates.

B Calcium chelator BAPTA-AM is cytotoxic to neutrophils after 300 min incubation. Cytotoxicity was quantified using propidium iodide staining and flow cytometry analysis. Neutrophils treated with 2% paraformaldehyde for 300 min served as positive control (n = 3 (4)).

Data information: Data in (A) are shown as mean ± SD and in (B) as mean ± SEM. Statistical analysis was performed using one-way ANOVA with Bonferroni *post hoc* test. Stars above bars indicate ***P < 0.001 and ****P < 0.0001 and "ns" indicates "not significant."

© 2023 The Authors

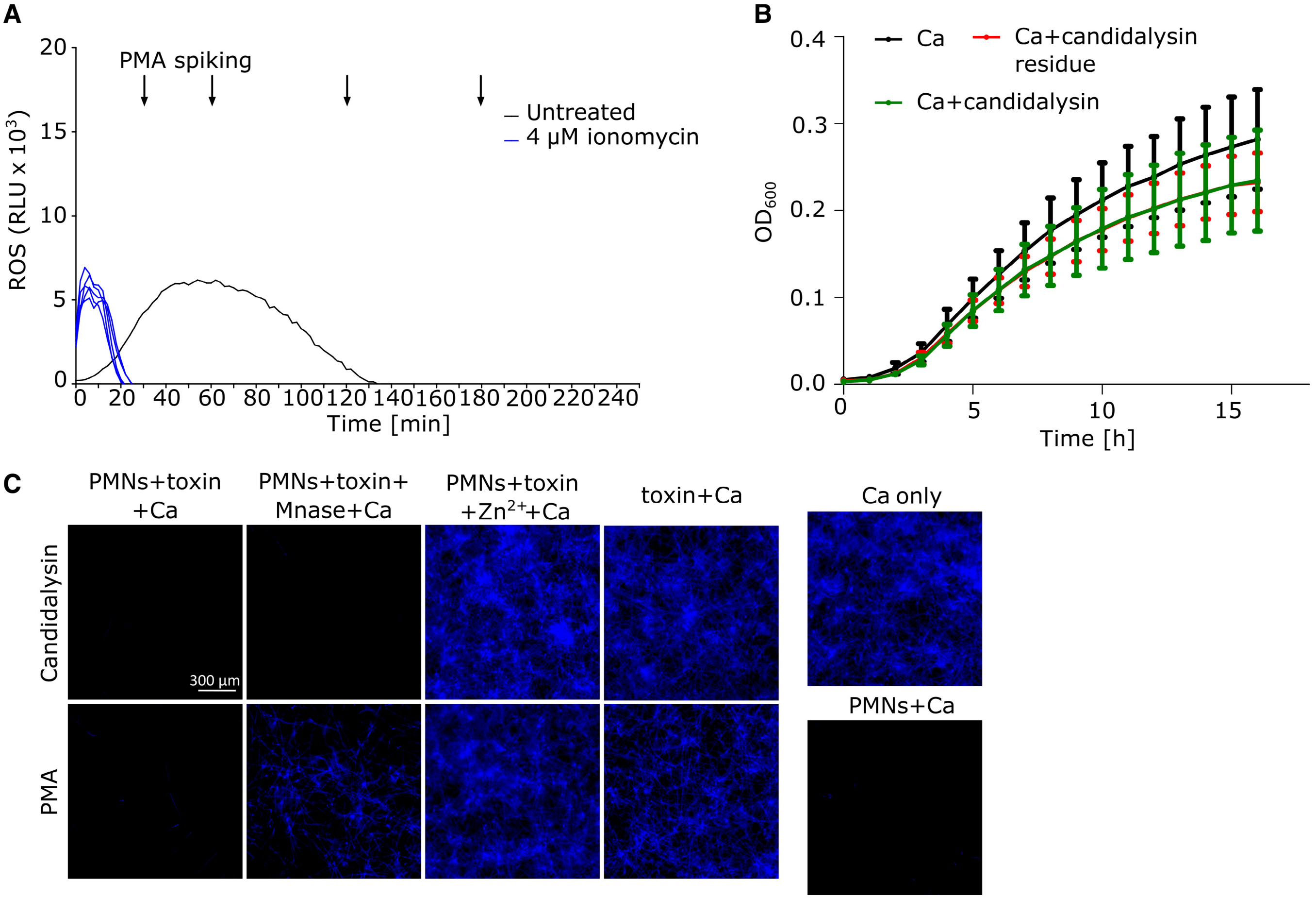

Figure EV4. Candidalysin-induced NLS but not candidalysin affects fungal growth.

A Luminol-based assay to assess neutrophil ROS response. Ionomycin-treated neutrophils mounted negligible ROS responses, and during the course of the experiment, the cells became functionally impaired to produce ROS in response to PMA spiking (graph shows one representative of three biological replicates with $n = 4$ technical replicates).

B OD measurement confirmed that 15 µM of externally added candidalysin did not inhibit the growth of *C. albicans* (Ca). One representative measurement of two biological replicates shown with $n = 4$ technical replicates.

C Representative microscopic images (10×) taken by Cytation 5 cell imaging reader (BioTek) show that the antimicrobial effect, which was assessed by Calcufluor White staining of fungal cell walls (quantification in Fig 7C), arose from candidalysin-induced NET-like structures similarly to the anti-*Candida* activity of NETs. Scale bar: 300 µm.

Data information: Data in (A) are shown as mean and data in (B) are shown as mean ± SD.

 © 2023 The Authors
