# Supplementary figures and images for: Candida albicans induces neutrophil extracellular traps and leucotoxic hypercitrullination via candidalysin
Source: EMBO Rep. 2023 Oct 5;24(11):e57571. doi: 10.15252/embr.202357571 (PMC10626426; doi:10.15252/embr.202357571)

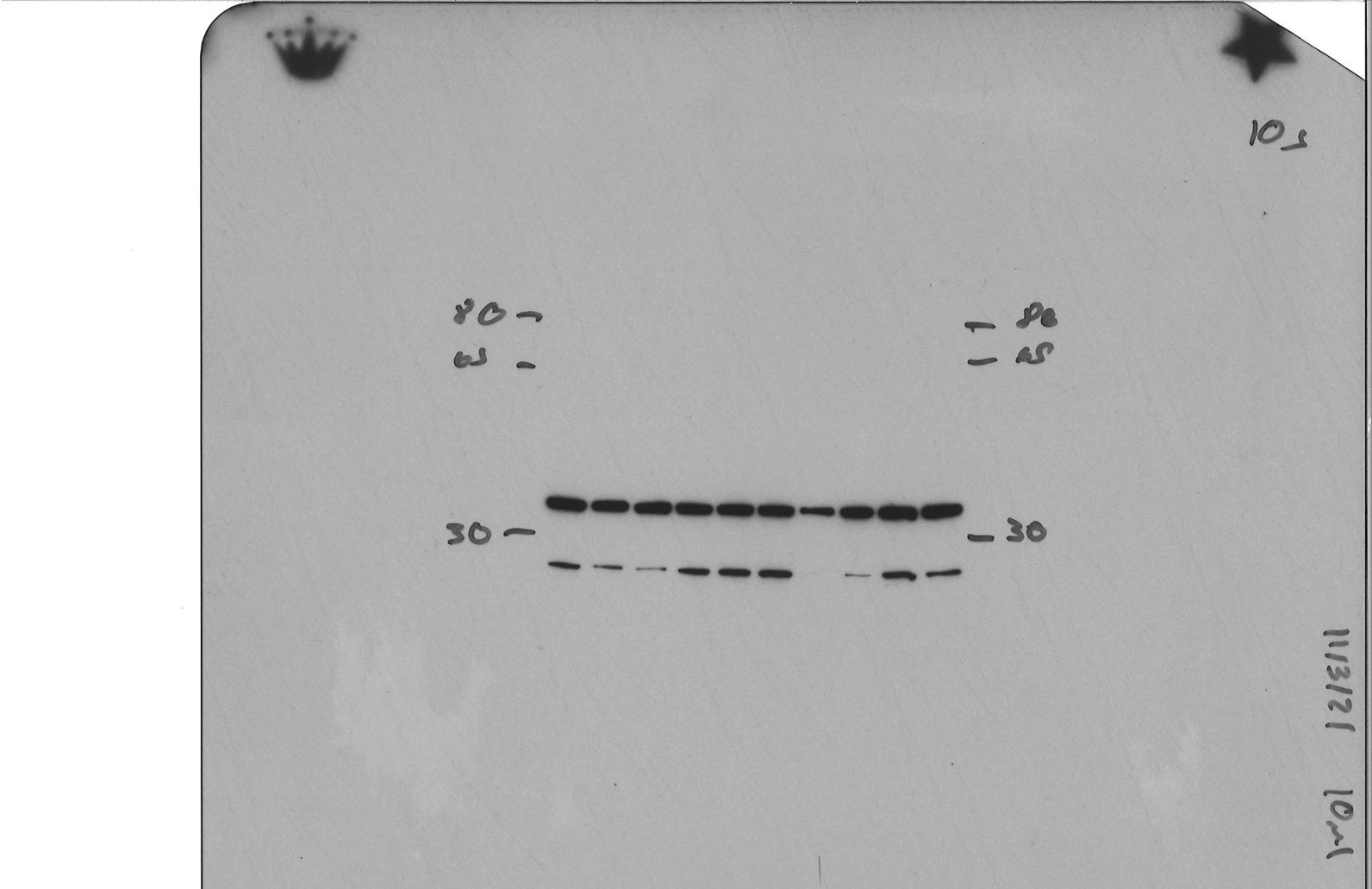

Supplement: Supplementary file 9 — Source Data for Figure 6 [file EMBR-24-e57571-s001.zip › figure 6/6c/P-lamin-N1/GAPDH-N1.jpg]

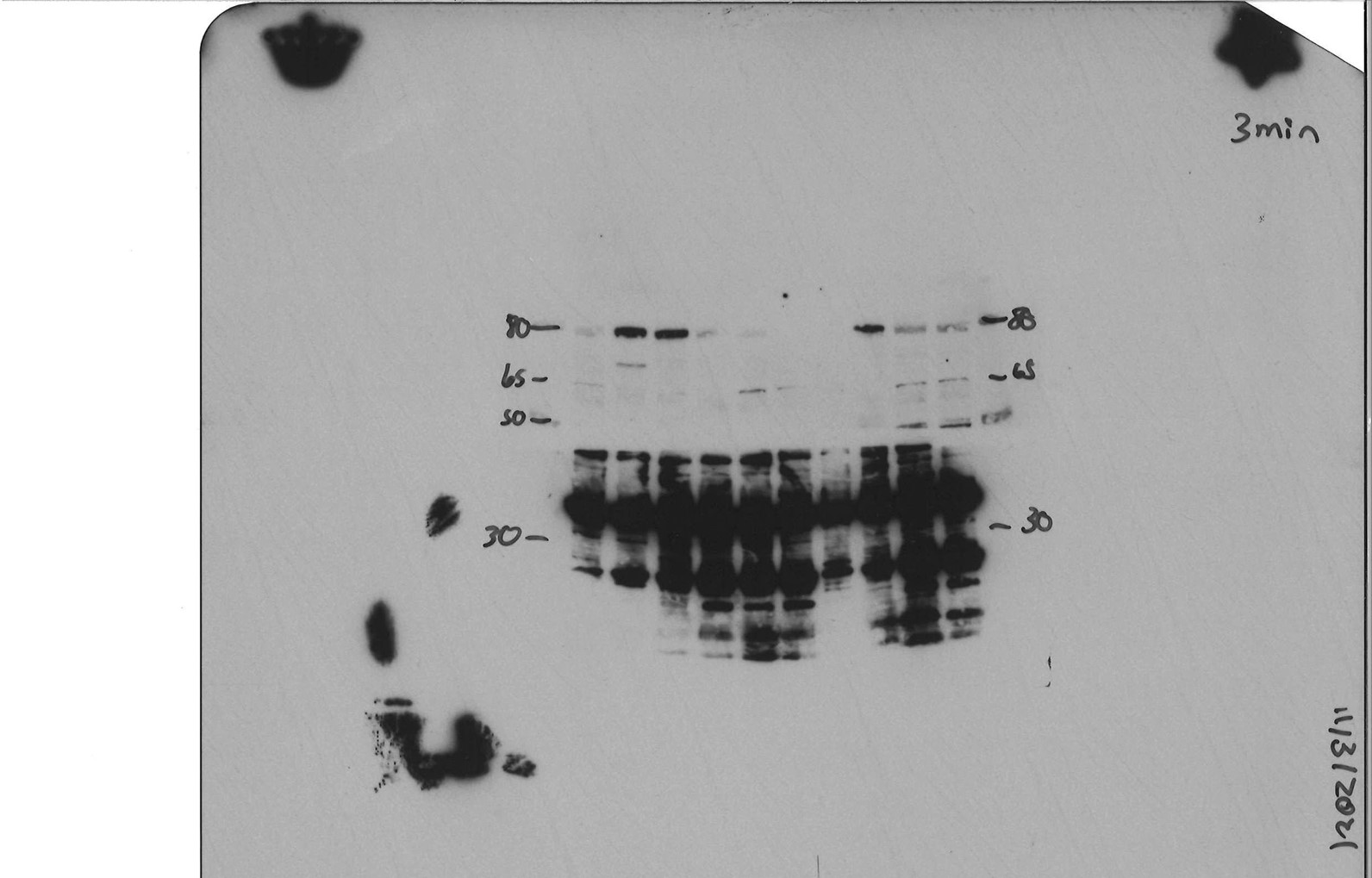

Supplement: Supplementary file 9 — Source Data for Figure 6 [file EMBR-24-e57571-s001.zip › figure 6/6c/P-lamin-N1/P-lamin-N1.jpg]

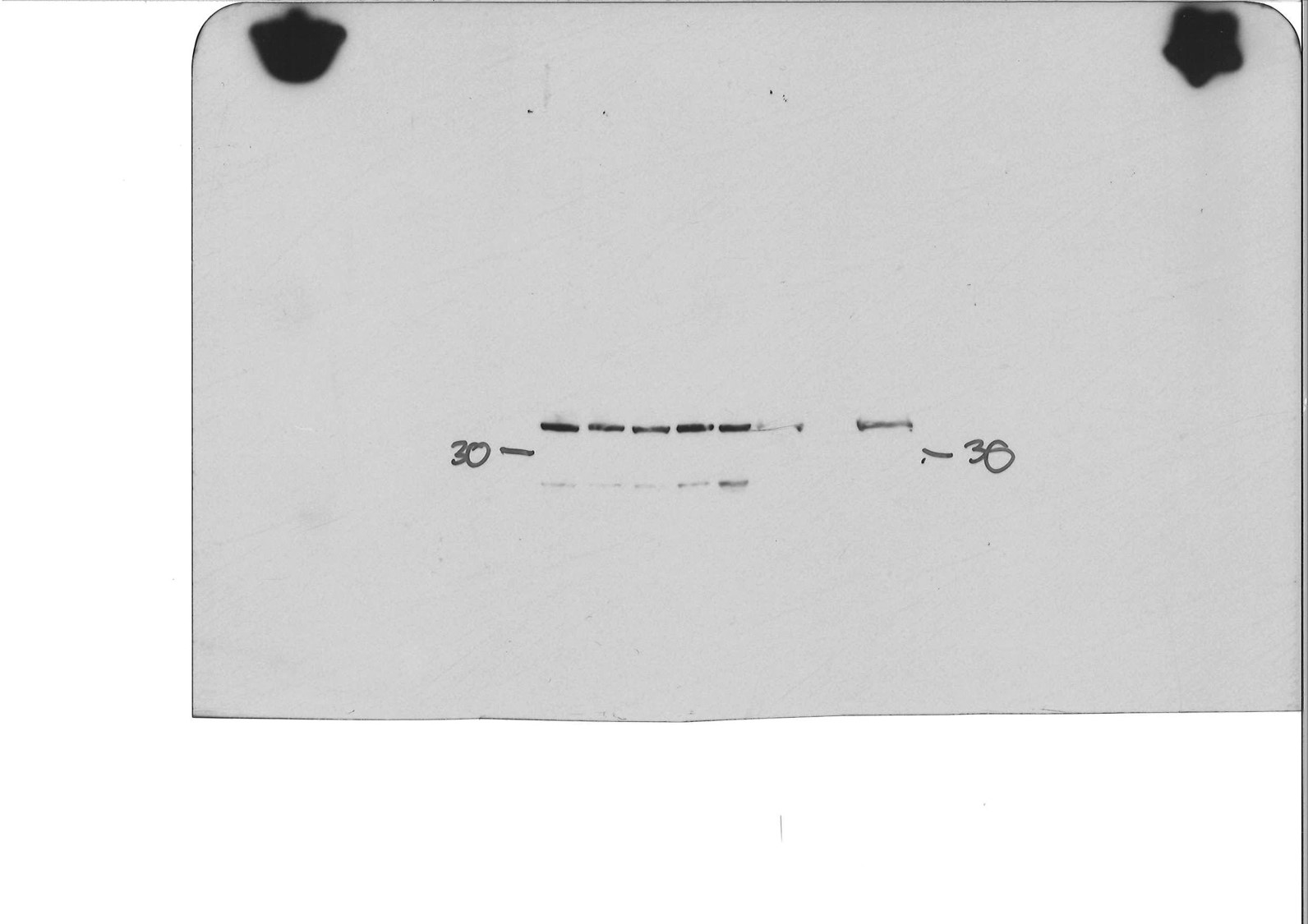

Supplement: Supplementary file 9 — Source Data for Figure 6 [file EMBR-24-e57571-s001.zip › figure 6/6c/P-lamins-N2/GAPDH-N2.jpg]

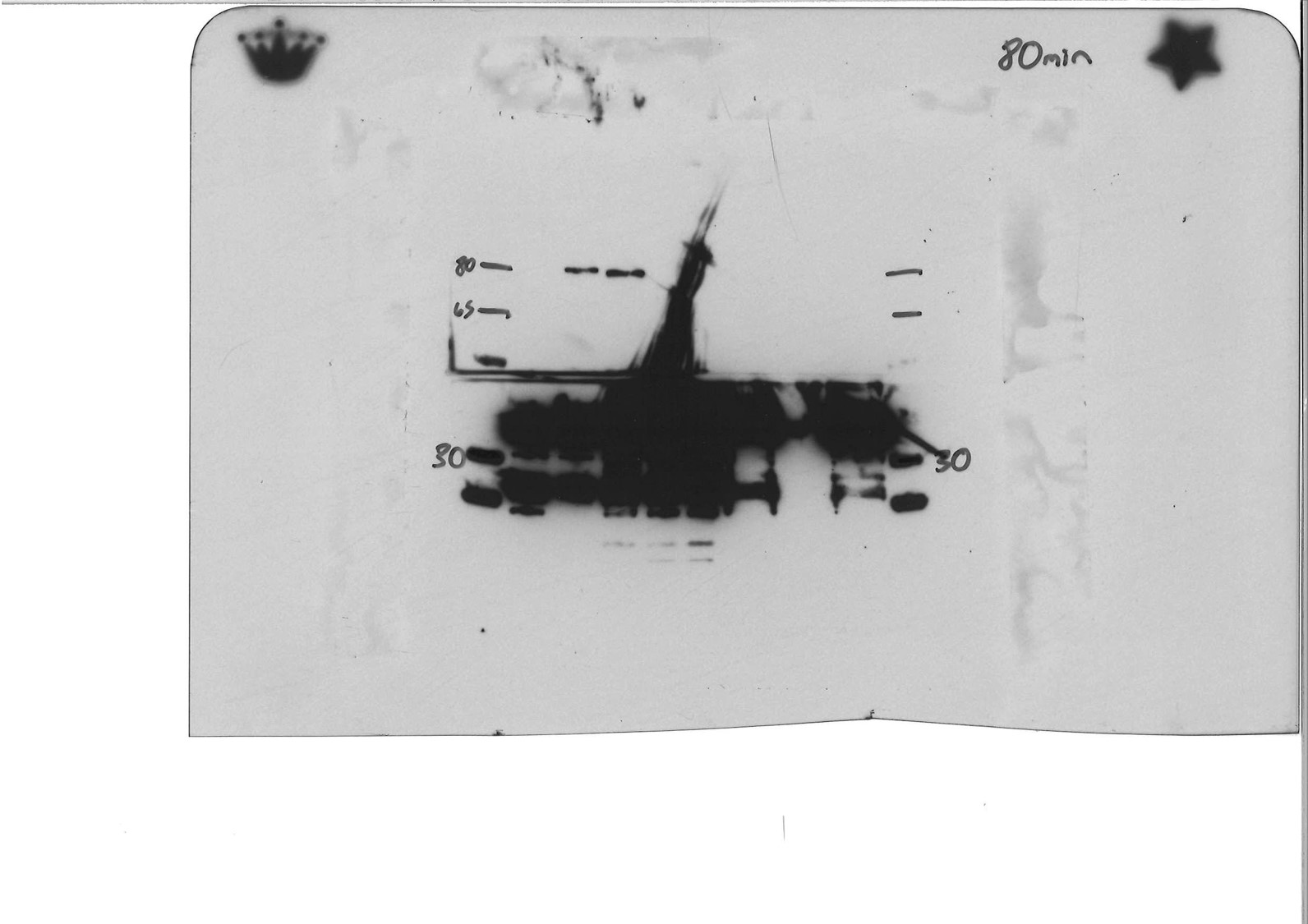

Supplement: Supplementary file 9 — Source Data for Figure 6 [file EMBR-24-e57571-s001.zip › figure 6/6c/P-lamins-N2/P-Lamin-N2.jpg]

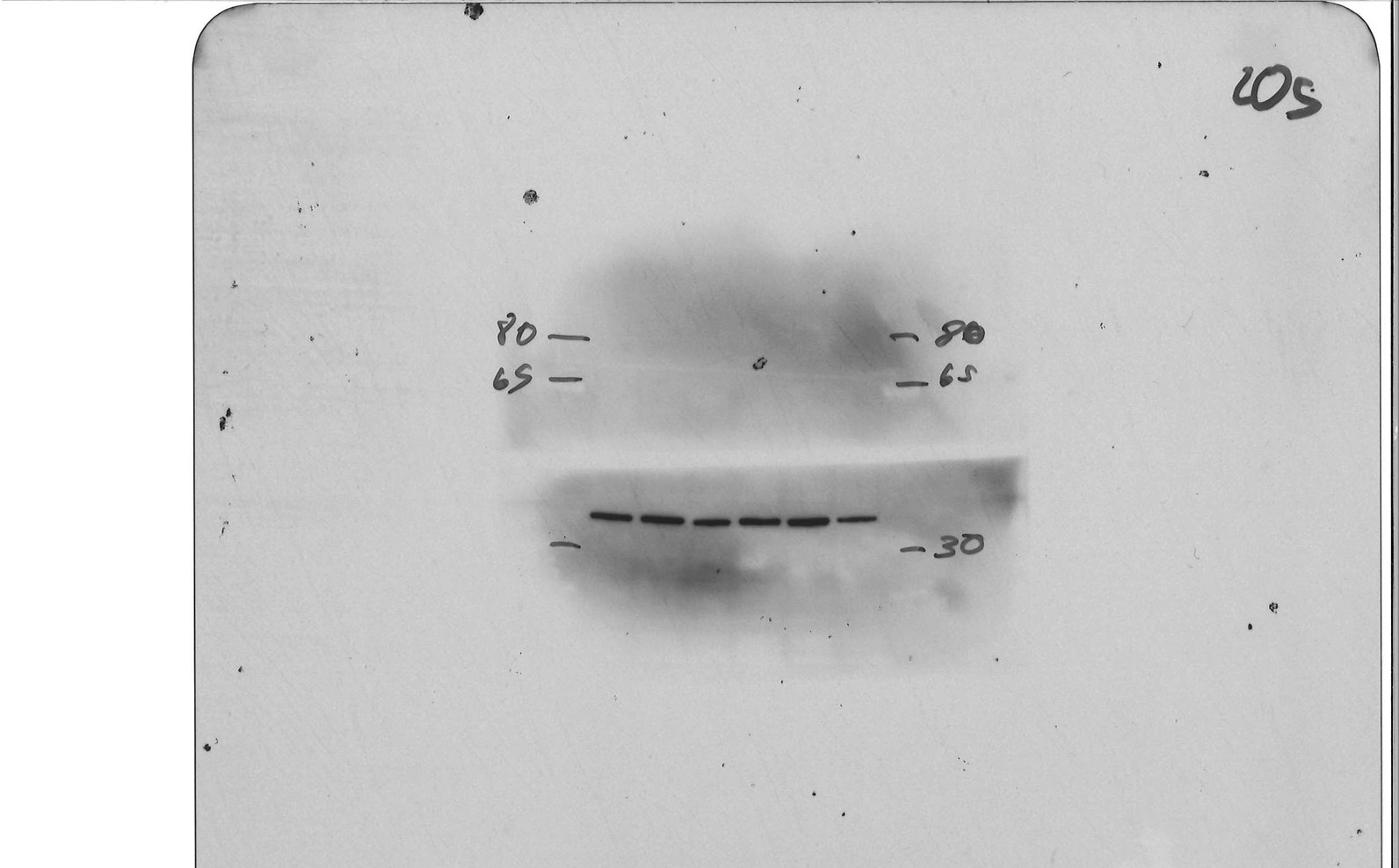

Supplement: Supplementary file 9 — Source Data for Figure 6 [file EMBR-24-e57571-s001.zip › figure 6/6c/P-lamins-N3/GAPDH-N3.jpg]

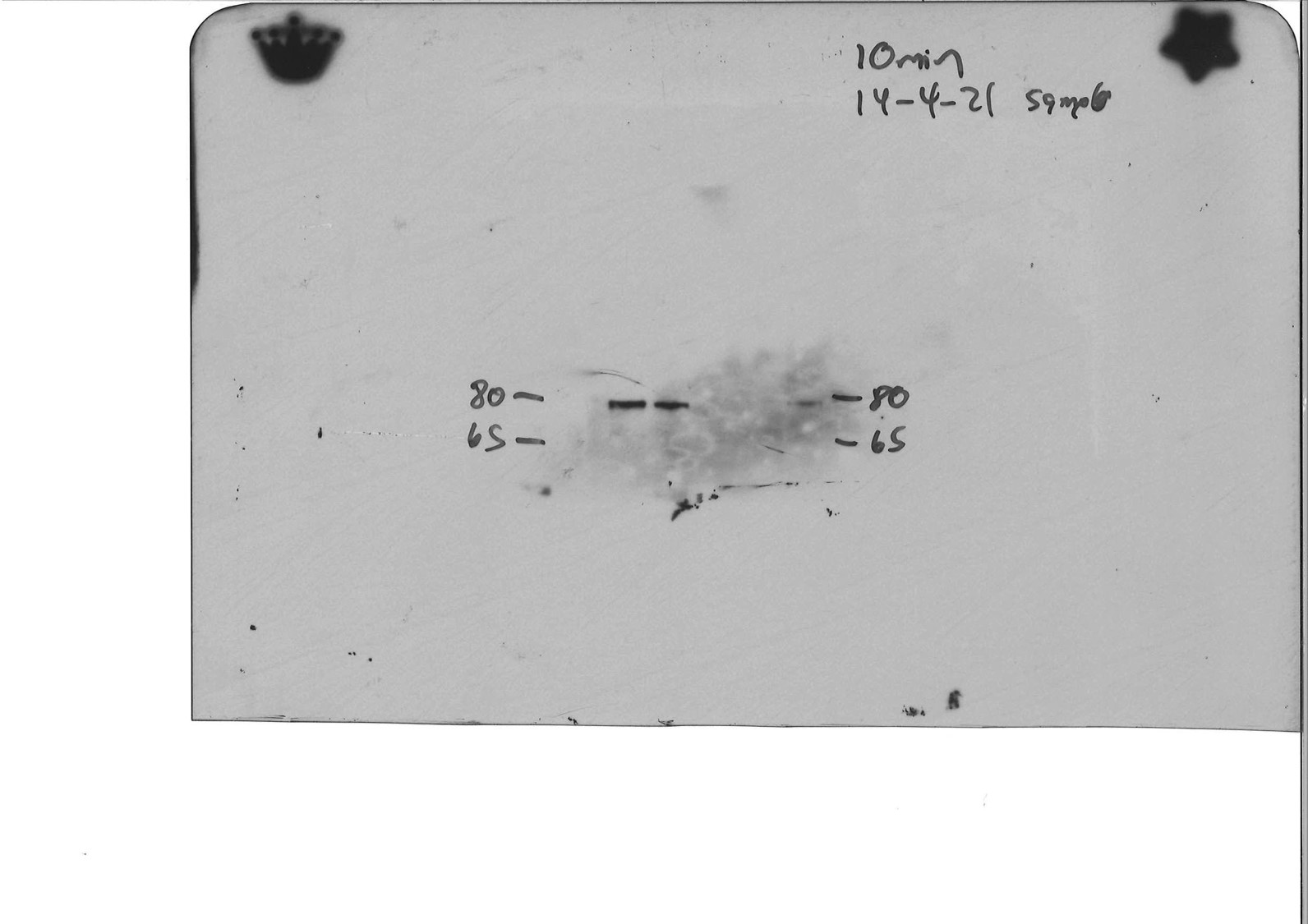

Supplement: Supplementary file 9 — Source Data for Figure 6 [file EMBR-24-e57571-s001.zip › figure 6/6c/P-lamins-N3/P-lamin-N3.jpg]

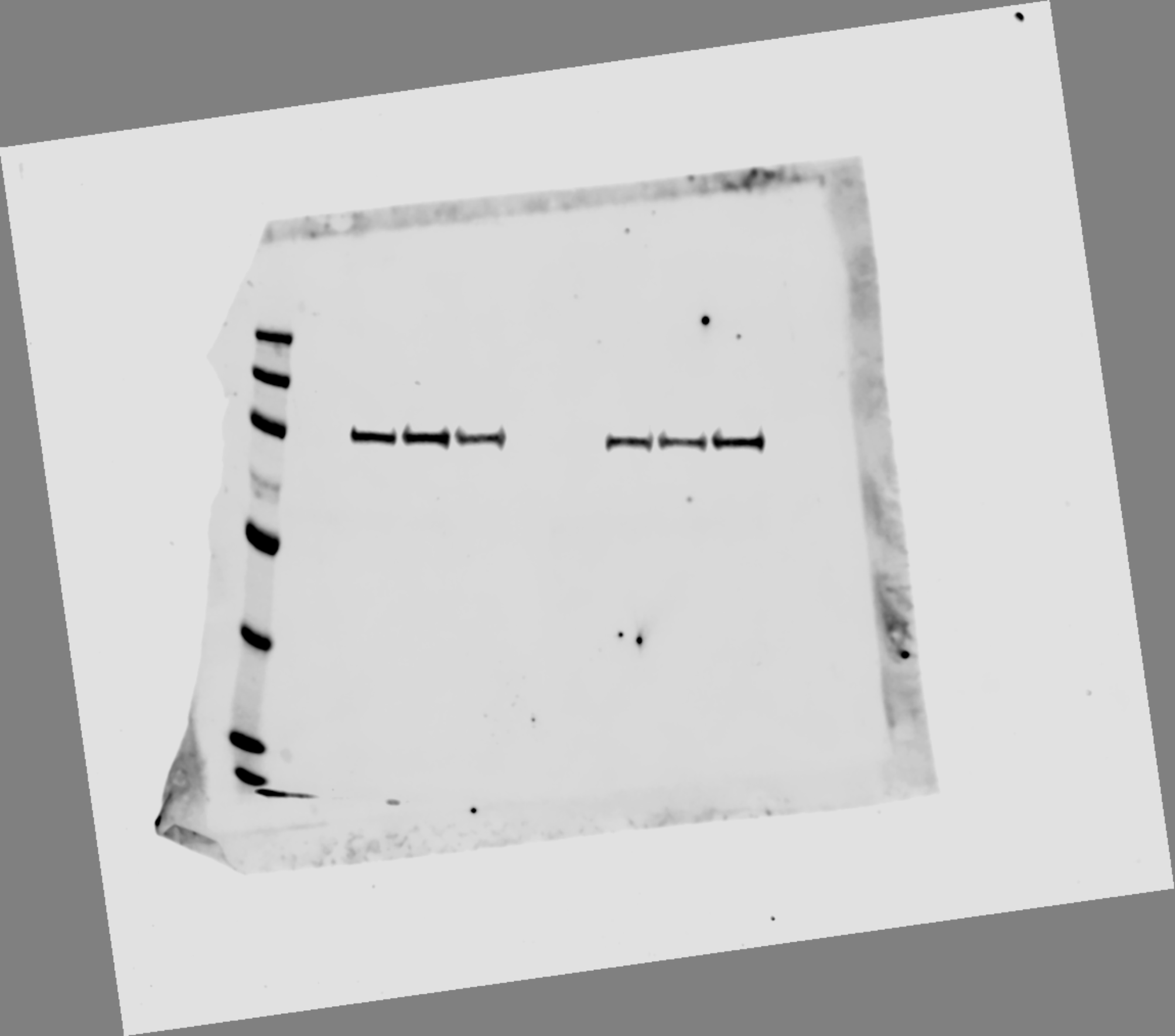

Supplement: Supplementary file 9 — Source Data for Figure 6 [file EMBR-24-e57571-s001.zip › figure 6/6e/original/CANDID~1.TIF]

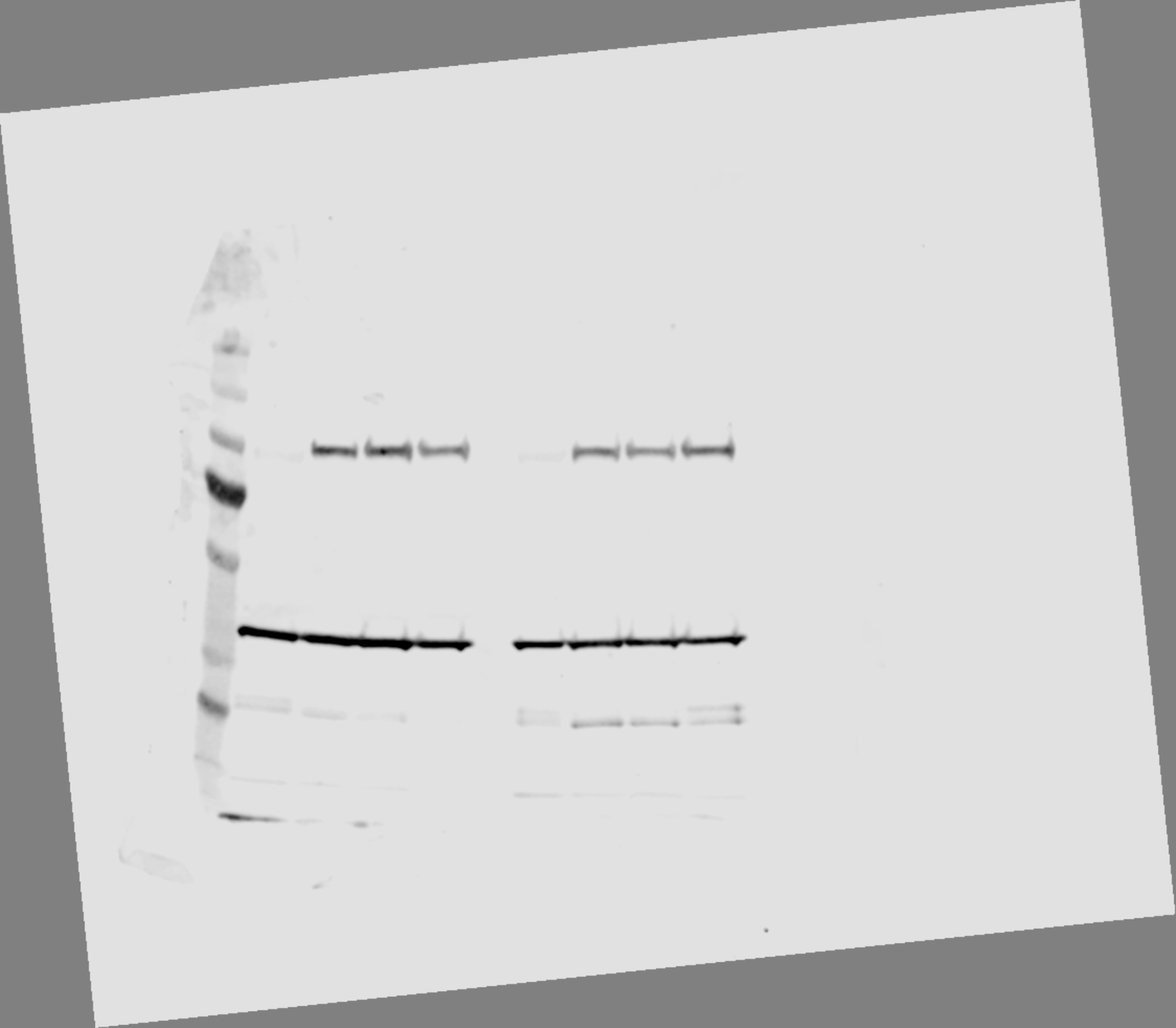

Supplement: Supplementary file 9 — Source Data for Figure 6 [file EMBR-24-e57571-s001.zip › figure 6/6e/original/CANDID~2.TIF]

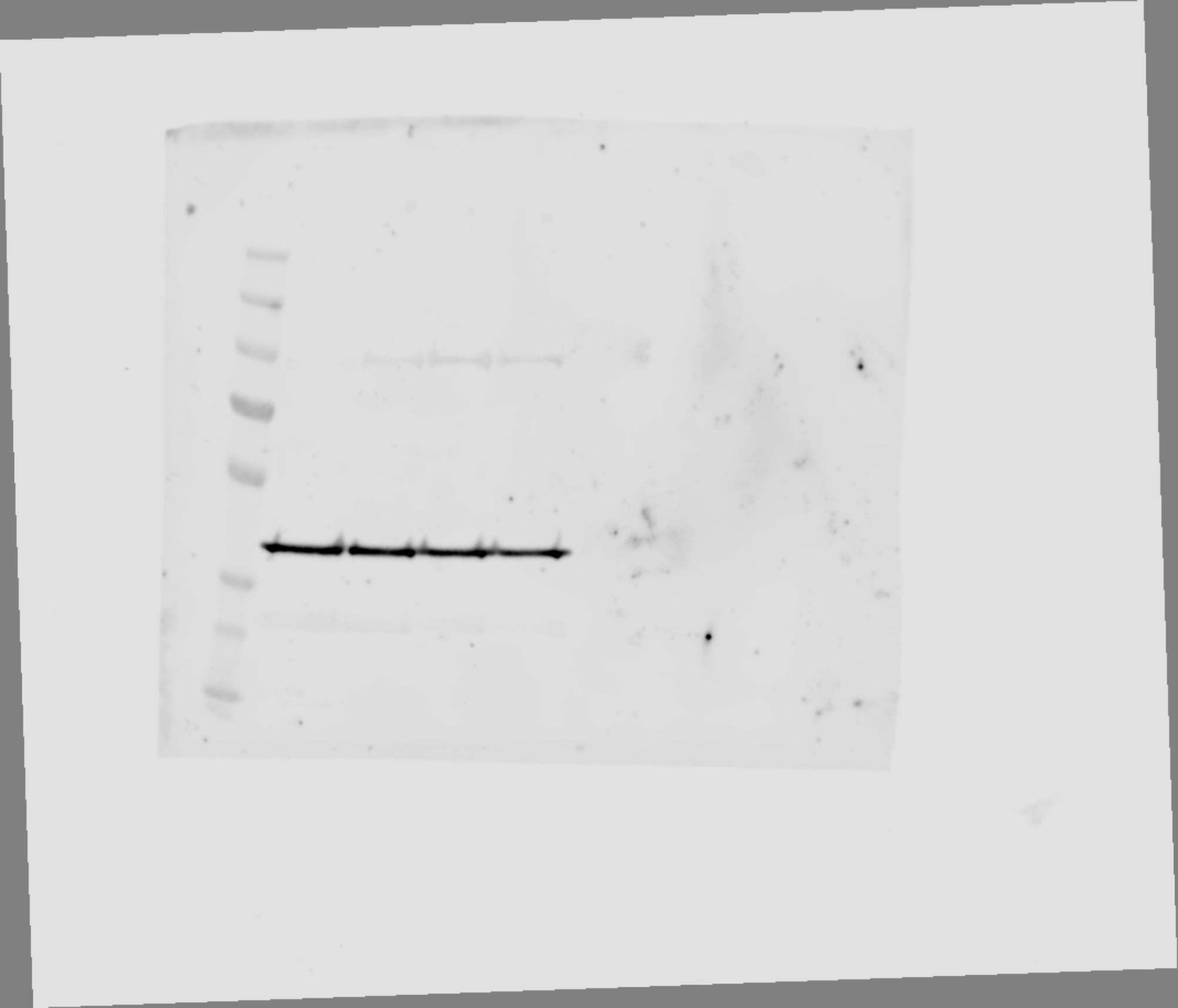

Supplement: Supplementary file 9 — Source Data for Figure 6 [file EMBR-24-e57571-s001.zip › figure 6/6e/original/CANDID~3.TIF]

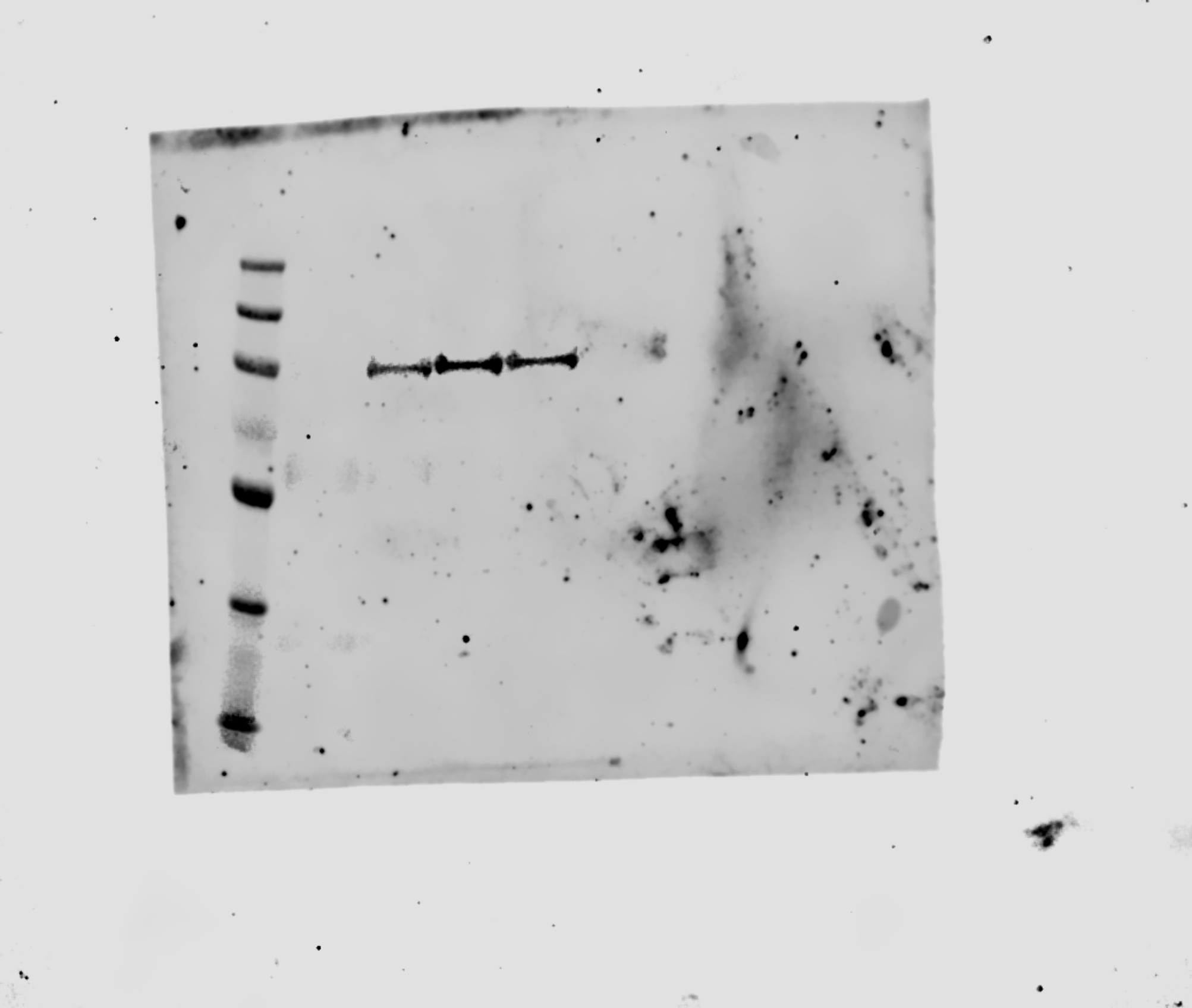

Supplement: Supplementary file 9 — Source Data for Figure 6 [file EMBR-24-e57571-s001.zip › figure 6/6e/original/CANDID~4.TIF]

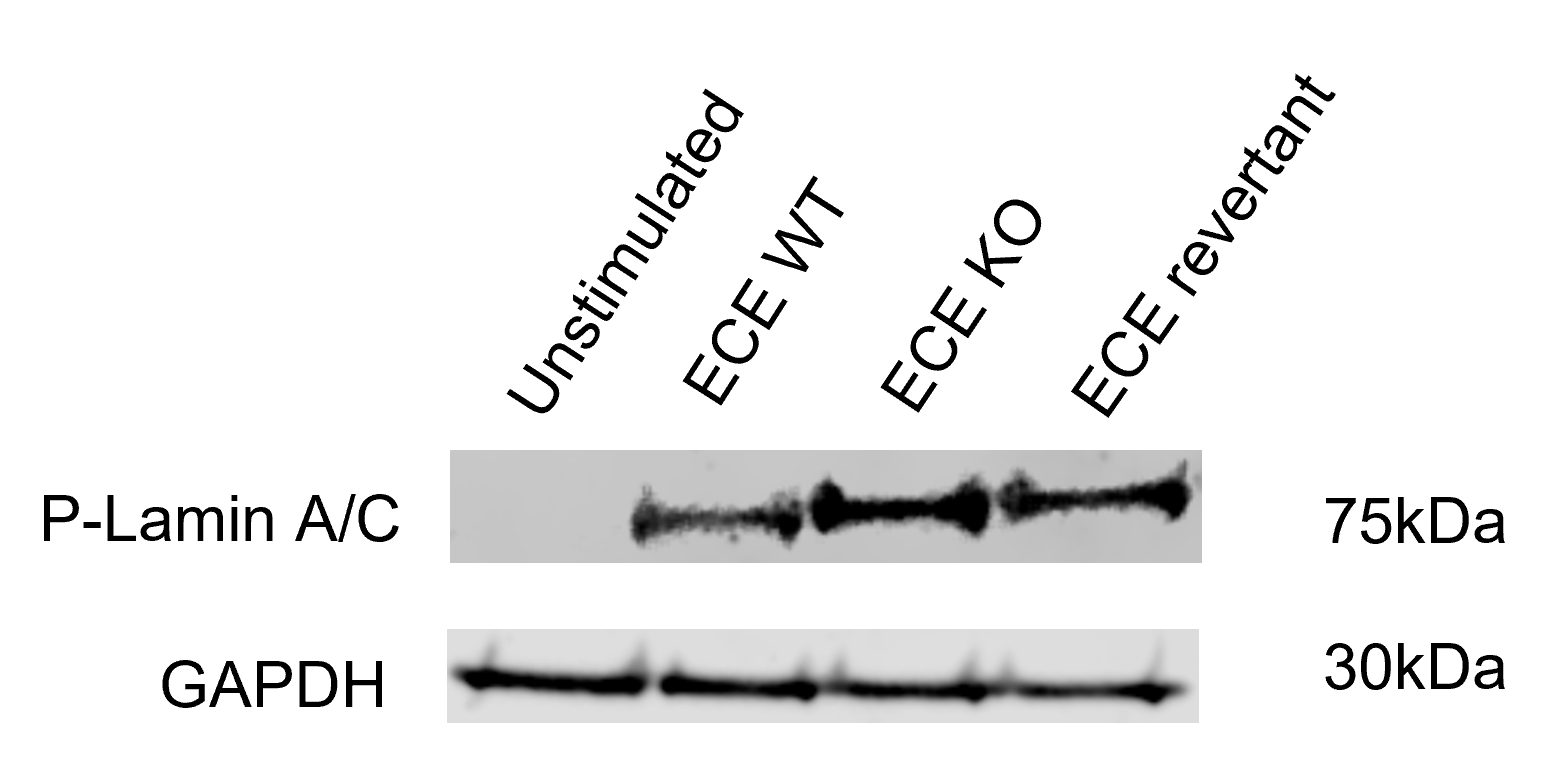

Supplement: Supplementary file 9 — Source Data for Figure 6 [file EMBR-24-e57571-s001.zip › figure 6/6e/prepared/p-lamin ECE MUTANT donor 1.tif]

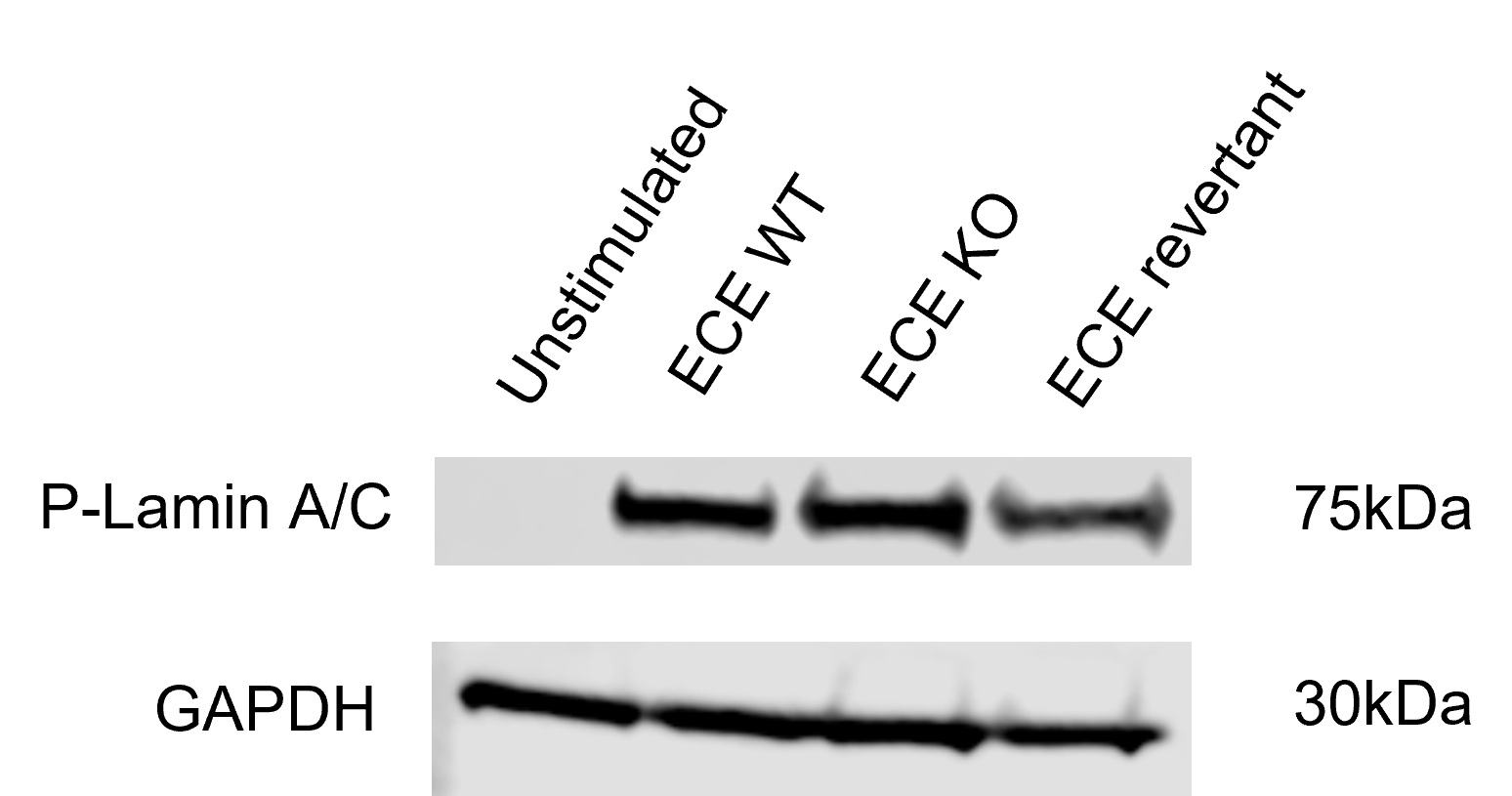

Supplement: Supplementary file 9 — Source Data for Figure 6 [file EMBR-24-e57571-s001.zip › figure 6/6e/prepared/p-lamin ECE MUTANT donor 2.tif]

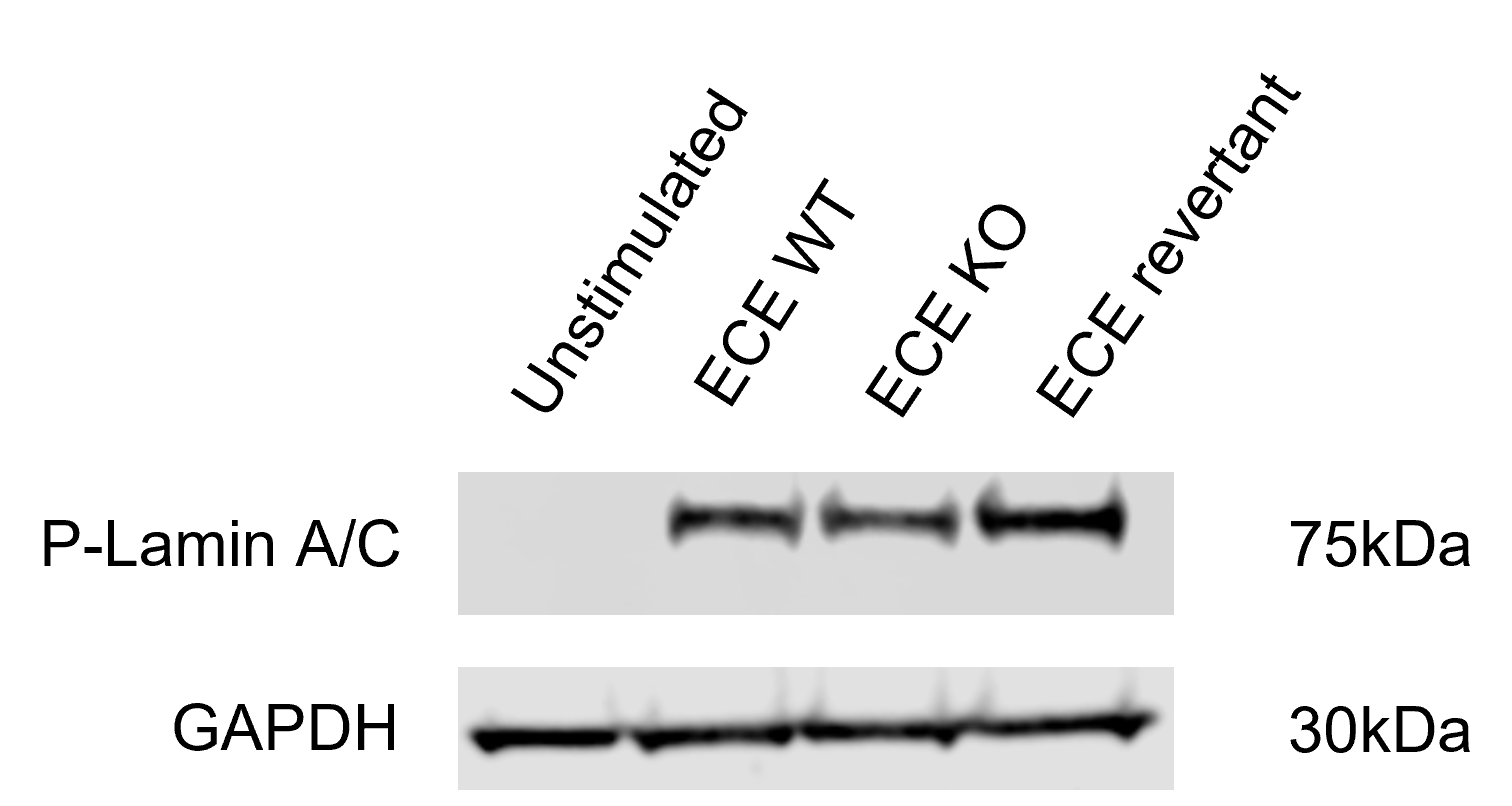

Supplement: Supplementary file 9 — Source Data for Figure 6 [file EMBR-24-e57571-s001.zip › figure 6/6e/prepared/p-lamin ECE MUTANT donor 3.tif]
