# Supplementary material for: Candida albicans induces neutrophil extracellular traps and leucotoxic hypercitrullination via candidalysin
Source: EMBO Rep. 2023 Oct 5;24(11):e57571. doi: 10.15252/embr.202357571 (PMC10626426; doi:10.15252/embr.202357571)
Supplement: Supplementary file 12 [file EMBR-24-e57571-s010.pdf]

EMBO reports

Candida albicans induces neutrophil extracellular traps and leukotoxic hypercitrullination via candidalysin

Samuel Skoluda, Emelie Backman, Borko Amulic, Fernando Ponce-Garcia, Chinelo Etiaba, Sujan Yellagunda, Renate Krüger, Horst Bernuth, Johan Bylund, Bernhard Hube, Julian Naglik, Lucas Unger, and Constantin Urban
DOI: 10.15252/embr.202357571

Corresponding author(s): Constantin Urban (constantin.urban@umu.se) , Lucas Unger (l.unger@aston.ac.uk)

Review Timeline:

Transfer from Review Commons:	31st May 23
Editorial Decision:	22nd Jun 23
Revision Received:	17th Aug 23
Editorial Decision:	24th Aug 23
Revision Received:	5th Sep 23
Accepted:	18th Sep 23

Editor: Achim Breiling

Transaction Report: **This manuscript was transferred to EMBO *reports* following peer review at Review Commons.**

Review #1

1. Evidence, reproducibility and clarity:

Evidence, reproducibility and clarity (Required)

This study evaluates the effect of fungal toxin candidalysin on neutrophils. The authors show that candidalysin induces NETosis when secreted by hyphae, but when candidalysin is added on its own, NLS are formed instead which are distinct from NETs. The authors have done lots of carefully controlled experiments, and delineated key components of the pathway inducing NLS, including the role of ROS and histone modifications. The data provided is high quality and well presented in figures.

2. Significance:

Significance (Required)

Strengths are the depth of analysis - many different aspects of NETosis is assessed and robustly tested.

Comments:

1. I was a bit confused by what should be the main message of the paper - is it that candidalysin on its own doesn't induce NETosis but only NLS? The answer to this question wasn't well addressed in my opinion, but the paper switches between using live fungi and purified candidalysin so it became confusing at times.
2. If candidalysin on its own only induces NLS - what is the relevance of this for disease? A lot of work has been provided on the pathway driving NLS formation, but it wasn't clear to me why this is important. More in discussion needed or evidence of disease relevance.
3. In Figure 2, it would be helpful to include images of ionomycin-stim neutrophils for comparison of the NLS structures across different stim conditions.
4. Few places where reference manager has failed (see bottom on page 10, line 190 for example)
5. Lines 191-198 - I was confused here by the text. I thought the point was that candidalysin induced NLS similar to ionomycin, but here the point is being made that the two are different? This led me to being confused as to the point of all the comparisons made between ionomycin NLS and candidalysin NLS... this could be made clearer.
6. Could the authors include some unstim neutrophil control images in Fig 3 for the

SEM? Can the SEM sample processing affect neutrophil structure in anyway? Feels like an important control although I don't have much experience with SEM personally.
7. I was very intrigued by the experiments where the authors added candidalysin in to neutrophils infected with ece1-null strain. Those experiments showed that candidalysin addition still drove NLS instead of NETosis. Can the authors investigate why this is? Is membrane intercalation different when candidalysin is delivered by hyphae vs added on its own? Could that explain some of the differences they have seen?
8. Is phagocytosis needed for NETosis induction by candidalysin? What happens if you add beads or beta-glucan particles with candidalysin stimulation? Do you get NLS or NETs?
9. Please confirm what the n numbers refer to in the figure legends - are these biological or technical replicates? How many experiments are the representative images representing?

3. How much time do you estimate the authors will need to complete the suggested revisions:

Estimated time to Complete Revisions (Required)

(Decision Recommendation)

Between 3 and 6 months

4. *Review Commons* values the work of reviewers and encourages them to get credit for their work. Select 'Yes' below to register your reviewing activity at Web of Science Reviewer Recognition Service (formerly Publons); note that the content of your review will not be visible on Web of Science.

Web of Science Reviewer Recognition

Yes

Review #2

1. Evidence, reproducibility and clarity:

Evidence, reproducibility and clarity (Required)

The study presented the role of cytolic fungal toxin - candidalysin, secreted by the hyphal form of Candida albicans, in the formation of neutrophil extracellular traps during C. albicans contact with neutrophils, which serve as the first line of the responses.
The key conclusions are convincing. The authors considered the whole mechanism of NLT formation, which explained previous observations made by others. Some of the additional proposed experiments are not necessary to perform. They would only complement the results already presented by the authors.
A few missing citations in the text need to be filled in. The others have been used appropriately and present earlier work on the subject. The reviewer indicated minor corrections to the drawings in the detailed comments.
This paper is very interesting and is crucial to understanding some unusual observations made earlier about NET production in fungal infections. On the other hand, the text requires minor corrections to understand better the occurrence of both forms of extracellular neutrophil traps.
The authors of the paper have experience in the subject matter presented, and the lead authors are among the leading researchers who analyze this problem. The presented research is a valuable addition to the previous work.

2. Significance:

Significance (Required)

The advantage of this work is the presentation of the mechanism associated with NLS formation in contact with candidalysin, where activation of NADPH oxidase and calcium influx have been documented to be important. This toxin can trigger ROS production and activate downstream signaling that is important for morphological changes and NLS formation. The important finding is also that NLS are resistant to nuclease treatment and increase the ability of neutrophils to control C. albicans hyphae formation and fungal cell growth. These findings provide a better understanding of the role of neutrophils in the treatment of infections caused by these microorganisms.

Below I present are minor suggestions that, in my opinion, will improve the text and

correct the presentation of the results, making this set of results a valuable source for explaining such a complex problem.

1. The authors should decide what thesis about NLS they want to prove:
100 NLS are less fibrous and .... than canonical NETs and are triggered in an NADPH oxidase-independent fashion.
121 NLS were dependent on NADPH oxidase-mediated reactive oxygen species (ROS) production
2. for the experiment described in the line below, MOI 2 was chosen; did the authors conduct an analysis of the response/eventual change in it, depending on the MOI?

131 we infected neutrophils with wild-type C. albicans, ECE1-deficient (ece1ΔΔ), and corresponding revertant (ece1ΔΔ+ECE1) strains,
3. Has the effect of deletion of ECE1 on other aspects of virulence, such as adhesion, virulence factor production, or biofilm formation, been analyzed?

137 the ECE1- and candidalysin-deficient strains triggered reduced levels
4. Fig.1 - How were C. albicans cells stained? Does 100%NET mean the number of cells netting after PMA treatment? This information should be given.
5. 168 dependent effect with increased NLS formation from 3 μM to 15 μM. However, the reduced NLS

How was determined the limiting concentration value of the toxin, for which an increase in NLS was observed? Was a wide range of concentrations used in the analysis or was the determination made only for these three selected values? A complete concentration analysis should be performed.
6. 169 formation was observed at 70 μM (Fig. 2b), which can be explained by neutrophil cell death induced by the toxin as determined by a DNA Sytox Green assay (Fig. S1a).

Was another viability test conducted? AnnexinV? Caspase 3/7? Sytox is not a specific staining in this regard. Furthermore, in Fig. S1a you state the kinetics of cell death, also after PMA treatment. On the one hand, you say that the production of candidalysin of NLS above 70 uM is reduced due to cell death, but at the same time you define as cell death the changes under PMA, which induce netosis. Please explain this reasoning better.
7. 175 mixing of granular and nuclear components at ~120 min after stimulation (Fig. 2d and Fig. S2).

Figure S2 does not show mixing with the content of the granules. You are not labeling any granule component, only histones. You cannot draw that conclusion from these

results.
8. Fig. 2. What concentration of PMA was used? What does 100% NLS mean? How is it different from 100% NET, since you are using PMA in both cases. Please explain.
9. 181 NLS were quantified with the same criteria as previous described for NETs.

The criterion for NETs was an area above 100um2, so what is the criterion for NLS? If we assume that this is the same as for NETs, then what is the difference between NLS and NETs? The criteria adopted do not differentiate between the two forms and appear to be subjective.
10. 190 allows a more detailed view of the neutrophil-derived structures (Error! Reference source not
Please, eliminate this error.
11. 193 Ionomycin has been previously reported to induce NLS, also...
194 Both, PMA and ionomycin generated widespread chromatin fibers in the extracellular space
197 In addition, C. albicans hyphae induced NETs with observable fibers and
198 threads similar to PMA- and ionomycin-stimulated neutrophils (Fig. 3b).
199 Image-based quantification of NLS events (candidalysin and ionomycin)

In a sentence earlier (193) you mentioned that the action of PMA leads to classical netosis and ionomycin leads to NLS. You pointed out earlier that NLS are poorly developed NETs (line 100), and here you write that PMA and ionomycin generate the same developed structures. You again differentiate between these structures depending on the stimulating factors. Pointing out the differences between the two forms, you should be more precise and consistent in your descriptions. This comment applies to the entire manuscript.
12. 203 NLS after 3 h and 5 h, respectively, and led to overall fewer NLS events. This was confirmed by observation.
204 area-based analysis of the events (Fig. 3d). The average area per event that exceeded 100 µm2 was
205 determined using the images from the DNA stain.
What is the accepted criterion for distinguishing between NLS and NETs?
13. line 218, 243 - reference error
14. What form are we actually talking about? Are we focusing on the effect of a natural agent or a synthetic one in relation to NLS/NET? Perhaps it is more important to focus on the citrullination process:

247 synthetic candidalysin only induces NLS, we concluded that candidalysin augments NET release when the toxin is secreted by C. albicans hyphae. 256 This confirmed that candidalysin promotes C. albicans-triggered NET release.
262 Interestingly, the addition of synthetic candidalysin resulted in a shift to NLS,

274 External addition of synthetic candidalysin resulted in a shift to NLS structures rather than NETs as visualized by microscopy after 5 h incubation (20X).
15. Has there been any method to track candidalysin production during contact of C. albicans with neutrophils?
16. In Figure 4f-the given information indicates 1,2 hour incubation, in the caption of the figure there is information about 5 hour incubation - please clarify. The description of the stains used is lacking.
17. Fig. 5 - result for 15 uM MitoTEMPO - adds nothing to the results and introduces image information noise - should be removed.
No information on the concentration of the peptide used.
18. Fig. 5, line 309: and cell-permeable Sytox Green DNA dye (250310 nM) to determine the total number of cells".

Please correct the information on the use of both dyes, according to the manufacturer's description:
"SYTOX® Green nucleic acid stain is an excellent green-fluorescent nuclear and chromosome counterstain that is impermeant to live cells, making it a useful indicator of dead cells within a population."
19. 324 At later time points, BAPTA-AM led to an increase in NLS, probably due to toxic effects as indicated by higher background levels of NLS formation in non-stimulated, BAPTA-AM-treated neutrophils (Fig. 6d).

If such an assumption is made, the toxic effect should also be observed for the control.
20. Fig. 6C PAD inhibitor should affect PMA-induced netosis, but the figure presents NLS existence - how was this change found?
21. line320 "This indicates that candidalysin most probably causes Ca2+ influx via pore formation and not via direct receptor stimulation"
And:
line 358. As C.albicans hyphae bind to pathogen recognition receptors (PRRs), activate neutrophils and ultimately promote the release of NETs, we aimed to elucidate whether candidalysin alone leads to the activation of similar pathways in neutrophils. Hence, we stimulated neutrophils with candidalysin in the presence or absence of specific inhibitors for SYK, PI3K, and Akt.

Lack of consistency in conclusion.
22. Fig. 7 It would be good to verify these results with experiments using mutants. Figures 7b, 7c, and 7d can be combined to make the whole drawing clearer.
23. line 603 'The percentage of dead cells was calculated using TritonX-100 lysed neutrophils as 100% control' - maybe use " treated or permeabilized"

3. How much time do you estimate the authors will need to complete the suggested revisions:

Estimated time to Complete Revisions (Required)

(Decision Recommendation)

Less than 1 month

4. *Review Commons* values the work of reviewers and encourages them to get credit for their work. Select 'Yes' below to register your reviewing activity at Web of Science Reviewer Recognition Service (formerly Publons); note that the content of your review will not be visible on Web of Science.

Web of Science Reviewer Recognition

No

Point-by-point response to reviewer comments for manuscript preprint RC-2022-01715

Reviewer #1 (Evidence, reproducibility and clarity (Required)):

This study evaluates the effect of fungal toxin candidalysin on neutrophils. The authors show that candidalysin induces NETosis when secreted by hyphae, but when candidalysin is added on its own, NLS are formed instead which are distinct from NETs. The authors have done lots of carefully controlled experiments, and delineated key components of the pathway inducing NLS, including the role of ROS and histone modifications. The data provided is high quality and well presented in figures.

Reviewer #1 (Significance (Required)):

Strengths are the depth of analysis - many different aspects of NETosis is assessed and robustly tested.

Comments:
1. I was a bit confused by what should be the main message of the paper - is it that candidalysin on its own doesn't induce NETosis but only NLS? The answer to this question wasn't well addressed in my opinion, but the paper switches between using live fungi and purified candidalysin so it became confusing at times.
Thank you for this important comment. We have clarified our narrative on candidalysin throughout the manuscript to provide a red thread for the readership. Our message is that candidalysin alone has not the capacity to induce a full cycle of signalling events which result in canonical NET formation. Our data show that candidalysin alone falls short and can only produce NLS. On the other hand, our data show that in the context of growing C. albicans cells candidalysin is able to promote the release of NETs. This is important, since previously the hyphal form of C. albicans has been reported to be a formidable inducer of NETs, whereas the yeast form was not. Our data put candidalysin in the centre of this observation, showing that it indeed is a major contributor to NET formation when present with growing C. albicans cells. Since candidalysin expression and release is strictly connected to hyphal growth our new data agrees with previous assessment and provides new insight in how this hyphae-specific inductive effect is accomplished.

In the revised manuscript, at first, we describe the difference of neutrophil stimulation when using strains expressing and lacking candidalysin as compared to candidalysin stimulation alone. We added or modified the following phrases:
*Line (163) "*As candidalysin-expressing *C. albicans* strains induced more NETs than candidalysin-deficient strains, we investigated the role of the toxin alone in stimulating neutrophil extracellular trap release".

Line (173) "In order to ensure consistency in NET/NLS quantification, NLS were quantified with the same criteria as previous described for NETs."

Line (182) "In summary, candidalysin alone triggers morphologically distinct NLS in a time- and dose-dependent manner, whereas candidalysin-producing *C. albicans* hyphae induce canonical NETs (Fig. 1a)."

Next, we describe the different morphology of NLS triggered by candidalysin alone in comparison to canonical NETs triggered by C. albicans strains expressing candidalysin. We added or modified the following phrases:

Line (198) "To investigate candidalysin-triggered NLS in further detail, we used scanning electron microscopy (SEM) that allows a more detailed view of the neutrophil-derived structures (Fig 3a)."

Furthermore, to prevent switching between experiments using candidalysin alone and experiments with different Candida strains, we have moved the next paragraph "Candidalysin-expressing strains induce more NETs and higher citrullination levels than candidalysin-deficient strains" to the end of the result section (old Fig. 4 is now new Fig. 8). In doing so, we focus on the direct morphological, signalling and functional effects of candidalysin alone on neutrophils and towards the end, we analyse how the strains are affected by different neutrophil killing mechanisms (phagocytosis and NETs). Subsequently, we synthetize our findings by showing that candidalysin is the main driver of histone citrullination by quantifying this histone modification in the context with NET induction comparing candidalysin-expressing and -deficient strains. We conclude that citrullination-induced chromatin decondensation in combination with candidalysin-induced ROS production are most probably the main contributors of increased NET formation stimulated by C. albicans hyphae expressing candidalysin. This is the also a good conclusion of the manuscript showing that candidalysin alone is not enough but together with growing C. albicans cells it contributes to NET induction and increased NETs in turn inhibit growth and limit spreading of C. albicans.

We modified and added the following sentences to the discussion section: (line 457) "The data suggests that candidalysin is the key driver of histone citrullination in neutrophils infected with *C. albicans* and that addition of evenly distributed, external candidalysin in high concentration (15 µM) drives neutrophils towards NLS despite the presence of *C. albicans* cells. We conclude that, during infection, candidalysin-triggered Ca^{2+} influx and histone hypercitrullination amplify processes in neutrophils which are induced by *C. albicans* hyphae. These amplified processes culminate in a strongly increased release of NETs that in turn are formidable weapons to control hyphal filaments."

2. If candidalysin on its own only induces NLS - what is the relevance of this for disease? A lot of work has been provided on the pathway driving NLS formation, but it wasn't clear to me why this is important. More in discussion needed or evidence of disease relevance.

Thank you for giving us the opportunity to clarify this issue. Candidalysin expression strongly increases with and is restricted to hyphal growth, which is the adhesive growth form of C. albicans. Given that epithelial cells expunge candidalysin for their own protection while hyphae remain attached, it could be possible that neutrophils get exposed to candidalysin before they encounter C. albicans cells. Therefore, it is relevant to understand how candidalysin per se shapes neutrophil responses. We have added the following sentences to the discussion section: (line 527) "As epithelial cells are able to expunge candidalysin for protection while *C. albicans* hyphae remain adeherent [46] recruited neutrophils may encounter candidalysin before direct contact with hyphae."

With regard to relevance for candidiasis the observation that candidalysin-deficient strains are poor inducers of NETs is most important. Since candidalysin expression is entirely restricted to hyphal growth. this finding gives crucial, new insight into the previous observation that hyphae are better NET inducers than yeast from C. albicans. In this context, we wanted to make it very clear to the reader that this effect only works when C. albicans cells and candidalysin are combined and that candidalysin alone does not lead to full-blown NET formation. Therefore, we have included a thorough investigation of the effects of candidalysin on neutrophils to be able to better contextualize our findings comparing candidalysin-expressing and candidalysin-deficient strains.

To make this point clearer. we added the following sentence to the summary at the end of the discussion section: (line 565) "Neutrophils encountering candidalysin-expressing hyphae are able to adequately respond by releasing increased amounts of NETs whereas secretion of candidalysin does not allow hyphae to evade from neutrophil attack."

In addition, we are convinced that the move of former Fig.4 to the end of the result section (now Fig. 8) additionally helps the reader to better understand the importance to first delineate the effect of

candidalysin on neutrophils alone and then to conclude the manuscript with experiments using different C. albicans strains to put the findings into context.

To add more substance to our conclusions we wrap up the new version of the manuscript with data comparing wild-type and candidalysin-deficient strains in neutrophil antimicrobial assays and quantification of histone citrullination. With the newly added antimicrobial assays we demonstrate that candidalysin expression does not affect phagocytic killing (Fig. 7d and 7e) as assessed by plating assays and that candidalysin does not affect inhibition by PMA-induced NETs (Fig. 7f and 7g). Thus, as stated above, during the interaction of hyphae and neutrophils candidalysin promotes the release of more NETs, but does otherwise not affect anti-Candida activity by neutrophils. Increased NETs in turn, however, inhibit growth and limit spreading of C. albicans. The manuscript now ends with the data on differences in histone citrullination when using wild-type and candidalysin-deficient strains indicating that citrullination-induced chromatin decondensation in combination with C. albicans cells ultimately leads to increased NET release.

We added the following text to the manuscript: (Line 416) "To corroborate, whether candidalysin deficiency affects *C. albicans'* susceptibility to neutrophil attack we performed two antimicrobial assays. In the first assay we determined NET-mediated anti-*Candida* activity by preformed NETs comparing wild-type and candidalysin-deficient strains. We used the same imaged-based analysis with calcofluor white staining. To be able to better observe differences in susceptibility of the different strains we used a slightly higher MOI than for the previous NET inhibition assays which explains higher survival percentage (Fig. 7c, black bars on the right side). As expected, candidalysin did not affect the inhibitory effect on *C. albicans* imposed by NETs (Fig. 7c). In the second assay, we determined short-term anti-*Candida* activity of intact neutrophils, which is predominantly phagocytic elimination, by serial dilution and plating for colony counts. Candidalysin-deficient and wild-type strains are killed similarly over the time of 1 to 4 h, both at MOI 1 and 3 (Fig. 7d and 7e). This indicates that candidalysin expression does not enable evasion from neutrophil phagocytic attack and this result agrees well with our previous finding that wild-type *C. albicans* engulfed by human neutrophils are unable to escape by hyphal outgrowth [16]. In conclusion, while candidalysin strongly increases the NET-inductive capacity of *C. albicans* hyphae, the toxin does neither affect the anti-*Candida* effect of intact neutrophils nor of NETs."

Notably, it is not informative to use C. albicans as inducer of NETs and as target of anti-Candida activity by NETs in the same assay, since both induction and anti-Candida activity are dependent on the amount of C. albicans cells. We therefore chose to show two separate assays where we (i) quantify short-term killing by plating (mainly phagocytosis) and (ii) quantify growth inhibition of C. albicans by pre-stimulated NETs.

3. In Figure 2, it would be helpful to include images of ionomycin-stim neutrophils for comparison of the NLS structures across different stim conditions.
This is a very good point. We supply a structural comparison between NLS and NETs induced by PMA, ionomycin and candidalysin in Figure 3. Additionally, the time-dependent changes for ionomycin are now included in the supplementary Figure S1.

4. Few places where reference manager has failed (see bottom on page 10, line 190 for example)
We have fixed this issue, thank you for pointing it out.

5. Lines 191-198 - I was confused here by the text. I thought the point was that candidalysin induced NLS similar to ionomycin, but here the point is being made that the two are different? This led me to being confused as to the point of all the comparisons made between ionomycin NLS and candidalysin NLS... this could be made clearer.

Thank you for highlighting this. According to previous literature ionomycin, a bacterial peptide toxin, was the most prominent example for induction of leukotoxic hypercitrullination. Therefore, we used ionomycin to put our findings with candidalysin, a fungal peptide toxin, into context. We find that candidalysin share similarities but also some striking differences to ionomycin. While we could not investigate the nature of these differences in more detail, this could be the basis of a follow-up study, we think it is important to give the reader the comparison in order to better understand how candidalysin shapes neutrophil responses. One clear difference which we show in the manuscript is that candidalysin induces some ROS whereas ionomycin does not at all (Fig. 4).

*We changed the text in the result section accordingly to make our point clearer: (line 203) "*PMA exposure generated widespread chromatin fibers in the extracellular space (Fig. 3a, left panels) whereas ionomycin exposure resulted in more compact, patchy areas occasionally dispersed with long, thin chromatin fibres (Fig. 3b, middle panels). With regard to morphological changes, candidalysin treatment resulted in compact, fibrous structures resembling those stemming from ionomycin treatment, however long, thread-like structures were absent in candidalysin-treated neutrophil samples (Fig. 3a right panels, for 7 h treatment see Fig. S1c)."

And did so as well in the discussion section: (Line 513) "While ionomycin- and candidalysin-induced NLS shared similar key features, such as increased histone citrullination, our study revealed striking differences between the two toxins. In contrast to ionomycin, candidalysin stimulation led to ROS production in neutrophils."

6. Could the authors include some unstim neutrophil control images in Fig 3 for the SEM? Can the SEM sample processing affect neutrophil structure in anyway? Feels like an important control although I don't have much experience with SEM personally.

This is of course a relevant control image. We have included an image showing unstimulated neutrophils from similar time points, but without exposure to candidalysin (Fig. 3). The unstimulated neutrophils are spherical and morphologically distinctly different from candidalysin-treated neutrophils.

7. I was very intrigued by the experiments where the authors added candidalysin in to neutrophils infected with ece1-null strain. Those experiments showed that candidalysin addition still drove NLS instead of NETosis. Can the authors investigate why this is? Is membrane intercalation different when candidalysin is delivered by hyphae vs added on its own? Could that explain some of the differences they have seen?

Thank you for this comment. Yes, there is a clear difference, since we add candidalysin to the medium such that the peptide is evenly distributed and reaches membranes rather evenly from the extracellular space. When released from growing C. albicans hyphae candidalysin is then predominantly released on hyphal tips as demonstrated in the referenced article (doi.org/10.1111/cmi.13378). Hyphal tips in turn are readily attacked by human neutrophils (doi.org/10.1189/jlb.0213063). Hence, we can safely assume according to these previous publications that there will be a more uneven distribution of candidalysin concentrations over neutrophil membranes, when the sole source of the toxin stems from growing hyphae interacting with neutrophils. It would of course be very interesting to know how the toxin exactly intercalates into membranes and which morphologies potential pores may have. These questions are currently under investigation in the laboratories of Profs Hube and Naglik. To include these findings here would certainly be far beyond the scope of this study.

We include and modify the following sentences to the discussion of this manuscript to clarify the issue: (Line 541). "One of the main goals of the study was to delineate contribution of candidalysin to neutrophil responses either as factor released by *C. albicans* hyphae or as singular peptide toxin. Our data demonstrates that candidalysin is the main driver of histone citrullination in neutrophils infected with *C. albicans* (Fig. 8). Lack of candidalysin production in *C. albicans* results in significantly reduced histone citrullination, accompanied with decreased NET formation. However, citrullination is not required for NET release, but rather governs the formation of NLS, which is dominant when candidalysin is added exogenously with even distribution throughout the cell suspension. With regard to *C. albicans* hyphae secreting candidalysin, local concentrations of the toxin are likely to vary to a large degree, particularly when the candidalysin-secreting hypha is engulfed by a neutrophil. Therefore, it may be difficult to discriminate NLS form NETs during the interaction of neutrophils and *C. albicans*, as both structures may be induced concurrently [10]. It seems logical that the pore-forming activity of candidalysin augments the release of NET fibres during *C. albicans* infection, where PRRs will additionally be triggered on neutrophils, resulting in combinatorial activation of downstream pathways. In line with this notion, candidalysin drives histone citrullination, which contributes to chromatin decondensation."

8. Is phagocytosis needed for NETosis induction by candidalysin? What happens if you add beads or beta-glucan particles with candidalysin stimulation? Do you get NLS or NETs?

This is an interesting question. Physical contact is required for the induction of NET formation (10.1111/j.1462-5822.2005.00659.x, 10.1371/journal.ppat.1000639) and physical contact leads to pattern recognition unequivocally followed by phagocytic events in neutrophils. Hence, at the least indirectly, phagocytosis and NET formation are connected, but may not be so causally.

While glucan-covered particles have been shown to induce NETs (10.1159/000365249), we show that C. albicans cells devoid of candidalysin induce NETs, but to a much lesser extent than wild-type C. albicans. In addition, the experiment shown in Fig. 8 shows exactly that. Instead of glucan-covered beats we used C. albicans cells (Fig. 8f) which by virtue are glucan covered.

9. Please confirm what the n numbers refer to in the figure legends - are these biological or technical replicates? How many experiments are the representative images representing?

Thank you very much for pointing this out. We adapted our figure legends accordingly and added the number of biological and technical replicates (n=x(y), x=biological replicates, y=technical replicates). Each experiment has been performed with at least three biological replicates which includes the use of different neutrophil donors.

Point-by-point response to reviewer comments for manuscript preprint RC-2022-01715

Reviewer #2 (Evidence, reproducibility and clarity (Required)):

The study presented the role of cytolic fungal toxin - candidalysin, secreted by the hyphal form of Candida albicans, in the formation of neutrophil extracellular traps during C. albicans contact with neutrophils, which serve as the first line of the responses.
The key conclusions are convincing. The authors considered the whole mechanism of NLT formation, which explained previous observations made by others. Some of the additional proposed experiments are not necessary to perform. They would only complement the results already presented by the authors.
A few missing citations in the text need to be filled in. The others have been used appropriately and present earlier work on the subject. The reviewer indicated minor corrections to the drawings in the detailed comments.
This paper is very interesting and is crucial to understanding some unusual observations made earlier about NET production in fungal infections. On the other hand, the text requires minor corrections to understand better the occurrence of both forms of extracellular neutrophil traps.
The authors of the paper have experience in the subject matter presented, and the lead authors are among the leading researchers who analyze this problem. The presented research is a valuable addition to the previous work.
Thank you very much for this positive and constructive assessment.

Reviewer #2 (Significance (Required)):

The advantage of this work is the presentation of the mechanism associated with NLS formation in contact with candidalysin, where activation of NADPH oxidase and calcium influx have been documented to be important. This toxin can trigger ROS production and activate downstream signaling that is important for morphological changes and NLS formation. The important finding is also that NLS are resistant to nuclease treatment and increase the ability of neutrophils to control C. albicans hyphae formation and fungal cell growth. These findings provide a better understanding of the role of neutrophils in the treatment of infections caused by these microorganisms.
Below I present are minor suggestions that, in my opinion, will improve the text and correct the presentation of the results, making this set of results a valuable source for explaining such a complex problem.
Thank you for this assessment. In cases which we have identified as crucial for our message we have decided to include additional experiments to better convey our message (Fig. 6e-f and Fig. 7d-g). We also included a time course for ionomycin stimulation of neutrophils in Fig. S1. We appreciate that the overall assessment was that no additional experiments were required.

1/ The authors should decide what thesis about NLS they want to prove:
100 NLS are less fibrous and .... than canonical NETs and are triggered in an NADPH oxidase-independent fashion.
121 NLS were dependent on NADPH oxidase-mediated reactive oxygen species (ROS) production
This was indeed imprecisely formulated from our side. NLS were previously described as NADPH-independent processes stimulated by toxins (see ionomycin). Candidalysin seems to trigger NADPH-dependent and NADPH-independent pathways. However, the main differentiation criteria were described through the hypercitrullination which we could observe for candidalysin. To clarify, we have modified the following sentence: (line 121) "In contrast to previously described stimuli of NLS, candidalysin induced NLS in partial dependence on NADPH oxidase-mediated reactive oxygen species (ROS) production, wheras PAD4-mediated histone citrullination could be observed as well. Notably, candidalysin alone failed to induce NETs as indicated by a lack of cell cycle activation determined via lamin A/C phosphorylation assays."

Point-by-point response to reviewer comments for manuscript preprint RC-2022-01715

2/ for the experiment described in the line below, MOI 2 was chosen; did the authors conduct an analysis of the response/eventual change in it, depending on the MOI?
Yes, from our experience in in vitro experiments with human neutrophils MOI<1 result in virtually complete phagocytosis of C. albicans and only minor amounts of NETs can be induced and at MOI>3 C. albicans overgrows too quickly. This is why an MOI 1-3 is the best option to analyse NET induction capacities.

131 we infected neutrophils with wild-type C. albicans, ECE1-deficient (ece1ΔΔ), and corresponding revertant (ece1ΔΔ+ECE1) strains,

3/ Has the effect of deletion of ECE1 on other aspects of virulence, such as adhesion, virulence factor production, or biofilm formation, been analyzed?
Yes indeed, the effect of candidalysin on other aspects has been studied. Candidalysin has no effect on adhesion and is expressed during biofilm formation. It has a broad effect on virulence in general and promotes neutrophil recruitment indirectly by a robust induction of damages responses. To clarify the amount of studies investigating these other aspects and to pinpoint the knowledge gap for direct interaction of neutrophils and candidalysin we include the following sentence: (line 132) "C. albicans hyphae release candidalysin and while the effects of the toxin for instance on virulence in general and on adhesion to host cells have been widely studied [17,18,23,28,30], the direct impact of candidalysin on the neutrophil immune response towards *C. albicans,* remains poorly understood. To investigate the role of candidalysin, we infected neutrophils with wild-type *C. albicans*,…"

137 the ECE1- and candidalysin-deficient strains triggered reduced levels

4/ Fig.1 - How were C. albicans cells stained? Does 100%NET mean the number of cells netting after PMA treatment? This information should be given.
Thank you for pointing this out. We were a bit unclear here. We added details in the respective figure legend and method section. C. albicans cells were visualised with anti-Candida antibody (1 µg/mL, ProSci, Cat#35-645). Furthermore, C. albicans nuclei are stained by DAPI, too.
100% NETs would mean that every single neutrophil (an image event which stains for neutrophil markers) in the analysed microscopic picture shows NET or NET-like morphology. We did not normalize to PMA treated cells.

5/ 168 dependent effect with increased NLS formation from 3 µM to 15 µM. However, the reduced NLS

How was determined the limiting concentration value of the toxin, for which an increase in NLS was observed? Was a wide range of concentrations used in the analysis or was the determination made only for these three selected values? A complete concentration analysis should be performed.
This is of course a valid point. We showed data on these concentrations as established from previous studies of our collaborators (10.1111/cmi.13378; 10.1038/nature17625; 10.1038/s41467-019-09915-2). Under 3 µM we did not observe much measurable results and therefore omitted these. Concentrations above 70 µM did not change the outcome anymore than at 70 µM, so higher concentrations were omitted. We, thus, show 3µM at which we see mild effects, show 15 µM (a 5-fold increase compared to 3µM) at which we see profound effects and show 70 µM (again approximately a 5-fold increase compared to 15 µM) at which we see an overwhelming effect. Additional concentrations in between the applied concentration values would not add much new information.

6/ 169 formation was observed at 70 µM (Fig. 2b), which can be explained by neutrophil cell death induced by the toxin as determined by a DNA Sytox Green assay (Fig. S1a).

Was another viability test conducted? AnnexinV? Caspase 3/7? Sytox is not a specific staining in this regard. Furthermore, in Fig. S1a you state the kinetics of cell death, also after PMA treatment. On the one hand, you say that the production of candidalysin of NLS above 70 uM is reduced due to cell death, but at the same time you define as cell death the changes under PMA, which induce netosis. Please explain this reasoning better.

Thank you for pointing this out. We have no indication that candidalysin stimulates apoptosis in neutrophils. Therefore, no AnnexinV/Caspase 3/7 stain was performed. What we wanted to emphasize is that at 70 µM candidalysin the cytotoxic character of candidalysin is overwhelming leading to rather quick cell death, as assessed by the Sytox assay. Sytox is specific in the regard that it determines whether the plasma membrane is permeable and gives the stain access to the nuclear DNA to result in a positive signal. We use this assay to quantify NET formation, since it is a quantitative assay and less laborious than microscopy. However, we always back up NET assays with microscopic, image-based analyses and do not use the Sytox assay as standalone experiment for NET quantification, since the Sytox assay is not specifically staining netting cells, but it also stains other types of cell death.

We clarify this in the text as follows: (line 659) "Neutrophil cell death or the presence of extracellular DNA was quantified using a Sytox Green-based (Invitrogen) fluorescence assay similar to previous descriptions [2,35]. To ultimately quantify NETs or NLS we always used image-based assys, the cell death assay was only used as complementation."

7/ 175 mixing of granular and nuclear components at ~120 min after stimulation (Fig. 2d and Fig. S2).

Figure S2 does not show mixing with the content of the granules. You are not labeling any granule component, only histones. You cannot draw that conclusion from these results.

We respectfully disagree. As indicated in the figure legend for Figure 2d we were labelling for neutrophil elastase (red) which is located in azurophilic granules and thereby presents a marker for granular content. Since we wrongfully referred to Figure S2 here, we removed this from the text. The latter reference probably remained erroneously from a previous version.

8/ Fig. 2. What concentration of PMA was used? What does 100% NLS mean? How is it different from 100% NET, since you are using PMA in both cases. Please explain.

We have now defined PMA concentration in the respective figure legend (100nM). The criteria for image-based assessment of NLS and NET quantification are the same for reason of comparison. PMA is included in each of the experiments as a positive control to show that the used neutrophils react upon stimulation. To clarify, we now specify at the y-axis %NETs or NLS. As stated above, 100% NLS means that each cell event in the image has increased in diameter such that it is considered as a NET or NLS. Hence, we use a common coordinate system to quantify extracellular events (NETs and NLS) based on size.

We have adjusted the figure legend as follows: (line 186) "**Fig 2. Candidalysin induces NLS in human neutrophils**. Candidalysin, but not scrambled candidalysin or pep2, another Ece1p-derived peptide (all 15 µM), induce (a) DNA decondensation in human neutrophils after 4 h (n = 4(10-14)) in a (b) dose-dependent manner (n = 3(10-14)). To allow comparability, NLS were quantified with the same criteria as previously described for NETs. Data shown as mean ± SEM. Confocal images (c) of immunostained cells display morphological changes involving nuclear and granular proteins after 4 h compared to unstimulated cells or 100 nM PMA, or cells exposed to scrambled candidalysin and

pep2. The morphological changes evoked by PMA considerably deviate from morphological changes evoked by candidalysin and, hence, are defined as NETs (for PMA) and NLS (for candidalysin). Time-dependent progression of morphological changes (d) in neutrophils induced by candidalysin over the course of 5 h (all images are with 60X magnification)."

9/ 181 NLS were quantified with the same criteria as previous described for NETs.

The criterion for NETs was an area above 100um2, so what is the criterion for NLS? If we assume that this is the same as for NETs, then what is the difference between NLS and NETs? The criteria adopted do not differentiate between the two forms and appear to be subjective.
As stated above, for us it was very important to find a common coordinate system to quantify NETs and NLS, since we wanted to deliver comparable and solid quantitative data. Hence, the quantification method does not discriminate between NETs and NLS. The notable morphological differences of NETs and NLS are thoroughly described with Figure 2 and Figure 3 and defined by differences in their structure. In addition, we present differences and similarities of induced pathways leading to canonical NETs or candidalysin-induced NLS in Figure 6 and Figure 7. We are convinced that, since NETs and NLS vary in size (DNA area covered), it will not be accurate for quantification purposes to include an additional size cut-off in the attempt to discriminate NLS and NETs. Instead we have established that candidalysin alone induces morphologically distinct NLS, whereas Candida albicans hyphae induce morphologically distinct NETs. By combination of quantitative data and image-based assessment, both structures can be discriminated from each other. In addition, we have established that during neutrophil and C. albicans interaction, citrullination of histone mainly stems from candidalysin. We show here and others have shown previously (10.3389/fimmu.2018.01573) that citrullination of histone occurs during but is not required for NET formation. But histone citrullination is promoted mainly by candidalysin and is also required for formation of NLS. Thus, histone citrullination constitutes another important discriminatory factor between NETs and NLS.

We added modified and added text to the respective figure legend: (line 188) "To allow comparability, NLS were quantified with the same criteria as previously described for NETs. Data shown as mean ± SEM. Confocal images (c) of immunostained cells display morphological changes involving nuclear and granular proteins after 4 h compared to unstimulated cells or 100 nM PMA, or cells exposed to scrambled candidalysin and pep2. The morphological changes evoked by PMA considerably deviate from morphological changes evoked by candidalysin and, hence, are defined as NETs (for PMA) and NLS (for candidalysin)."

10/ 190 allows a more detailed view of the neutrophil-derived structures (Error! Reference source not
Please, eliminate this error.
Thank you for pointing this out to us. We have fixed this error.

11/ 193 Ionomycin has been previously reported to induce NLS, also...
194 Both, PMA and ionomycin generated widespread chromatin fibers in the extracellular space
197 In addition, C. albicans hyphae induced NETs with observable fibers and
198 threads similar to PMA- and ionomycin-stimulated neutrophils (Fig. 3b).
199 Image-based quantification of NLS events (candidalysin and ionomycin)

In a sentence earlier (193) you mentioned that the action of PMA leads to classical netosis and ionomycin leads to NLS. You pointed out earlier that NLS are poorly developed NETs (line 100), and here you write that PMA and ionomycin generate the same developed structures. You again differentiate between these structures depending on the stimulating factors. Pointing out the

differences between the two forms, you should be more precise and consistent in your descriptions. This comment applies to the entire manuscript.

Thank you, we agree that consistency and clarity is required to describe the observed phenomena. We therefore modified or included the following sentences to the manuscript:

(line 203) ”PMA exposure generated widespread chromatin fibres in the extracellular space (Fig. 3a, left panels) whereas ionomycin exposure resulted in more compact, patchy areas occasionally dispersed with long, thin chromatin fibres (Fig. 3b, middle panels). With regard to morphological changes, candidalysin treatment resulted in compact, fibrous structures resembling those stemming from ionomycin treatment, however long, thread-like structures were absent in candidalysin-treated neutrophil samples (Fig. 3a right panels, for 7 h treatment see Fig. S1c)”

(Line 513) ”While ionomycin- and candidalysin-induced NLS shared similar key features, such as increased histone citrullination, our study revealed striking differences between the two toxins. In contrast to ionomycin, candidalysin stimulation led to ROS production in neutrophils.”

12/ 203 NLS after 3 h and 5 h, respectively, and led to overall fewer NLS events. This was confirmed by observation.
204 area-based analysis of the events (Fig. 3d). The average area per event that exceeded 100 µm2 was
205 determined using the images from the DNA stain.
What is the accepted criterion for distinguishing between NLS and NETs?

The main criteria distinguishing canonical NETs from NLS is a higher compactness for NLS and an increased citrullination of histones, the latter being absent in canonical NETs (10.3389/fimmu.2016.00461; 10.1016/j.mib.2020.09.011). Please see our comment above (regarding reviewer comment 9). Comparing candidalysin and ionomycin as stimuli for NLS they share key similarities, such as increased citrullination of histone (Fig. 3) and more compact structures than NETs (Fig. 3) with an average size of 151 µm2 for candidalysin-induced and 149 µm2 for ionomycin-induced NLS compared to 262 µm2 for PMA-induced and 231 µm2 for C. albicans-induced NETs (for clarification these average sizes are stated in the text). However, the NLS triggered by candidalysin and ionomycin also show differences. Ionomycin occasionally results in extended chromatin threads, whereas candidalysin does not. Ionomycin induces no ROS at all, whereas candidalysin does to some extent. By consistent usage of the definitions for NETs and NLS and by pinpointing the differences between ionomycin and candidalysin in terms of NLS induction (which are previously unknown) we hope we have sufficiently addressed this comment.

13/ line 218, 243 - reference error

Thank you, we have fixed this error

14/ What form are we actually talking about? Are we focusing on the effect of a natural agent or a synthetic one in relation to NLS/NET? Perhaps it is more important to focus on the citrullination process.

247 synthetic candidalysin only induces NLS, we concluded that candidalysin augments NET release when the toxin is secreted by C. albicans hyphae. 256 This confirmed that candidalysin promotes C. albicans-triggered NET release.
262 Interestingly, the addition of synthetic candidalysin resulted in a shift to NLS,
274 External addition of synthetic candidalysin resulted in a shift to NLS structures rather than NETs as visualized by microscopy after 5 h incubation (20X).

We used the adjective “synthetic” here to make clear that this is a synthetized peptide and not candidalysin isolated from growing C. albicans. Having said that, we fully agree that the synthetized peptide and the one released by C. albicans cells are essentially identical on the molecular level and

thus it is irrelevant and confusing to state in this context here. Therefore, we removed the adjective "synthetic" throughout the study and refer the reader to the method section for information on the origin of candidalysin used in the study. At times, we state "candidalysin alone" when we want to emphasize that candidalysin was the sole trigger used for the respective assay.

15/ Has there been any method to track candidalysin production during contact of C. albicans with neutrophils?

Thank you for this comment. Yes, there is a QVQ nanobody that can be used which is currently not to our disposal (doi.org/10.1111/cmi.13378). However, we already know from this publication that candidalysin concentrations vary when released naturally. The concentrations are particularly high in invasion pockets or dense biofilms. We also know that if we add candidalysin to the medium we have even distribution throughout and this is by definition different from concertation spikes at host cell-fungal interaction sites. As we have stated above, hyphal tips in turn are readily attacked by human neutrophils (doi.org/10.1189/jlb.0213063). Hence, we can safely assume, according to these previous publications, that there will be a more uneven distribution of candidalysin concentrations over neutrophil membranes, when the sole source of the toxin stems from growing hyphae interacting with neutrophils. It would of course be very interesting to know how the toxin exactly intercalates into membranes and which morphologies potential pores may have. These questions are currently under investigation in the laboratories of B. Hube and J. Naglik. To incorporate these findings here would certainly be far beyond the scope of this study.

We include and modify the following sentences to the discussion of this manuscript to clarify the issue: (Line 544). " Lack of candidalysin production in *C. albicans* results in significantly reduced histone citrullination, accompanied with decreased NET formation. However, citrullination is not required for NET release, but rather governs the formation of NLS, which is dominant when candidalysin is added exogenously with even distribution throughout the cell suspension. With regard to *C. albicans* hyphae secreting candidalysin, local concentrations of the toxin are likely to vary to a large degree, particularly when the candidalysin-secreting hypha is engulfed by a neutrophil. Therefore, it may be difficult to discriminate NLS form NETs during the interaction of neutrophils and *C. albicans*, as both structures may be induced concurrently [10]. It seems logical that the pore-forming activity of candidalysin augments the release of NET fibres during *C. albicans* infection, where PRRs will additionally be triggered on neutrophils, resulting in combinatorial activation of downstream pathways. In line with this notion, candidalysin drives histone citrullination, which contributes to chromatin decondensation."

16/ In Figure 4f-the given information indicates 1,2 hour incubation, in the caption of the figure there is information about 5 hour incubation - please clarify. The description of the stains used is lacking.

Microscopic analysis performed after 5h incubation time, whereas *candidalysin has been added to different time points indicated in the* Figure (in the new version this is now Figure 8f). We clarified in the legend as follows: (line 472) "(f) Neutrophils were infected with *C. albicans* and 15 µM candidalysin was added 0 h, 1 h or 2 h after the infection. Addition of candidalysin at the different time points after *C. albicans* infection resulted in a shift to NLS structures rather than NETs as visualized by microscopy after 5 h total incubation (20X)." *The description of the strains is depicted directly in the Figure, next to the microscopic images.*

17/ Fig. 5 - result for 15 uM MitoTEMPO - adds nothing to the results and introduces image information noise - should be removed.

No information on the concentration of the peptide used.

We would like to keep the 15 µM MitoTEMPO concentration, since it is the more reasonable concentration at which we do not observe an effect. This argues that ROS is more-likely derived from

NADPH oxidase and not mitochondrial ROS. We show TEMPOL effects at 15 µM and at 100 µM to document the dose dependency and for the sake of comparability, we would like to keep both concentrations also for MitoTEMPO.

The indicated peptide concentration was added to the figure legend. Thank you for pointing this out.

18 / Fig. 5, line 309: and cell-permeable Sytox Green DNA dye (250310 nM) to determine the total number of cells".

Please correct the information on the use of both dyes, according to the manufacturer's description:
"SYTOX® Green nucleic acid stain is an excellent green-fluorescent nuclear and chromosome counterstain that is impermeant to live cells, making it a useful indicator of dead cells within a population."
Thank you for highlighting this error. Indeed, we used Syto Green for this particular staining, a dye which stains both live and dead cells since the dye is cell-permeable. We corrected the error at this section of the text.

19/ 324 At later time points, BAPTA-AM led to an increase in NLS, probably due to toxic effects as indicated by higher background levels of NLS formation in non-stimulated, BAPTA-AM-treated neutrophils (Fig. 6d).

If such an assumption is made, the toxic effect should also be observed for the control.
The toxic effect was observed while conducting the experiments, but cannot be seen in the size-base quantification which is the read out for this particular experiment. We have performed a cytotoxicity assay using flow cytometry and PI staining to confirm the effect. The results are added as supplemental Figure (Fig. S3b).

20/Fig. 6C PAD inhibitor should affect PMA-induced netosis, but the figure presents NLS existence - how was this change found?
We are grateful for the opportunity to explain this more thoroughly. PMA does not trigger histone citrullination (10.3389/fimmu.2016.00461) and thereby there is no effect of the PAD inhibitor on PMA-induced NETs. Notably, some level of histone citrullination can also be observed in unstimulated neutrophils (see Fig. 3, 5 and 8), since histone modification is not exclusively dependent on stimulation. However, upon PMA stimulation we observe a decrease (Fig. S1b), not an increase, of histone citrullination consistent with previous reports.

We adjusted the text as follows: (line 235) ". Expectedly, citH3 levels upon PMA stimulation did not increase, but rather decreased which is consistent with previous reports [10] (Fig. 3d and Suppl. Fig. S1b). While citrullination levels in unstimulated neutrophils decreased over time, ionomycin stimulation sustained high levels over 5 h.

21/ line320 "This indicates that candidalysin most probably causes Ca2+ influx via pore formation and not via direct receptor stimulation"
And:
line 358. As C.albicans hyphae bind to pathogen recognition receptors (PRRs), activate neutrophils and ultimately promote the release of NETs, we aimed to elucidate whether candidalysin alone leads to the activation of similar pathways in neutrophils. Hence, we stimulated neutrophils with candidalysin in the presence or absence of specific inhibitors for SYK, PI3K, and Akt.

Lack of consistency in conclusion.

Thank you for pointing this out. We adjusted the paragraph (line 331) as follows: "As *C. albicans* hyphae bind to pathogen recognition receptors (PRRs), activate neutrophils and ultimately promote the release of NETs, we aimed to elucidate whether candidalysin alone can trigger similar pathways in neutrophils via signalling cross talk induced by Ca^{2+} influx. Hence, we stimulated neutrophils with candidalysin in the presence or absence of specific inhibitors for SYK, PI3K, and Akt (Fig. 6b)."

22/ Fig. 7 It would be good to verify these results with experiments using mutants. Figures 7b, 7c, and 7d can be combined to make the whole drawing clearer.

We thought this is very relevant and included additional experiments showing that the mutant strains also induce phosphorylation of lamin A/C independent of the expression of candidalysin (new Fig. 6e and 6f).

23/ line 603 'The percentage of dead cells was calculated using TritonX-100 lysed neutrophils as 100% control' - maybe use " treated or permeabilized"

Thank you, we changed the phrasing accordingly.

Dear Dr. Urban,

Thank you for the transfer of your revised manuscript to our editorial offices. I have now received the report from one of the two Review Commons referees that I asked to re-evaluate your study, you will find below. The second referee was completely unresponsive to my invitations to re-assess the study. However, going through your point-by-point-response, I consider his/her points as adequately addressed. Moreover, as you will see, referee #1 now fully supports publication of your study in EMBO reports.

Before I can proceed with formal acceptance, the manuscript needs formatting according to our journal style. Please carefully review the instructions that follow below.

PLEASE NOTE THAT upon resubmission revised manuscripts are subjected to an initial quality control prior to exposition to re-review. Upon failure in the initial quality control, the manuscripts are sent back to the authors, which may lead to delays. Frequent reasons for such a failure are the lack of the data availability section (please see below) and the presence of statistics based on n=2 (the authors are then asked to present scatter plots or provide more data points).

When submitting your final revised manuscript, we will require:

1) a .docx formatted version of the final manuscript text (including legends for main figures, EV figures and tables), but without the figures included. Figure legends should be compiled at the end of the manuscript text.

2) individual production quality figure files as .eps, .tif, .jpg (one file per figure), of main figures and EV figures. Please upload these as separate, individual files upon re-submission.

The Expanded View format, which will be displayed in the main HTML of the paper in a collapsible format, has replaced the Supplementary information. You can submit up to 5 images as Expanded View. Please follow the nomenclature Figure EV1, Figure EV2 etc. The figure legend for these should be included in the main manuscript document file in a section called Expanded View Figure Legends after the main Figure Legends section. Additional Supplementary material should be supplied as a single pdf file labeled Appendix. The Appendix should have page numbers and needs to include a table of content on the first page (with page numbers) and legends for all content. Please follow the nomenclature Appendix Figure Sx, Appendix Table Sx etc. throughout the text, and also label the figures and tables according to this nomenclature.

For more details, please refer to our guide to authors:
http://www.embopress.org/page/journal/14693178/authorguide#manuscriptpreparation

Please consult our guide for figure preparation:
http://wol-prod-cdn.literatumonline.com/pb-assets/embo-site/EMBOPress_Figure_Guidelines_061115-1561436025777.pdf

See also the guidelines for figure legend preparation:
https://www.embopress.org/page/journal/14693178/authorguide#figureformat

3) a complete author checklist, which you can download from our author guidelines (https://www.embopress.org/page/journal/14693178/authorguide). Please insert page numbers in the checklist to indicate where the requested information can be found in the manuscript. The completed author checklist will also be part of the RPF.

Please also follow our guidelines for the use of living organisms, and the respective reporting guidelines:
http://www.embopress.org/page/journal/14693178/authorguide#livingorganisms

4) that primary datasets produced in this study (e.g. RNA-seq, ChIP-seq, structural and array data) are deposited in an appropriate public database. If no primary datasets have been deposited, please also state this in a dedicated section (e.g. 'No primary datasets have been generated and deposited'), see below.

See also: http://embor.embopress.org/authorguide#datadeposition

Please remember to provide a reviewer password if the datasets are not yet public.

The accession numbers and database should be listed in a formal "Data Availability" section (placed after Materials & Methods) that follows the model below. This is now mandatory (like the COI statement). Please note that the Data Availability Section is restricted to new primary data that are part of this study. This section is mandatory. As indicated above, if no primary datasets have been deposited, please state this in this section

Data availability

The datasets produced in this study are available in the following databases:

- RNA-Seq data: Gene Expression Omnibus GSE46843 (https://www.ncbi.nlm.nih.gov/geo/query/acc.cgi?acc=GSE46843)
- [data type]: [name of the resource] [accession number/identifier/doi] ([URL or identifiers.org/DATABASE:ACCESSION])

*** Note - All links should resolve to a page where the data can be accessed. ***

Moreover, I have these editorial requests:

5) We now request the publication of original source data with the aim of making primary data more accessible and transparent to the reader. Our source data coordinator has already contacted you to discuss which figure panels we would need source data for and will also provide you with helpful tips on how to upload and organize the files. Please do that when you submit your final revised manuscript files.

6) Our journal encourages inclusion of *data citations in the reference list* to directly cite datasets that were re-used and obtained from public databases. Data citations in the article text are distinct from normal bibliographical citations and should directly link to the database records from which the data can be accessed. In the main text, data citations are formatted as follows: "Data ref: Smith et al, 2001" or "Data ref: NCBI Sequence Read Archive PRJNA342805, 2017". In the Reference list, data citations must be labeled with "[DATASET]". A data reference must provide the database name, accession number/identifiers and a resolvable link to the landing page from which the data can be accessed at the end of the reference. Further instructions are available at: http://www.embopress.org/page/journal/14693178/authorguide#referencesformat

7) Regarding data quantification and statistics, please make sure that the number "n" for how many independent experiments were performed, their nature (biological versus technical replicates), the bars and error bars (e.g. SEM, SD) and the test used to calculate p-values is indicated in the respective figure legends (also for potential EV figures and all those in the final Appendix). Please also check that all the p-values are explained in the legend, and that these fit to those shown in the figure. Please provide statistical testing where applicable. Please avoid the phrase 'independent experiment', but clearly state if these were biological or technical replicates. Please also indicate (e.g. with n.s.) if testing was performed, but the differences are not significant. In case n=2, please show the data as separate datapoints without error bars and statistics. See also: http://www.embopress.org/page/journal/14693178/authorguide#statisticalanalysis

If n<5, please show single datapoints for diagrams.

8) Please also note our reference format:
http://www.embopress.org/page/journal/14693178/authorguide#referencesformat

9) For microscopic images, please add scale bars of similar style and thickness to all the microscopic images, using clearly visible black or white bars (depending on the background). Please place these in the lower right corner of the images. Please do not write on or near the bars in the image but define the size in the respective figure legend.

10) We updated our journal's competing interests policy in January 2022 and request authors to consider both actual and perceived competing interests. Please review the policy https://www.embopress.org/competing-interests and update your competing interests if necessary. Please name this section 'Disclosure and Competing Interests Statement' and put it after the Acknowledgements section.

11) We now use CRediT to specify the contributions of each author in the journal submission system. CRediT replaces the author contribution section. Please use the free text box to provide more detailed descriptions and do not add author contributions to the manuscript text file. See also guide to authors:
https://www.embopress.org/page/journal/14693178/authorguide#authorshipguidelines

12) Please reduce the number of keywords to 5 and order the manuscript sections like this, using these names:
Title page - Abstract - Keywords - Introduction - Results - Discussion - Materials and Methods - Data availability section - Acknowledgements - Disclosure and Competing Interests Statement - References - Figure legends - Expanded View Figure legends - Tables

13) Please provide a title of not more than 100 characters (including spaces).

14) Please include funding information into the acknowledgements. Please enter all the funding information also into our submission system and make sure this is complete and similar to the one mentioned in the manuscript text file.

15) Please provide an abstract with not more than 175 words, written in present tense throughout.

In addition, I would need from you:

- a short, two-sentence summary of the manuscript (not more than 35 words).
- three to four short one sentence bullet points highlighting the key findings of your study.
- a schematic summary figure (synopsis image) in jpeg or tiff format with the exact width of 550 pixels and a height of not more than 400 pixels that can be used as a visual synopsis on our website.

I look forward to seeing a revised version of your manuscript when it is ready. Please let me know if you have questions or comments regarding the revision.

Best,

Achim Breiling
Senior Editor
EMBO Reports

Referee #1:

I am happy with the revisions made by the reviewers which I think help clarify the earlier confusions and make the paper easier to read and follow.

***
Rev_Com_number: RC-2022-01715
New_manu_number: EMBOR-2023-57571V1
Corr_author: Urban
Title: Candida albicans promotes neutrophil extracellular trap formation and leukotoxic hypercitrullination via the peptide toxin candidalysin.

The authors have addressed all minor editorial requests.

1st Revision - Editorial Decision 24th Aug 2023

Dear Prof. Urban,

Thank you for the submission of your revised manuscript to our editorial offices. Before I can proceed with formal acceptance, I have these few further editorial requests I ask you to address in a final revised manuscript:

- We updated our journal's competing interests policy in January 2022 and request authors to consider both actual and perceived competing interests. Please review the policy https://www.embopress.org/competing-interests and update your competing interests if necessary. Please name this section 'Disclosure and Competing Interests Statement' and put it after the Acknowledgements section.

- Please format the references and their callouts according to our journal style: http://www.embopress.org/page/journal/14693178/authorguide#referencesformat

- Please remove the synopsis blurb and the bullet points from the Figure legends. I have attached these here as a word file. Please go through this again and note that the synopsis blurb should not have more than 35 words and the up to 4 bullet points not more than 2 lines.

- Please make sure that the number "n" for how many independent experiments were performed, their nature (biological versus technical replicates), the bars and error bars (e.g. SEM, SD) and the test used to calculate p-values is indicated in the respective figure legends (main and EV figures). Please also check that all the p-values are explained in the legend, and that these fit to those shown in the figure. Please provide statistical testing where applicable. Please avoid the phrase 'independent experiment', but clearly state if these were biological or technical replicates. Please also indicate (e.g. with n.s.) if testing was performed, but the differences are not significant. In case n=2, please show the data as separate datapoints without error bars and statistics (see e.g. panel 7C and EV4A). See also: http://www.embopress.org/page/journal/14693178/authorguide#statisticalanalysis

If n<5, please show single datapoints for diagrams.

Presently, the number of replicates in the legends is often given as, e.g., n = 4 (10-14). Please explain this better in the legends. Are these 4 biological replicates with 10-14 technical replicates?

- Please move the table and its legend to the end of the manuscript text file (after the EV figure legends).

- Please add a formal "Data Availability" section (placed after Materials & Methods) to the manuscript text file. This is now mandatory (like the COI statement). Please add there information on all the image files deposited at BioStudies and provide a link.

- Please upload the movie file ZIPd together with a text file with the legend. Then please remove the movie legend from the manuscript file.

- Please complete the attached SD Checklist (regarding Fig. 8E and 8F) and also mention there which images have been deposited at BioStudies.

- Please make sure that all figure panels are called out separately and sequentially (main and EV items). Presently, there seem to be no separate callouts for Fig. 4D and 6E&F. Moreover, Fig 5A is called out after 5D. Please check.

- Finally, please find attached a word file of the manuscript text (provided by our publisher) with changes we ask you to include in your final manuscript text, and some queries, we ask you to address. Please provide your final manuscript file with track changes, in order that we can see any modifications done.

I look forward to seeing the final revised version of your manuscript when it is ready. Please let me know if you have questions regarding the revision.

Please use this link to submit your revision: https://embor.msubmit.net/cgi-bin/main.plex

Best,

Achim Breiling
Senior Editor
EMBO Reports

The authors have addressed all minor editorial requests.

2nd Revision - Editorial Decision 18th Sep 2023

Prof. Constantin Urban
Umeå University
Clinical Micropbiology
Sjukhusområdet 6C
Umeå 90185
Sweden

Dear Prof. Urban,

I am very pleased to accept your manuscript for publication in the next available issue of EMBO reports. Thank you for your contribution to our journal.

At the end of this email I include important information about how to proceed. Please ensure that you take the time to read the information and complete and return the necessary forms to allow us to publish your manuscript as quickly as possible.

As part of the EMBO publication's Transparent Editorial Process, EMBO reports publishes online a Review Process File to accompany accepted manuscripts. As you are aware, this File will be published in conjunction with your paper and will include the referee reports, your point-by-point response and all pertinent correspondence relating to the manuscript.

If you do NOT want this File to be published, please inform the editorial office within 2 days, if you have not done so already, otherwise the File will be published by default [contact: emboreports@embo.org]. If you do opt out, the Review Process File link will point to the following statement: "No Review Process File is available with this article, as the authors have chosen not to make the review process public in this case."

Thank you again for your contribution to EMBO reports and congratulations on a successful publication. Please consider us again in the future for your most exciting work.

Yours sincerely,

Achim Breiling
Editor
EMBO Reports

****

THINGS TO DO NOW:

Please note that you will be contacted by Wiley Author Services to complete licensing and payment information. The required 'Page Charges Authorization Form' is available here: https://www.embopress.org/pb-assets/embo-site/er_apc.pdf - please download and complete the form and return to embopressproduction@wiley.com

EMBO Press participates in many Publish and Read agreements that allow authors to publish Open Access with reduced/no publication charges. Check your eligibility: https://authorservices.wiley.com/author-resources/Journal-Authors/open-access/affiliation-policies-payments/index.html

You will receive proofs by e-mail approximately 2-3 weeks after all relevant files have been sent to our Production Office; you should return your corrections within 2 days of receiving the proofs.

Please inform us if there is likely to be any difficulty in reaching you at the above address at that time. Failure to meet our deadlines may result in a delay of publication, or publication without your corrections.

All further communications concerning your paper should quote reference number EMBOR-2023-57571V3 and be addressed to emboreports@wiley.com.

Should you be planning a Press Release on your article, please get in contact with emboreports@wiley.com as early as possible, in order to coordinate publication and release dates.

EMBO Press Author Checklist

Corresponding Author Name: Constantin Urban, Lucas Unger	
Journal Submitted to: EMBO Reports	
Manuscript Number: EMBOR-2023-57571V2	

USEFUL LINKS FOR COMPLETING THIS FORM
[The EMBO Journal - Author Guidelines]
[EMBO Reports - Author Guidelines]
[Molecular Systems Biology - Author Guidelines]
[EMBO Molecular Medicine - Author Guidelines]

Reporting Checklist for Life Science Articles (updated January
This checklist is adapted from Materials Design Analysis Reporting (MDAR) Checklist for Authors. MDAR establishes a minimum set of requirements in transparent reporting in the life sciences (see Statement of Task: 10.31222/osf.io/9sm4x). Please follow the journal's guidelines in preparing your
Please note that a copy of this checklist will be published alongside your article.

Abridged guidelines for figures
1. Data
The data shown in figures should satisfy the following conditions:
- the data were obtained and processed according to the field's best practice and are presented to reflect the results of the experiments in an accurate and unbiased manner.
- ideally, figure panels should include only measurements that are directly comparable to each other and obtained with the same assay.
- plots include clearly labeled error bars for independent experiments and sample sizes. Unless justified, error bars should not be shown for technical
- if n<5, the individual data points from each experiment should be plotted. Any statistical test employed should be justified.
- Source Data should be included to report the data underlying figures according to the guidelines set out in the authorship guidelines on Data

2. Captions
Each figure caption should contain the following information, for each panel where they are relevant:
- a specification of the experimental system investigated (eg cell line, species name).
- the assay(s) and method(s) used to carry out the reported observations and measurements.
- an explicit mention of the biological and chemical entity(ies) that are being measured.
- an explicit mention of the biological and chemical entity(ies) that are altered/varied/perturbed in a controlled manner.
- the exact sample size (n) for each experimental group/condition, given as a number, not a range;
- a description of the sample collection allowing the reader to understand whether the samples represent technical or biological replicates (including how many animals, litters, cultures, etc.).
- a statement of how many times the experiment shown was independently replicated in the laboratory.
- definitions of statistical methods and measures:
  - common tests, such as t-test (please specify whether paired vs. unpaired), simple χ2 tests, Wilcoxon and Mann-Whitney tests, can be unambiguously identified by name only, but more complex techniques should be described in the methods section;
  - are tests one-sided or two-sided?
  - are there adjustments for multiple comparisons?
  - exact statistical test results, e.g., P values = x but not P values < x;
  - definition of 'center values' as median or average;
  - definition of error bars as s.d. or s.e.m.

Please complete ALL of the questions below.
Select "Not Applicable" only when the requested information is not relevant for your study.

Materials

Newly Created Materials	Information included in the manuscript?	In which section is the information available? (Reagents and Tools Table, Materials and Methods, Figures, Data Availability Section)
New materials and reagents need to be available; do any restrictions apply?	Not Applicable	

Antibodies	Information included in the manuscript?	In which section is the information available? (Reagents and Tools Table, Materials and Methods, Figures, Data Availability Section)
For **antibodies** provide the following information:<br>- Commercial antibodies: RRID (if possible) or supplier name, catalogue number and or/clone number<br>- Non-commercial: RRID or citation	Yes	Materials and Methods

DNA and RNA sequences	Information included in the manuscript?	In which section is the information available? (Reagents and Tools Table, Materials and Methods, Figures, Data Availability Section)
**Short novel DNA or RNA including primers, probes**: provide the sequences.	Not Applicable	

Cell materials	Information included in the manuscript?	In which section is the information available? (Reagents and Tools Table, Materials and Methods, Figures, Data Availability Section)
**Cell lines:** Provide species information, strain. Provide accession number in repository **OR** supplier name, catalog number, clone number, and/**OR** RRID.	Not Applicable	
**Primary cultures:** Provide species, strain, sex of origin, genetic modification status.	Yes	Materials and Methods
Report if the cell lines were recently **authenticated** (e.g., by STR profiling) and tested for mycoplasma contamination.	Not Applicable	

Experimental animals	Information included in the manuscript?	In which section is the information available? (Reagents and Tools Table, Materials and Methods, Figures, Data Availability Section)
**Laboratory animals or Model organisms:** Provide species, strain, sex, age, genetic modification status. Provide accession number in repository **OR** supplier name, catalog number, clone number, **OR** RRID.	Not Applicable	
**Animal observed in or captured from the field:** Provide species, sex, and age where possible.	Not Applicable	
Please detail **housing and husbandry conditions**.	Not Applicable	

Plants and microbes	Information included in the manuscript?	In which section is the information available? (Reagents and Tools Table, Materials and Methods, Figures, Data Availability Section)
**Plants:** provide species and strain, ecotype and cultivar where relevant, unique accession number if available, and source (including location for collected wild specimens).	Not Applicable	
**Microbes:** provide species and strain, unique accession number if available, and source.	Yes	Materials and Methods, Table 1

Human research participants	Information included in the manuscript?	In which section is the information available? (Reagents and Tools Table, Materials and Methods, Figures, Data Availability Section)
If collected and within the bounds of privacy constraints report on age, sex and gender or ethnicity for all study participants.	Not Applicable	

Core facilities	Information included in the manuscript?	In which section is the information available? (Reagents and Tools Table, Materials and Methods, Figures, Data Availability Section)
If your work benefited from core facilities, was their service mentioned in the acknowledgments section?	Yes	Acknowledgements

Design

Study protocol	Information included in the manuscript?	In which section is the information available? (Reagents and Tools Table, Materials and Methods, Figures, Data Availability Section)

If study protocol has been **pre-registered, provide DOI in the manuscript**. For clinical trials, provide the trial registration number **OR** cite DOI.	Not Applicable	
Report the **clinical trial registration number** (at ClinicalTrials.gov or equivalent), where applicable.	Not Applicable	

Laboratory protocol	Information included in the manuscript?	In which section is the information available? (Reagents and Tools Table, Materials and Methods, Figures, Data Availability Section)
Provide DOI OR other citation details if **external detailed step-by-step protocols** are available.	Not Applicable	

Experimental study design and statistics	Information included in the manuscript?	In which section is the information available? (Reagents and Tools Table, Materials and Methods, Figures, Data Availability Section)
Include a statement about **sample size** estimate even if no statistical methods were used.	Yes	Materials and Methods
Were any steps taken to minimize the effects of subjective bias when allocating animals/samples to treatment (e.g. **randomization procedure**)? If yes, have they been described?	Yes	Materials and Methods
Include a statement about **blinding** even if no blinding was done.	Not Applicable	
Describe **inclusion/exclusion criteria** if samples or animals were excluded from the analysis. Were the criteria pre-established?<br><br>If sample or data points were omitted from analysis, report if this was due to attrition or intentional exclusion and provide justification.	Not Applicable	
For every figure, are **statistical tests** justified as appropriate? Do the data meet the assumptions of the tests (e.g., normal distribution)? Describe any methods used to assess it. Is there an estimate of variation within each group of data? Is the variance similar between the groups that are being statistically compared?	Yes	Materials and Methods, Figures

Sample definition and in-laboratory replication	Information included in the manuscript?	In which section is the information available? (Reagents and Tools Table, Materials and Methods, Figures, Data Availability Section)
In the figure legends: state number of times the experiment was **replicated** in laboratory.	Yes	Figures
In the figure legends: define whether data describe **technical or biological replicates**.	Yes	Figures

Ethics

Ethics	Information included in the manuscript?	In which section is the information available? (Reagents and Tools Table, Materials and Methods, Figures, Data Availability Section)
Studies involving **human participants**: State details of **authority granting ethics approval** (IRB or equivalent committee(s), provide reference number for approval.	Yes	Materials and Methods
Studies involving **human participants**: Include a statement confirming that **informed consent** was obtained from all subjects and that the experiments conformed to the principles set out in the WMA Declaration of Helsinki and the Department of Health and Human Services Belmont Report.	Yes	Materials and Methods
Studies involving **human participants:** For publication of **patient photos**, include a statement confirming that consent to publish was obtained.	Not Applicable	
Studies involving experimental **animals**: State details of **authority granting ethics approval** (IRB or equivalent committee(s), provide reference number for approval. Include a statement of compliance with ethical regulations.	Not Applicable	
Studies involving **specimen and field samples:** State if relevant **permits** obtained, provide details of authority approving study; if none were required, explain why.	Not Applicable	

Dual Use Research of Concern (DURC)	Information included in the manuscript?	In which section is the information available? (Reagents and Tools Table, Materials and Methods, Figures, Data Availability Section)
Could your study fall under dual use research restrictions? Please check biosecurity documents and list of **select agents and toxins** (CDC): https://www.selectagents.gov/sat/list.htm	Not Applicable	
If you used a select agent, is the security level of the lab appropriate and reported in the manuscript?	Not Applicable	
If a study is subject to dual use research of concern regulations, is the name of the **authority granting approval and reference number** for the regulatory approval provided in the manuscript?	Not Applicable	

Reporting

The MDAR framework recommends adoption of discipline-specific guidelines, established and endorsed through community initiatives. Journals have their own policy about requiring specific guidelines and recommendations to complement MDAR.

Adherence to community standards	Information included in the manuscript?	In which section is the information available? (Reagents and Tools Table, Materials and Methods, Figures, Data Availability Section)
State if relevant guidelines or checklists (e.g., **ICMJE, MIBBI, ARRIVE, PRISMA**) have been followed or provided.	Not Applicable	
For **tumor marker prognostic studies**, we recommend that you follow the **REMARK** reporting guidelines (see link list at top right). See author guidelines, under 'Reporting Guidelines'. Please confirm you have followed these guidelines.	Not Applicable	
For **phase II and III randomized controlled trials**, please refer to the **CONSORT** flow diagram (see link list at top right) and submit the CONSORT checklist (see link list at top right) with your submission. See author guidelines, under 'Reporting Guidelines'. Please confirm you have submitted this list.	Not Applicable	

Data Availability

Data availability	Information included in the manuscript?	In which section is the information available? (Reagents and Tools Table, Materials and Methods, Figures, Data Availability Section)
Have **primary datasets** been deposited according to the journal's guidelines (see 'Data Deposition' section) and the respective accession numbers provided in the Data Availability Section?	Yes	Figures, BioStudies
Were **human clinical and genomic datasets** deposited in a public access-controlled repository in accordance to ethical obligations to the patients and to the applicable consent agreement?	Not Applicable	
Are **computational models** that are central and integral to a study available without restrictions in a machine-readable form? Were the relevant accession numbers or links provided?	Not Applicable	
If publicly available data were reused, provide the respective **data citations in the reference list**.	Not Applicable	
